# The Distribution and Biogenic Origins of Zinc in the Mineralised Tooth Tissues of Modern and Fossil Hominoids: Implications for Life History, Diet and Taphonomy

**DOI:** 10.3390/biology12121455

**Published:** 2023-11-21

**Authors:** M. Christopher Dean, Jan Garrevoet, Stijn J. M. Van Malderen, Frédéric Santos, Marta Mirazón Lahr, Robert Foley, Adeline Le Cabec

**Affiliations:** 1 Centre for Human Evolution Research, Natural History Museum, Cromwell Road, London SW7 5BD, UK; 2 Department of Cell and Developmental Biology, University College London, Gower Street, London WC1E 6BT, UK; 3 Deutsches Elektronen-Synchrotron DESY, Notkestraße 85, 22607 Hamburg, Germany; jan.garrevoet@desy.de (J.G.); stijn.vanmalderen@teledyne.com (S.J.M.V.M.); 4 Univ. Bordeaux, CNRS, Ministère de la Culture, PACEA, UMR 5199, F-33600 Pessac, France; frederic.santos@u-bordeaux.fr (F.S.); adeline.le-cabec@u-bordeaux.fr (A.L.C.); 5 Leverhulme Centre for Human Evolutionary Studies, Department of Archaeology, University of Cambridge, Fitzwilliam Street, Cambridge CB2 1QH, UK; mbml1@cam.ac.uk (M.M.L.); raf10@cam.ac.uk (R.F.)

**Keywords:** trace elements, neonatal line, enamel, SXRF, enamel thickness, mineralisation process

## Abstract

**Simple Summary:**

Teeth begin to grow in the jaws before birth and continue to appear in an overlapping sequence until each is complete in length. Subsequently, the central pulp (nerve) chamber of each tooth slowly fills in with dentine and the root of the tooth continues to accumulate thin layers of cementum until the individual dies. Each of the tooth tissues, the hard enamel cap, the dentine core of the tooth and the root cementum grow incrementally and incorporate small quantities of blood-born trace elements ingested from our diet into their structure. A chronological record of zinc incorporation exists in each tooth tissue and can be visualised in thin sections, or slices, of teeth using a beam of synchrotron light. Zinc markings in teeth are especially useful and occur at birth in enamel and dentine and annually in the cementum layers. This work shows that zinc is consistently concentrated within surface enamel and in the dentine surrounding the central pulp chamber. Knowing where to sample Zn in modern and fossil teeth enables us to reconstruct a chronology of growth and to determine something about diet in the past from the remnants of different Zn isotopes contained in different foodstuffs.

**Abstract:**

Zinc is incorporated into enamel, dentine and cementum during tooth growth. This work aimed to distinguish between the processes underlying Zn incorporation and Zn distribution. These include different mineralisation processes, the physiological events around birth, Zn ingestion with diet, exposure to the oral environment during life and diagenetic changes to fossil teeth *post-mortem*. Synchrotron X-ray Fluorescence (SXRF) was used to map zinc distribution across longitudinal polished ground sections of both deciduous and permanent modern human, great ape and fossil hominoid teeth. Higher resolution fluorescence intensity maps were used to image Zn in surface enamel, secondary dentine and cementum, and at the neonatal line (NNL) and enamel–dentine–junction (EDJ) in deciduous teeth. Secondary dentine was consistently Zn-rich, but the highest concentrations of Zn (range 197–1743 ppm) were found in cuspal, mid-lateral and cervical surface enamel and were similar in unerupted teeth never exposed to the oral environment. Zinc was identified at the NNL and EDJ in both modern and fossil deciduous teeth. In fossil specimens, diagenetic changes were identified in various trace element distributions but only demineralisation appeared to markedly alter Zn distribution. Zinc appears to be tenacious and stable in fossil tooth tissues, especially in enamel, over millions of years.

## 1. Introduction

Zinc is an essential biological trace element. Besides this, Zn also has an affinity for bone and the three mineralised tooth tissues, enamel, dentine and cementum. Not only does the distribution of Zn vary among the three tooth tissues, but its origins also derive from different physiological, developmental and chemical processes. Ultimately, the Zn source is either from the intestinal absorption of ingested dietary Zn, or from the mother perinatally, via a direct-blood-borne maternal–placental transfer to the fetus. Zinc retrieved from tooth tissues in an archaeological and palaeontological context offers strong potential for revealing aspects of an individual’s life history, for example, as a marker of birth or of annual increments of tissue laid down during life, or as an indicator of diet. Reviewing the role and function of Zn in the body, and of how it comes to distribute differently in each of the tooth tissues, provides some insight into how and where it might best be sampled and of where its incorporation into each of the mineralised tooth tissues is likely to be tightly chronologically circumscribed or not. Fossil tooth tissues are often, if not always, altered through diagenesis, and a comparison of Zn distribution and preservation in fossils from contrasting sites may point to similarities or differences with modern teeth and so indicate where within the tooth tissues Zn of biogenic origin persists for longest. 

### 1.1. Zinc as an Essential Trace Element in the Body

Zinc is an essential trace element involved in many physiological processes as both a catalyst in biochemical reactions, in the maintenance of protein quaternary structure and as a component of many essential enzymes [1]. Zinc is required, among other things, for cell division, tissue growth and wound healing, but also for intestinal electrolyte absorption, neurotransmission, the immune response, thymus activity and vision [1,2]. It is the most abundant trace element found in bone mineral, being present in concentrations of between 200–300 ppm [2,3]. Zinc is also present in muscle, red blood cells and skin as well as in bone and the mineralised tooth tissues [2,4]. Zinc is required for the DNA binding proteins involved in regulating gene transcription and expression [5]. It is also a critical component of several hundred essential enzymes and proteins [4]. At least 10% of human proteins contain Zn as a cofactor [6]. These include many metalloproteins and enzymes that are involved in most of the major metabolic pathways [1]. 

Among metalloenzymes, Zn is a component of carbonic anhydrase, alcohol dehydrogenase [7] and of alkaline phosphatase that is crucially involved in the process of mineralisation through the generation of free phosphate groups that are then taken up by newly forming bone and tooth tissues [8,9,10,11]. The zinc-finger proteins that control a variety of fundamental cellular activities require Zn to maintain their precise quaternary structure and functional integrity [1]. Some zinc-finger proteins have been identified as specific regulators of skeletal development and mineralised tissues formation [5,11]. Zinc in bone is concentrated at the sites of mineralisation and has been shown to stimulate osteoblast differentiation and proliferation [9,10,12]. Kim et al. [13] have also demonstrated that a Zn finger-containing transcription factor (Osterix; Osx) is an essential site-specific regulator of odontoblast differentiation, maturation and of tooth root elongation.

### 1.2. Zinc in Enamel Secretion and Maturation

Matrix metalloproteinase 20, or MMP-20, (sometimes also referred to as enamelysin), is a protease that is secreted with enamel proteins during the early (secretory) stage of amelogenesis, when the enamel crystallites are growing predominantly in length [11,14]. MMP-20 cleaves the enamel proteins surrounding crystallites to allow their slow growth in width, after which these cleavage products are reabsorbed by secretory ameloblasts and degraded. The more aggressive serine protease, kallikrein 4, or KLK-4, is a protease that is secreted during the later transition and maturation stages of amelogenesis. KLK-4 degrades what remains of the enamel organic matrix after enamel secretion is completed [11,14]. The principal functions of MMP-20 and KLK-4 in dental enamel formation are to facilitate the orderly replacement of organic matrix with mineral to form hard dense enamel. This occurs in a low-calcium environment that favours the slow controlled formation of hydroxyapatite that is maintained by the tight barrier filter of the secretory ameloblast sheet; this excludes or removes excess Ca [15]. MMP-20 itself contains Zn, but Zn in enamel is a potent inhibitor of serine proteases and so may have an important controlling influence, especially on KLK-4 during the maturation phase [11]. For these reasons, it has been suggested that Zn enrichment in the outer enamel [16,17], but also at the EDJ [18] and in dentine [19], may result from metalloprotease activity and degradation that results in the retention and sequestration of Zn originally involved in the mineralisation and/or enamel maturation process. 

### 1.3. Zinc Absorption and Maternal–Fetal Transfer

Zinc absorption from the gut involves first binding to a surface receptor on the gut wall enterocytes and then subsequently being taken up into the enterocytes [10,20]. Zn has five stable isotopes of which the most abundant are ^64^Zn and ^66^Zn. The detection and distribution of Zn isotopes in dental tissues and their relation to diet are discussed more fully below (Section 1.6). However, the intestinal absorption of Zn is inhibited and undergoes isotopic fractionation by, for example, plant phytates which precipitate dietary Zn, and because there is a preferential precipitation of the lighter Zn isotopes (^64^Zn) with plant phytates, this then favours heavier ^66^Zn absorption and enrichment relative to ^64^Zn [21]. Thus, the isotopic composition of Zn found in body tissues may not directly reflect the dietary isotopic composition [21,22]. A proportion of absorbed Zn then remains bound to metallothionein within the enterocyte, and as this accumulates, it inhibits further Zn absorption from the gut, thus regulating Zn uptake. It follows that as dietary Zn intake increases beyond the threshold required, Zn absorption decreases relative to intake [20]. The proportion of Zn that remains bound to metallothionein within the cell, eventually returns to the bowel lumen when the enterocyte is shed. Consequently, faecal loss of unabsorbed Zn increases with excess Zn uptake as the Zn-containing enterocytes are shed. Zn is also lost through urinary excretion and through sweat; this has practical implications when performing Synchrotron X-ray fluorescence (SXRF) experiments to map Zn distribution in tooth and bone samples, as surfaces under investigation must remain completely clean and untouched. Zn is an important essential metal ion such that recent research has identified metallochaperone proteins that escort or direct Zn to specific crucial cellular enzymes, ensuring the correct Zn allocation when levels are scarce [6]. 

Some Zn becomes bound in serum to albumin or an alpha-2 macroglobulin after absorption; this may be transported to the liver [2,20], but beyond the Zn component in bone mineral, there are no real functional reserves of Zn in the body [1]. The exception is in neonates where, at term, Zn accumulated during gestation is stored in the liver and then released postnatally. Most Zn is transferred to the fetus from the mother after the 24th week of gestation and stored in the fetal liver [1]. Pre-term birth, therefore, reduces the amount of Zn that can accumulate with the risk of Zn deficiency in the neonate [1,23]. Maternal-fetal placental transfer of Zn is an active process and fetal Zn concentrations are maintained constantly higher than maternal levels. While Zn in human milk varies in concentration between 0.7–1.6 mg/L, and declines with time, colostrum contains 8–12 mg/L, falling to 3–6 mg/L within a week [1].

### 1.4. The Distribution of Zinc in Mineralised Tissues

**Figure 1:**
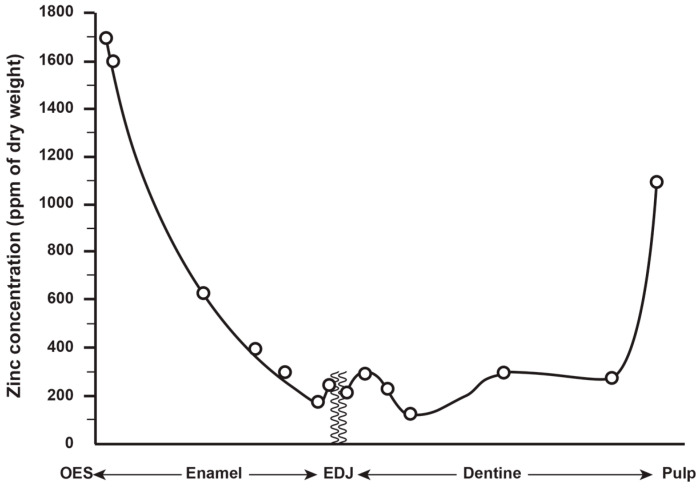
The distribution and concentration (ppm) of Zn from the outer enamel surface (OES) to the EDJ (enamel–dentine junction) and from the EDJ to the pulp in crowns of unerupted teeth collected from Schenectady, New York. Adapted from Figure 1 in Brudevold et al. [7].

Zinc is the only trace element in teeth where the concentrations approach that of fluoride, an element with which Zn shares a similar distribution across the various tooth tissues [7,24]. Brudevold et al. [7] used spectrography on serial sections of teeth to describe the distribution and concentration of Zn in unerupted and erupted human permanent teeth from patients of known age and of diverse geographical origin. They reported the highest concentrations of Zn in the outer surface layers of enamel (1300–2100 ppm) and quantified a steep decline in Zn concentration towards the enamel dentine junction (EDJ) where it was ~200 ppm (Figure 1). Brudevold et al. [7] conclude that most of the Zn uptake occurred prior to eruption, as attested by the high levels in Zn in the unerupted teeth. They further state that, although some Zn uptake may occur, the post-eruptive data are too irregular to support a continuous Zn uptake with age [7]. Yet, there was a variation in Zn concentration between the teeth of diverse worldwide origins where outer enamel values ranged between 900–2100 ppm [7]. More recently, Lynch [4] has shown that post-eruptive Zn uptake can occur and may, for example, be derived from accumulated Zn in the salivary pellicle and oral mucosa. In modern teeth, it is also possible, but remains to be demonstrated, that further Zn enrichment may occur at the very outer enamel surface following a prolonged use of dentifrices, to which Zn is added as an anti-bacterial agent to control plaque accumulation and reduce calculus formation [4].

Other studies have also reported a gradient of Zn concentration in enamel with maximal values in outer enamel (e.g., [25,26,27]). Sanchez-Quevedo et al. [28] noted a rise in the Zn gradient from inner to outer enamel, and Humphrey et al. [29,30,31] and Müller et al. [17] have shown that Zn/Ca ratios in deciduous enamel increase near exponentially, some 20–30 times [17], towards the outermost enamel layers from values close to the EDJ using laser ablation-inductively coupled plasma-mass spectrometry (LA-ICP-MS). In both a modern human sample and in a sample of mostly thin-enamelled, non-primate fossil taxa (but including one *Macaca* sp.), Bourgon et al. [32] reported the same gradient of Zn distribution where the range of maximum concentrations in outer enamel was between ~100–700 ppm in both samples, and Brozou et al. [26] also reported the Zn concentration within the “first tens of microns of the enamel surface” as ~650 ppm. Just one study, however, based on atomic absorption spectrometry of permanent upper central incisor labial enamel, reported a reverse pattern of Zn enrichment with lower concentrations at the surface enamel and higher concentrations at the EDJ [11].

Kang and colleagues [33] again used LA-ICP-MS and reported high concentrations of Zn at the enamel neonatal line (NNL) and, since then, Dean et al. [34] have used synchrotron X-ray fluorescence (SXRF) to quantify Zn concentration at the NNL and the enamel-dentine junction (EDJ), where it was between 200–500 ppm, compared with Zn concentration at the outer deciduous enamel surface, where it was slightly higher (400–500 ppm). 

In dentine, Brudevold et al. [7] reported that the greatest concentration of Zn (as high as 1000 ppm) was in secondary dentine, adjacent to the pulp chamber, with a decreasing gradient in concentration towards the EDJ in the crown and towards the cementum–dentine junction (CDJ) in the root dentine (Figure 1; see also [25,26]). Noticeably, there was a small rise in Zn concentration (of between 150–300 ppm) in the mantle dentine immediately adjacent to the EDJ, and Dean et al. [34] have also reported Zn concentrations of up to 500 ppm along the EDJ of deciduous teeth. Stock et al. [35] and Dean et al. [36] have each identified high concentrations of Zn in slow forming peritubular dentine. Kang and colleagues [33], using LA-ICP-MS again, identified elevated levels of Zn at the dentine NNL, along the EDJ and in secondary dentine adjacent to the pulp. Studies of deciduous dentine using SXRF [34] have also identified high levels of Zn in the dentine NNL itself, but also in dentine formed prenatally.

**Figure 2:**
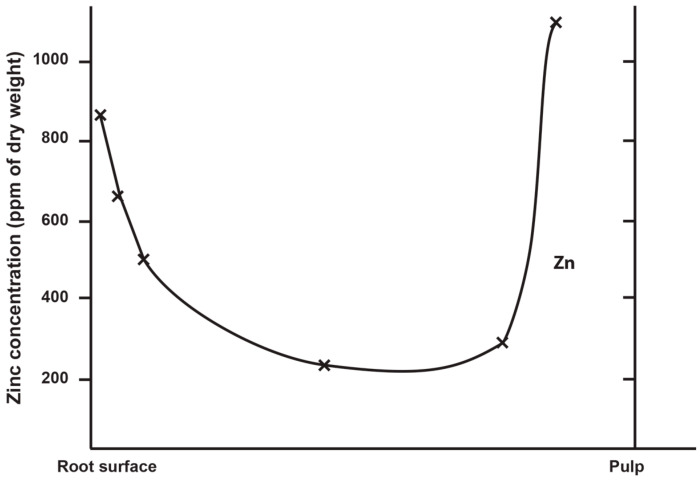
The distribution and concentration (ppm) of Zn from the tooth root surface to the pulp in unerupted teeth collected from Schenectady, New York. Adapted from Figure 2 in Brudevold et al. [7].

At the tooth root surface, Brudevold et al. [7,37] first reported that the Zn concentration in cementum was also high, ranging from 500 to 1500 ppm; subsequently, Martin et al. [38,39] also demonstrated high levels of Zn in cementum as compared with the underlying root dentine (Figure 2). Since this time, several papers have observed and quantified the distribution and concentration of Zn in both cellular and acellular cementum [19,26,36,40,41]. At higher resolution, it becomes clear that there are fine Zn-rich lines or bands in cementum that define what have been shown to be annual incremental markings (see [42] for a review; see e.g., [43,44] for further visualisation of these incremental markings) with greater resolution than transmitted light microscopy or than SXRF intensity maps for Ca or Sr [26,36].

Zinc is also present in bone at the boundary between the forming pre-calcified and calcified bone matrix where it is associated with alkaline phosphatase, a metalloenzyme that itself contains two functional Zn atoms. However, unlike dentine or cementum, Zn does not persist in bone [12] but may well play an important role in stimulating bone cell proliferation and collagen formation [9,41,45]. These observations about Zn at the bone-forming front of Haversian systems have prompted suggestions that the Zn-rich incremental markings in cementum may also reflect periods of cell proliferation and initiation of cementoblast secretory activity [36].

### 1.5. Zinc Incorporation into Hydroxyapatite

Brudevold et al. [7] and Lynch [4] have proposed that Zn is likely to be incorporated directly into the hydroxyapatite crystal lattice during crystal formation as well as at the crystal surface (depending on the Zn/Ca ratio present in the enamel fluid). In enamel, Zn also continues to be incorporated into hydroxyapatite during further mineral deposition during the pre-eruptive phase by exchange of Ca^2+^ and Zn^2+^, and then subsequently through the post-eruptive acquisition of Zn by surface enamel from saliva, plaque, water and food [4]. However, Zn also readily binds to proteins present in the organic components of dental tissues, which make up a much greater proportion of mature dentine and cementum than enamel. Raising dietary levels of Zn does not seem to raise levels of Zn measured in teeth [46], suggesting there is tight regulation of Zn (and other ion) levels by secretory ameloblasts [47]. If this is indeed the case, it begs the question of how the observed variation in Zn concentration in both forming enamel and dentine can arise in the first place. It implies that fluctuations in Zn concentration would result largely from periods of Zn depletion that fall below a threshold maximum rather than from bouts of Zn enrichment.

High levels of Zn in enamel, secondary dentine and cementum may be adaptive in that it reduces acid solubility and inhibits the resorption of cementum by osteoclasts and thus protects the root surface [3,36,48,49,50,51,52]. When the solution concentration of Zn^2+^ ions at the hydroxyapatite crystal surface exceeds 1 ppm [53], the precipitation of a zinc phosphate, alpha-Hopeite, (α-Zn_3_(PO_4_)_2_.4H_2_O), occurs [4]. Mohammed et al. [50] have demonstrated that the likely mechanism for the reduction in the acid dissolution of hydroxyapatite is through Zn interacting and binding with PO_4_^3−^ sites at the crystal surface where it forms a Hopeite-like phase, suppressing the release of further phosphate ions and reducing mineral loss from the apatite structure at low pH. While Zn certainly reduces the acid solubility of hydroxyapatite, to a similar degree as fluoride, it appears to have little or no effect in reducing the incidence of caries [4,7]. Nonetheless, Zn has been shown to selectively and strongly inhibit osteoclast activity in bone resorption [3]. Similarly, at the cementum surface, Zn may well inhibit osteoclast activity, and cementum resorption and Zn at the inner aspect of the pulp chamber may well also protect dentine from resorption by blood-borne osteoclasts present in the pulp [36]. The presence of Zn^2+^ ions is also effective in inhibiting hydroxyapatite crystal growth [53] and so Zn may well play a role in controlling the rate of crystallite growth in enamel, dentine and cementum formation. It is for this reason that Zn is an effective inhibitor of calculus formation in the mouth and why, therefore, as well as being bacteriostatic, it is incorporated into so many dental health products [4].

### 1.6. Zinc Isotopes and Diet in Fossils

Zinc concentration itself is not a reliable dietary trophic indicator; however, the Zn isotopic fingerprint in tooth tissues has been shown to provide information about diet, and the two most abundant Zn isotopes, ^64^Zn and ^66^Zn, are yielding important dietary information in modern and fossil taxa. New methods of detecting stable isotopes (multi-collector inductively coupled plasma mass spectrometry, or MC-ICP-MS) has meant that it is now possible to detect stable isotopes in modern and fossil tooth tissues at high-resolution [10,32,54,55,56,57,58]. This has proved to be a breakthrough in reconstructing reliable diet-related trophic levels in mammal teeth from specific sites and locations using the ratio of ^66^Zn/^64^Zn expressed as the δ^66^Zn value [21,32,55,56,57,58,59,60]. Food ingested has different δ^66^Zn values depending on its origins. For example, the plants that herbivores eat have the most elevated δ^66^Zn values and the muscle tissues that many carnivores consume have the lowest δ^66^Zn values [32]. It follows that the mineralised tooth tissues of carnivores are typically depleted in the heavier Zn isotope and are approximately 0.4–0.5‰ lower in their ^66^Zn/^64^Zn ratio compared to herbivores from the same location and food web [55]. The fact that the dietary expectations of fossil taxa can be accurately reconstructed from the δ^66^Zn values in tooth enamel, and that they closely reflect those known in modern taxa, strongly suggests fossil teeth preserve their original isotopic composition [32]. Moreover, Zn remains distributed and concentrated in the outer 10% (~200 µm) of cuspal enamel in many thin-enamelled fossil species (See SI Figure S30 on page 38 in [32]) in the same way as reported for modern teeth. However, in vitro alteration experiments where enamel and dentine were placed in enriched ^67^Zn isotopic solutions for different periods of time have demonstrated that the Zn isotopic composition in outer enamel can change over time while the inner enamel remains unaffected [61]. On this basis, Weber et al. [61] have suggested sampling enamel at least 100–200 µm deep to the surface to avoid potential diagenetic changes in fossil material. Dentine, in these experiments, was, as expected, judged to be far more prone to diagenetic isotopic alteration than enamel, even close to the EDJ [61].

It seems likely that the dietary transition from mother’s milk through the introduction of solid foods and then onto an exclusively post-weaning plant or cereal-based diet is reflected in the δ^66^Zn value detected in tooth enamel forming at different times from childhood to adulthood [22]. In two modern archaeological samples, there was a trend of decreasing δ^66^Zn value with increasing crown formation age through the deciduous then permanent molar series, dm1, dm2, M1, M2, M3, and this has been attributed to changes in the intestinal absorption and fractionation of ingested Zn with age [21,22].

### 1.7. Aims of the Study

This study aimed to address three research questions that each concern the distribution and concentration of Zn as a trace element in extant and fossil primate tooth tissues. First, the distribution of Zn was mapped in pre- and postnatal enamel and dentine to explore the degree to which Zn distribution in dental tissues reflects rising prenatal fetal serum Zn levels in the last trimester and Zn-rich colostrum in the first postnatal days in breast-fed infants. Second, unerupted teeth were included in this study for comparison with teeth that had been in function in the oral environment. The aim was to identify any marked differences in Zn concentration and distribution in the outer enamel that might derive entirely from the oral environment, or alternatively be incorporated during the pre-eruptive enamel formation period. Third, the distribution and concentration of Zn was mapped in fossil hominoid and hominin teeth from different geological contexts to identify any consistent Zn-rich regions that compared with modern material. Patterns of diagenetic change in Ca, Zn, Sr, Fe and Mn were also mapped in each of the fossil tooth tissues, to compare the stability of each of these elements with that of Zn and to identify any potential influence they might have on the regional distribution of Zn over long periods of time.

## 2. Materials and Methods

### 2.1. Samples

The sample of teeth used in this study is summarised in Table 1 and included extant teeth attributed to modern humans (9 deciduous teeth and 3 permanent teeth), *Pan* (5 permanent teeth), *Gorilla* (2 deciduous and 3 permanent teeth) and *Pongo* (2 deciduous teeth and one permanent tooth). The deciduous and permanent modern human teeth used in this study have been described and used in previous studies [34,62,63]. The origins of the deciduous and permanent great ape teeth have also been described previously [64], and some have been used in previous SXRF studies [65]. All non-human teeth derive from once-free-living individuals collected ~100 years ago.

Fossil teeth from five genera were included in this study, all of which have previously been described and published. These included, *Ekembo nyanzae*, a URM1, KNM-RU 1721; *Ekembo heseloni*, a LRdm2 and an LLM1, both from KNM-RU individual IV recovered from the 17.4–17.8 Ma site on Rusinga Island, Kenya and previously described in Beynon et al. [66]; *Hispanopithecus laietanus*, IPS 1781, an UM1 from the Late Miocene site, Can Llobateres, Spain, previously described in Dean and Kelley [67]; *Australopithecus anamensis*, KNM-KP 30748, a mandibular premolar fragment from the 3.9–4.2 Ma site at Kanapoi, Kenya, previously described in Ward et al. [68]; *Paranthropus robustus*, a third permanent molar SK 835 from the 1.5–1.8 Ma site at Swartkrans, South Africa, previously described in Dean et al. [69]; a Neanderthal Udm1 belonging to the infant Amud 7, from the ~60 kyr [70] cave site, Amud, Israel, previously described by Rak et al. [71] and Hovers et al. [72]; and a single fossil LLM3 from the early modern human Skhūl IV specimen, from the 90–120 kyr [73,74] site at Mount Carmel, Israel, previously described in Dean et al. [75].

In this study, we have adopted the tooth notation system commonly used by dental anthropologists: U = upper (maxillary), L = lower (mandibular); followed by L = left and R = right; followed by tooth type (upper case) permanent, (lower case) deciduous; followed by tooth number. For example, LRM2 = lower right second permanent molar; URdm1 = upper right deciduous first molar. When the side and/or jaw is unknown just the tooth type appears.

### 2.2. Histological Methods

The modern and fossil teeth used in this study were sectioned longitudinally with a low-speed diamond saw (Buehler IsoMet). One cut face was then abraded using a graded series of abrasive papers and polished with a 3-μm aluminium oxide polishing powder and deionised water on a polishing pad. This polished surface was then cleaned in an ultrasonic bath and dried and fixed to a 1 mm-thick glass slide with zero-bond epoxy resin (Huntsman Araldite 2020) subjected to pressure for 48 h. A further cut was then made parallel with the glass slide and tooth block leaving a 300–400 μm thick longitudinal tooth section and a thin layer of epoxy bond attached to the slide. Sections were then ground and lapped to between 80–100 μm above of the glass slide surface and polished according to the protocol above. When sections were prepared specifically for this study, no coverslip was placed leaving the polished surface exposed for SXRF. Sections that had been previously prepared for other studies that already had coverslips placed with DPX clearing medium were soaked in xylene for 24 h and the coverslips were removed. After polishing, all sections were cleaned ultrasonically in deionised water, but no etching or surface treatment of any kind was carried out.

### 2.3. SXRF Methods

Synchrotron X-ray fluorescence (SXRF) methods adopted in this study have previous been described in detail elsewhere [26,36,65] but are briefly summarised here. Polished thin ground sections of teeth (~80–100 µm) were mapped to quantify the distribution and concentration of Zn in enamel, dentine and cementum. SXRF experiments were performed on the P06 Beamline [76,77], Petra III, at DESY (Deutsches Elektronen-Synchrotron, Hamburg, Germany). Data were acquired during three experiments at P06, using a Maia detector system [78] or two Vortex EM silicon drift detectors, Hitachi High-Tech Science America, Inc. (Chatsworth, CA, USA). The scanning strategy followed a multiscale approach for each tooth. Teeth were scanned several times at increasing resolution with an X-ray beam monochromatised to 16.5 or 17.0 keV, depending on the monochromator tuning, at resolutions ranging between 25 µm and 1 µm and integration time ranging from 4 to 10 ms. First, a fast overview scan with a step size of 200 or 100 µm (dwell time 10 ms) was acquired to verify that the tooth section was well-centred in the field of view and to assess the overall signal-to-noise ratio of the elemental signature. Second, a higher resolution overview scan (15–25 µm; integration time: 10 ms) was performed to visualise the elemental variation within the smaller region of interest. Finally, based on prior observations of the tooth sections under transmitted light microscopy and on the SXRF signal quality in the 10 µm scans, some small regions of interest were selected and scanned at either 5.0, 2.5 or 1.0 µm (See Table 1 for details).

The X-ray yield calculations were performed assuming a hydroxyapatite matrix (Ca_10_(PO_4_)_6_(OH)_2_) with density ~3.0 g/cm^3^ close to enamel [79] and a final sample thickness of 80–120 μm (measured for each specimen, see Table 1). Glass slides were included in the overall sample absorption model as appropriate. Only three thin sections were fixed on a kapton foil, and which were accounted for in the calculations. Spectral peak deconvolution and integration was performed using the core of PyMca 5.5.0 and PyMca 5.5.3 for data acquired with the Vortex detectors [80]. Concentrations were determined using a conversion factor (photon counts to equivalent charge) through measurement of a standard Ni foil with areal density 50.0 µg/cm^2^ (Micromatter Technologies Inc., Surrey, BC, Canada) and additional 6.3 µm foils (Mylar) of CaF_2_, 20.1 μg/cm^2^, ZnTe, 50.5 μg/cm^2^, and SrF_2_, 48.7 μg/cm^2^. Elemental distribution maps were normalised to the incoming X-ray flux. SXRF concentrations are reported as parts-per-million (ppm or μg/g). The elemental maps were generated in GeoPIXE 7.4f. or in HDIP v-1.3.3.1073 (Teledyne Photon Machines, Bozeman, MT, USA). Using ImageJ (version 1.53m), raw data were collected along Regions of Interest (ROIs; see Appendix SA, Table S1), and selected maps were adjusted for better revealing the targeted dental structures for the figures.

### 2.4. Statistical Methods

Graphs and plots were performed in Excel, and PAST v4.13 [81]. All the statistical analyses for investigating the correlations were performed using R [82]. Appendix SB was built with Org mode 9.6.10 for GNU Emacs 29.1 [83]. Along with R version 4.3.1 (16 June 2023) itself, the R packages used in Appendix SB are loaded, using their version available on CRAN at a fixed date (22 September 2023), using the R package {groundhog} [84]. Further statistical methods are set out in Appendix SB.

## 3. Results

### 3.1. Zinc Distribution in Modern Human and Great Ape Deciduous Teeth

**Figure 3:**
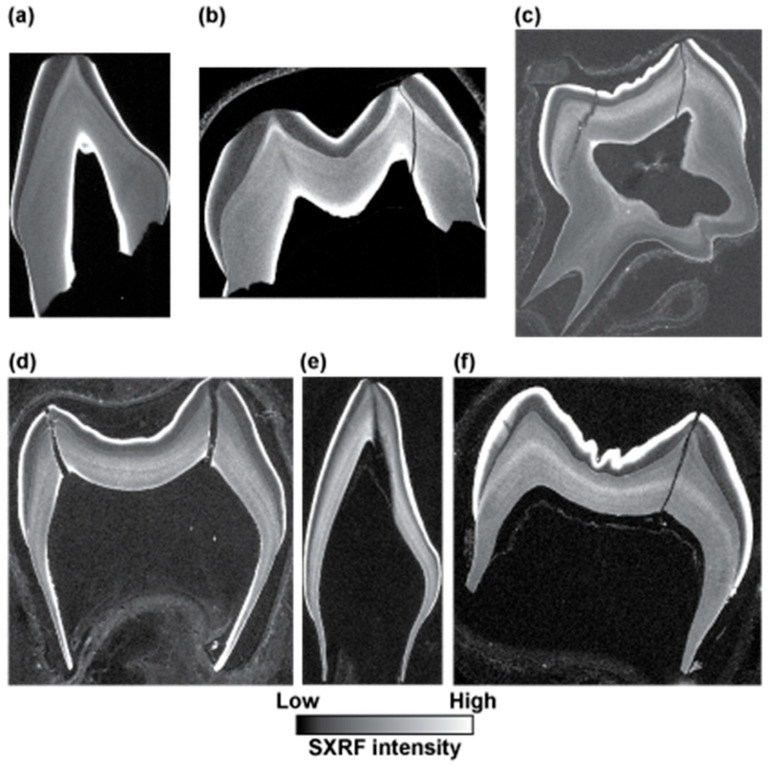
SXRF intensity maps for Zn in (**a**) modern human Ldc, (**b**) modern human Udm2 from the same individual as (**a**), (**c**) *Pongo* Udm2, (**d**) *Gorilla* Ldm2, (**e**) *Gorilla* Ldc from the same individual as (**d**,**f**) *Pongo* Ldm2 from a different individual to (**c**). In all cases, outer enamel as well as secondary dentine and cementum (when formed, i.e., in (**a**–**c**)) are Zn-rich. In modern human teeth, prenatal dentine is Zn-rich relative to dentine formed postnatally. In the modern great ape teeth, the reverse is true. All teeth show Zn distribution in dentine that broadly follows the incremental growth pattern of the tooth. Teeth on the first row are worn and have been in functional occlusion (emerged), while the teeth on the bottom row are unerupted (and thus unworn) and were completely contained within their crypts. Images (**a**,**b**) are from Dean et al. [34] Figure 1. Images are not to scale.

Figure 3 shows the distribution of Zn in two modern human deciduous teeth from the same individual (Ldc and Udm2), two *Gorilla* deciduous teeth from the same individual (LRdm2 and LLdc) and two *Pongo* deciduous teeth from different individuals (URdm2 and LRdm2). In all of these deciduous teeth, the outer enamel is Zn-rich. Where enamel is worn, there is no evidence that Zn from the oral environment has been incorporated into newly exposed enamel. Where secondary dentine or cementum has formed, these tissues are Zn-enriched. The bulk of the dentine in all teeth shows variation in the Zn SXRF intensity that broadly follows the incremental pattern of dentine formation. Both modern human teeth show Zn enrichment in the first formed prenatal dentine that corresponds with the first formed prenatal cuspal enamel; however, this is not the case in any of the great ape deciduous teeth, where, if anything, prenatal enamel appears Zn-depleted relative to the dentine formed postnatally. Despite this, at higher resolution, a Zn-rich NNL is often discernible. In all teeth, bands of Zn-rich dentine alternate with regions of relative Zn-depletion. 

**Figure 4:**
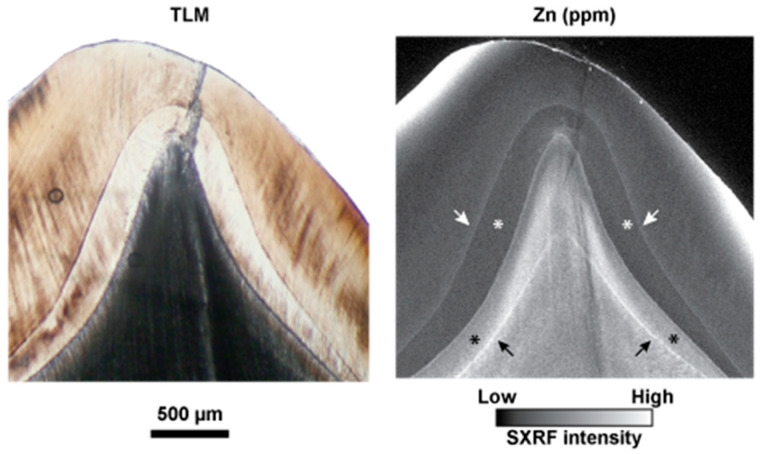
Transmitted light micrograph (TLM) and matching Zn SXRF intensity image of the cuspal region of a modern human dm2 showing the neonatal line (NNL) and EDJ. In the Zn map, the NNL in both enamel (white arrows) and dentine (black arrows) is Zn-rich, as is the prenatal dentine (black asterisks) and EDJ. In prenatal enamel (white asterisks), the SXRF intensity map suggests that this is Zn-depleted relative to postnatal enamel. Adapted from Figure 2e,h in Dean et al. [34].

**Figure 5:**
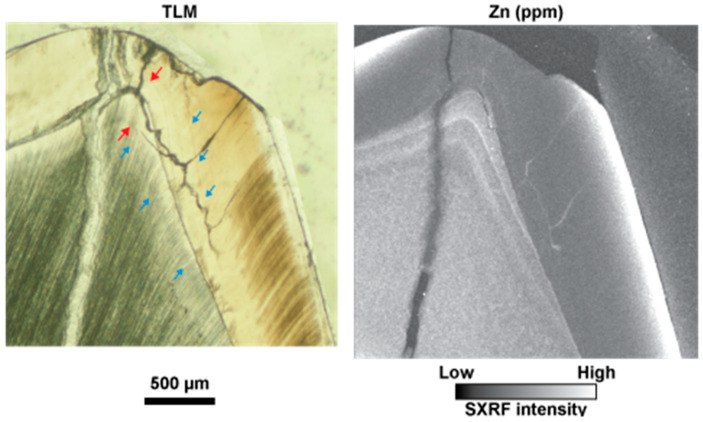
Transmitted light micrograph and matching Zn SXRF intensity image of the cuspal region of a modern human Udm2 showing the neonatal (NNL) and EDJ from an individual born ~6 weeks prematurely by Caesarean section. A faint NNL is visible in the light micrograph (red arrows) as well as a stress line (at 57 days after birth; blue arrows) more clearly observed in enamel. In the SXRF map, the unworn surface enamel, EDJ, the NNL in both enamel and dentine and the stress line in dentine are Zn-rich. The prenatal enamel and dentine formed after the NNL, and the stress line all appear to be Zn-depleted.

Figure 4 shows the cuspal region of a modern human second deciduous molar at higher resolution [34]. The NNL is clearly visible when imaged with transmitted light microscopy in enamel but is difficult to observe in dentine. The SXRF intensity map of the same cusp, however, clearly shows a Zn-rich NNL in both enamel and dentine as well as Zn enrichment along the EDJ and in the dentine formed prenatally. The enamel formed prenatally, in contrast, is depleted in Zn. Figure 5 shows how this general pattern of Zn distribution may vary. Here, the cusp of an Udm2 from a child of known history, and previously described by Birch and Dean [85], is imaged with transmitted light microscopy and then mapped with SXRF. The child, a twin that was judged to be in distress and of low birth weight for its gestational age, was born ~6 weeks prematurely by Caesarean section.

The NNL in both enamel and dentine is only faintly visible in the transmitted light image and is close to the dentine horn (compare with the position of the NNL in the same tooth type in Figure 3). The SXRF intensity map for Zn shows a clear NNL in dentine and a fainter NNL in enamel. The prenatally formed dentine, but not enamel, is also Zn-rich. Following gastrointestinal upsets between days 35–40 postnatally, the child underwent surgery under a general anaesthetic on day 57 for an acute intestinal hernia [85]. A faint accentuated stress marking associated with this incident is just visible in the enamel and dentine in the transmitted light image (Figure 5, blue arrows). In the SXRF image, however, a Zn-rich line in the dentine clearly marks this acute stress event. A matching line in enamel is, however, almost imperceptible. The dentine formed after this stress event then appears relatively Zn-depleted. In SXRF, both the NNL and the accentuated stress marking are more clearly indicated by Zn-rich lines in dentine than they are in enamel (Figure 5).

### 3.2. Zinc Distribution in Modern Human and Great Ape Permanent Teeth

**Figure 6:**
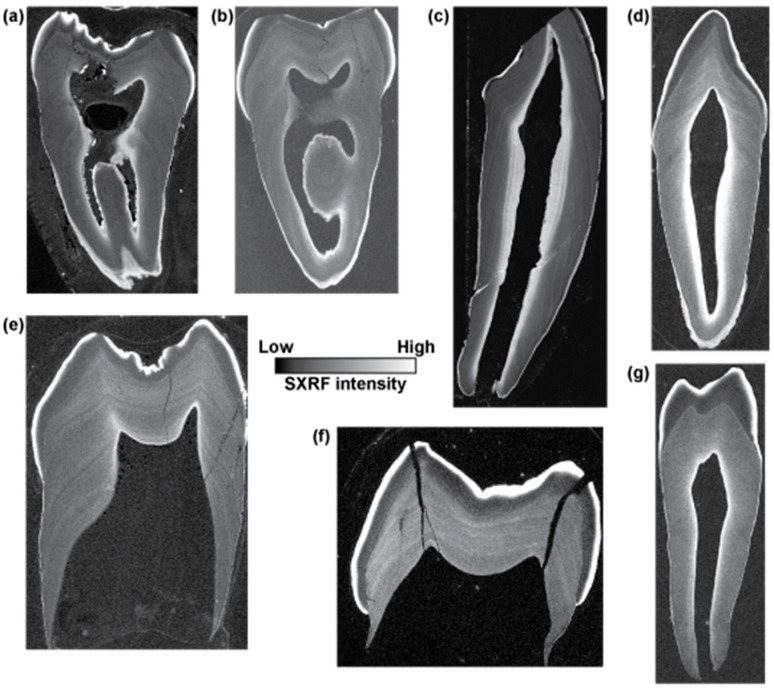
Zinc SXRF intensity maps of permanent modern great ape and human teeth: (**a**) *Pan* LM1, (**b**) *Pan* LM3, (**c**) *Pongo* female canine, (**d**) modern human upper canine (**e**) *Gorilla* LM2, (**f**) *Gorilla* unerupted LM3 from the same individual as (**e**,**g**) modern human LM3. In all teeth, including the unerupted LM3, surface enamel is Zn-rich. In all cases, where it has formed, secondary dentine and cementum are Zn-rich. The Zn distribution in dentine in each tooth broadly follows the incremental formation pattern. Images are not to scale.

Figure 6 shows SXRF maps of the Zn distribution in seven permanent teeth of varying tooth types. Two *Pan* molars from different individuals (LLM1 and LLM3), a permanent female *Pongo* canine, two *Gorilla* molars from the same specimen (LRM2 and LRM3), a modern human M3 and permanent canine. In all teeth, the outer enamel is Zn-rich, the secondary dentine and the cementum layer when formed is also Zn-rich. While the Zn-rich layer in the enamel and in the secondary dentine appears amorphous at this resolution, the dentine in all teeth shows evidence of irregular alternating Zn-rich and Zn-depleted bands that broadly follow the incremental growth pattern of the tooth. One tooth, the *Gorilla* LRM3, was extracted from its crypt for histological preparation. It was hence unerupted during life and never exposed to the oral environment. The Zn-rich outer enamel layer is clearly present in this tooth, as are the irregular Zn-rich bands in the coronal dentine.

### 3.3. Zinc Distribution in Permanent Teeth at Higher Resolution

**Figure 7:**
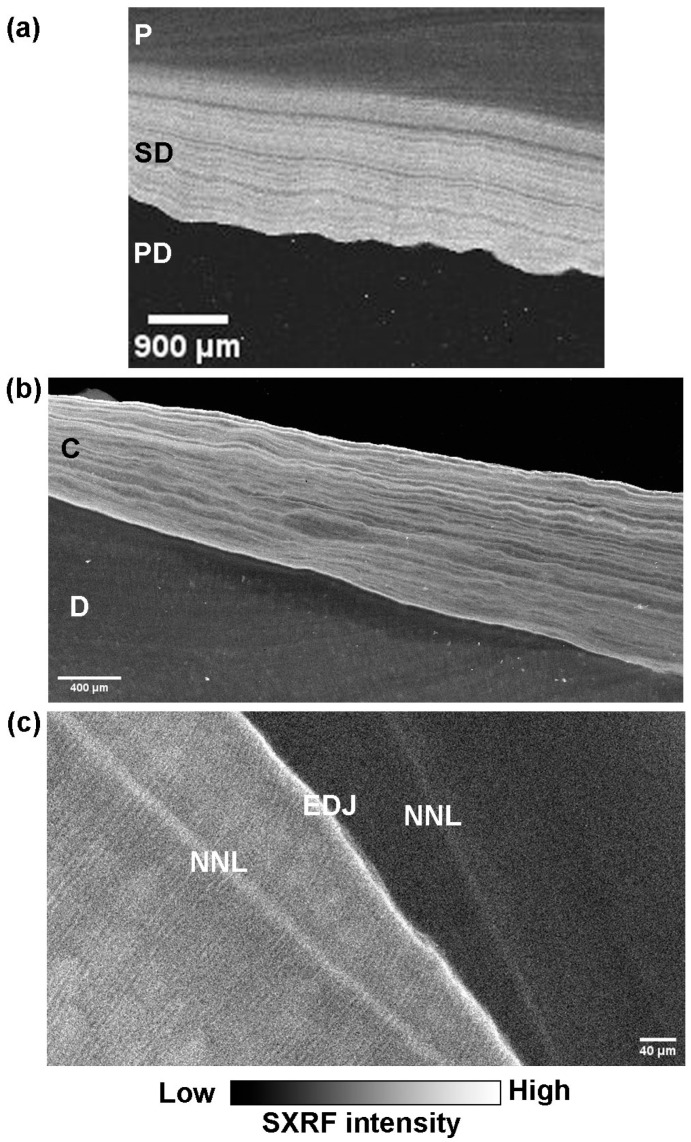
SXRF intensity maps of (**a**) secondary dentine in a modern *Pongo* female canine showing fluctuating Zn-rich and Zn-depleted zones that follow the incremental growth pattern of secondary dentine and contrast with the low levels of Zn in primary dentine. P = pulp, SD = secondary dentine, PD = primary dentine, (**b**) cellular cementum in a modern *Pan* permanent incisor root showing sharply formed Zn-rich annual growth increments, D = root primary dentine, C = cementum, (**c**) the Zn-rich NNLs in enamel and dentine converging towards the Zn-rich EDJ in a modern human upper dm2, and Image (**b**) is from Dean et al. [36] Figure 4d, Image (**c**) is from Dean et al. [34], Figure 4c.

**Figure 8:**
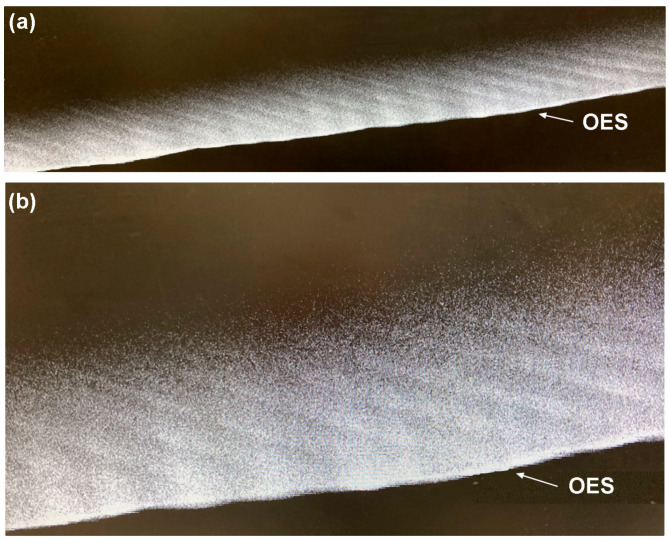
Zn SXRF intensity maps of a portion of the outer labial enamel surface (OES) of a modern human permanent canine tooth, scanned at 5 µm (**a**) and 3 µm (**b**). The long-period Retzius lines are Zn-rich. This then reduces in intensity immediately after Retzius line formation and then gradually builds up again towards the next Retzius line. Relative greyscale with black representing low Zn fluorescence intensity and white representing high intensity.

Figure 7 shows the distribution of Zn at the EDJ and NNL in a deciduous tooth and in secondary dentine and cementum at higher resolution. Both the Zn-rich NNLs in enamel and dentine converge towards the EDJ. In this modern human example (See also [34]), it can be clearly observed that the EDJ is also Zn-rich on the dentine side of the junction, and dentine tubules can be observed within it. Consistent with the lower resolution maps (Figure 6 and Figure 7), the dentine NNL shows greater SXRF intensity than the NNL in enamel. Zinc distribution in cellular cementum has previously been described [36,86] and by way of example can be observed here in *Pan* cellular cementum to be distributed as fine clearly separated Zn-rich incremental markings that are presumed to be annual. Zn distribution in secondary dentine also appears to be laid down in bands or increments but these seem to be generally less well defined than those in cementum and are of unknown periodicity. While the whole outer enamel region is relatively Zn-rich, the modern human example in Figure 8 shows that the greatest SXRF Zn intensity occurs along the regular long-period incremental markings (the striae of Retzius) but with just a thin outer Zn-intense layer corresponding to the thin layer of surface aprismatic enamel.

### 3.4. Zinc Distribution and Diagenesis in Permanent Fossil Teeth

**Figure 9:**
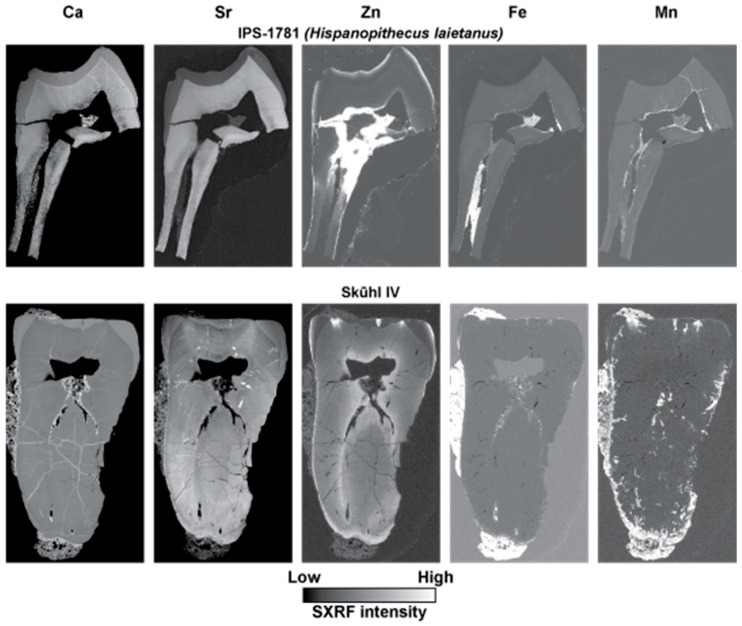
SXRF intensity maps for Ca, Sr, Zn, Fe and Mn shown for an UM1, IPS-1781 attributed to *Hispanopithecus laietanus* from the Late Miocene site of Can Llobateres, Spain, and for a LLM3, Skhūl IV, attributed to an early modern human from the site of Skhūl, Wadi el-Mughara, Mount Carmel, Israel. Outer enamel, secondary dentine and cementum remain Zn-rich but there is clear evidence of diagenetic change to Zn distribution where there has been demineralisation in the IPS-1781 tooth root and pulp chamber as well as Zn penetration of occlusal dentine tubules from overlying matrix in Skhūl IV. Images are not to scale.

**Figure 10:**
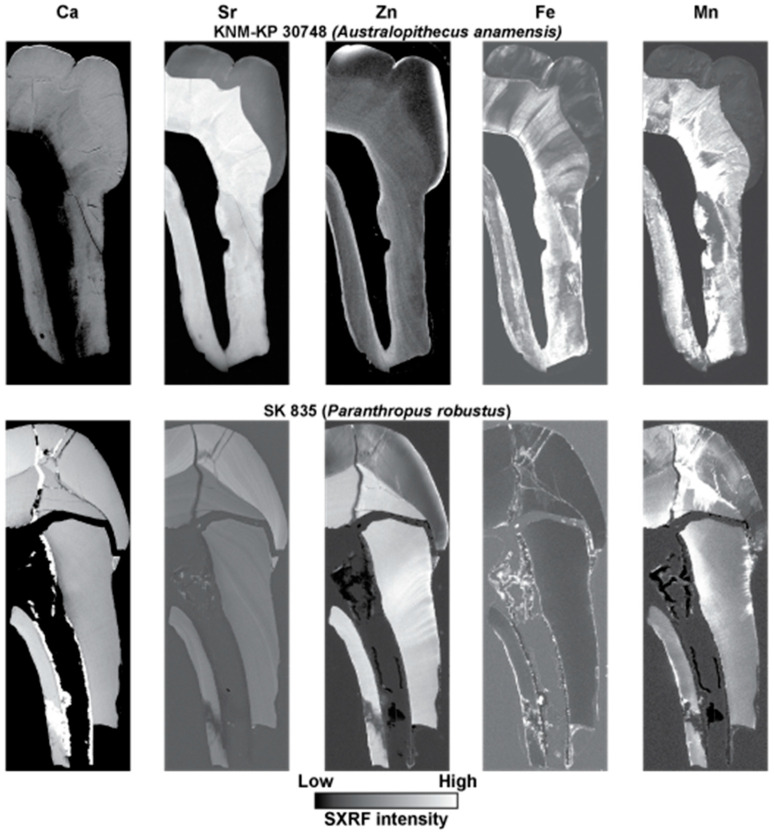
SXRF intensity maps for Ca, Sr, Zn, Fe and Mn shown for a mandibular premolar fragment, KNM-KP 30748 (**top row**), attributed to *Australopithecus anamensis* from Kanapoi, Kenya and for an UM3, SK 835 (**bottom row**), attributed to *Paranthropus robustus*, from Swartkrans, South Africa. The surface enamel, secondary dentine and cementum are Zn-rich in KNM-KP 30748, and the incremental growth pattern of dentine remains discernible in the Zn distribution map. In SK 835, surface enamel and cementum remain Zn-rich but the dentine shows strong evidence of Zn diagenesis with dentine tubules from the root surface to the pulp that is now Zn-rich. This has obscured the incremental pattern of dentine formation, but which remains clear in the Sr SXRF intensity map. Zn diagenesis does not follow the pattern of Ca, Sr, Fe or Mn diagenesis. Images are not to scale.

Figure 9 and Figure 10 show SXRF maps of four permanent fossil teeth. They show how the distribution of Ca, Sr, Zn, Fe and Mn compare with each other in each tooth.

In the *Hispanopithecus laietanus* UM1, IPS-1781, the SXRF Ca intensity map shows calcite within the matrix in the pulp chamber as the most intense but with dentine now more heavily calcified than enamel in this fossil. The Sr map also shows greatest intensity in the dentine and not the enamel. The preserved tooth root shows a loss of both Ca and Sr in a presumed demineralised region along its length. The Zn SXRF map shows the outer enamel to be Zn-rich, as in modern teeth, but with extensive SXRF Zn intensity extending into the presumed demineralised region of the root from the pulp chamber and into cracks and tubules radiating from the pulp on the root side. Matrix within the root canal and pulp chamber is Fe-rich but this has not spread through root dentine in the way Zn appears to have done. Similarly, Mn is distributed around the pulp chamber walls but has not generally penetrated the dentine tubules or matrix to the same degree as Fe. 

The LLM3 of Skhūl IV (Figure 9) still has matrix covering the enamel occlusal surface as well as the root surface and has also accumulated some matrix within the pulp chamber. This is calcite-rich and bright veins of calcite can be observed to have formed within the dentine crown and root. Zinc distributes in the cementum, especially apically where there is thicker cellular cementum, in the secondary dentine and in the outer enamel as it does in modern teeth, but where matrix covers the exposed worn enamel (just deep to the plane of section), there is clear evidence of Zn penetrating dentine tubules into the cuspal dentine. The enamel remains more heavily calcified than the dentine in this fossil tooth, as in life. The Sr map also shows that the peripheral coronal mantle dentine, but not the root dentine, contains less Sr than enamel. Strontium still retains something of its incremental distribution (unlike Zn in this fossil tooth) that follows the growth pattern of the tooth, but where there has been the most intense Sr uptake around the root apex, the incremental pattern is obliterated in places. Strontium follows the same diagenetic pattern as Zn, where it has diffused into the occlusal dentine via exposed dentine tubules just deep to the plane of the section. This pattern of diagenetic change resembles that of Mn occlusally but not of Fe. The matrix adherent to the tooth surface is both Fe-rich and Mn-rich, but not Sr or Zn-rich. Only Mn, and not Fe, has penetrated the cracks, fissures and tubules within the root dentine and cementum.

The SXRF intensity maps of KNM-KP 30748, the mandibular premolar fragment attributed to *Australopithecus anamensis* from Kanapoi, Kenya (Figure 10), show enamel and dentine that are nearly equally calcified but with no calcite visible in the Ca map. Sr intensity is much greater in dentine than enamel, indicating preferential Sr uptake through dentine tubules. Both Fe and Mn have also penetrated dentine from the pulp chamber and from the root surface. The Zn distribution, however, closely resembles that of a modern tooth with the greatest Zn SXRF intensity in cementum, the outer enamel and in the secondary dentine. Only Zn clearly still preserves the incremental formation pattern of the dentine that is almost entirely obliterated in the Ca, Sr, Fe and Mn maps.

The SXRF intensity maps of the UM3 attributed to *Paranthropus robustus*, from Swartkrans, South Africa, SK 835 (Figure 10) show calcite formed around the pulp chamber walls and along long-standing cracks in the dentine crown and into enamel. Enamel appears near-equally calcified as dentine, but the Sr map better resembles the situation in modern teeth with the enamel of greater SXRF Sr intensity and with the incremental structure in both enamel and dentine still visible. This suggests there has been minimal *post-mortem* Sr uptake in the dentine of this specimen. The Zn SXRF intensity map shows only a thin Zn-rich region of outer enamel and only a thin cementum layer in this M3 which had not yet completed root formation (or begun secondary dentine formation). However, there is clear evidence of Zn penetration along dentine tubules, most likely from the external root surface towards the pulp in this case; there is no evidence of any incremental pattern in Zn distribution that may now have been obliterated, or that alternatively, may never have existed. The pattern of Fe and Mn diagenesis is different. Fe SXRF intensity is greatest at the enamel and dentine surfaces and in the matrix within the pulp chamber and the cracks between the enamel and dentine fragments. However, Mn penetration has occurred through cuspal enamel more extensively and into the dentine tubules occlusally, as well as through root dentine tubules from both the pulpal and root surface.

### 3.5. Zinc Distribution and Diagenesis in Deciduous Fossil Teeth

**Figure 11:**
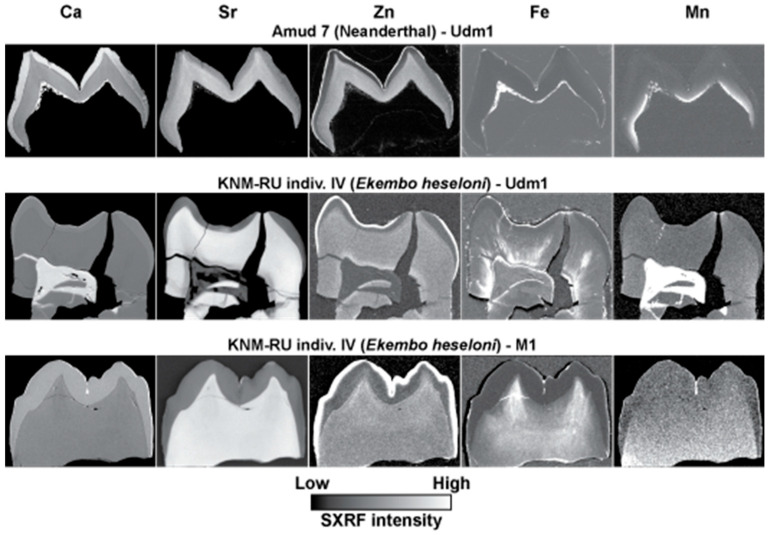
SXRF intensity maps for Ca, Sr, Zn, Fe and Mn shown for deciduous teeth, Amud 7, Udm1, attributed to a Neanderthal infant from the Amud cave site, Israel, and an LRdm2 and a LLM1, also belonging to KNM-RU individual IV and attributed to *Ekembo heseloni* from the site of Rusinga Island, Kenya. Surface enamel remains Zn-rich in all three teeth except in regions of the Amud 7 outer enamel that have been eroded *post-mortem*. The secondary dentine in KNM-RU individual IV LRdm2 remains Zn-rich but was not formed in the Udm1 of Amud 7 and is not preserved in the *Ekembo* LLM1. The pattern of diagenetic change in Ca, Sr, Fe and Mn distribution is different in each tooth but is not associated with any obvious diagenetic change in Zn distribution. Images are not to scale.

The SXRF intensity maps of three fossil deciduous teeth (Figure 11) show how the distribution of Ca, Sr, Zn, Fe and Mn compare with each other in each tooth. The Udm1 germ of Amud 7 was unerupted at the time of death and so was never exposed to the oral environment. The enamel surface of this tooth is also eroded in places *post-mortem* and was presumably exposed to the burial environment in this state. The Ca SXRF intensity map shows that enamel is more heavily calcified than dentine, as in modern teeth, but also shows calcite lining the pulp walls within the matrix here. The Sr map shows the greatest SXRF intensity in the prenatal cuspal enamel and preserves the incremental structure of the dentine as it does in modern deciduous teeth. Zinc is also distributed as it is in modern deciduous teeth with the greatest SXRF intensity in the outer enamel and in the prenatal dentine. No Zn from the external environment appears to have entered the surface enamel where it is eroded, nor has it penetrated dentine from the pulpal aspect. Secondary dentine had not formed in this tooth and so there is no Zn-rich layer around the pulp chamber. The matrix in the pulp cavity is both Fe-rich and Mn-rich, but Fe is confined to a thin surface layer of enamel and pulpal dentine, whereas Mn appears to be absent over the enamel crown but has penetrated dentine tubules from the pulpal aspect.

The LRdm2 of KNM-RU individual IV attributed to *Ekembo heseloni* from Rusinga Island, Kenya, shows Ca SXRF intensity to be equal in both enamel and dentine but with calcite formed in the matrix within the pulp chamber. Strontium uptake in dentine has far exceeded that in enamel, but Zn distribution still resembles that in modern deciduous teeth with a Zn-rich surface enamel layer and Zn-rich secondary dentine layer surrounding the pulp chamber. In this tooth, Fe forms a thin layer over the enamel, lines the pulp chamber, and has near-completely penetrated the dentine tubules. Manganese intensity is greatest in the matrix within the pulp chamber and over the root dentine but has not spread like Fe along the dentine tubules.

The LLM1 of KNM-RU individual IV, also attributed to *Ekembo heseloni,* shows Ca SXRF intensity to be still greater in enamel than dentine, as in modern teeth, which, therefore, differs in this respect from the LRdm2 of the same individual. However, Sr SXRF intensity is much greater in dentine than enamel, indicating greater uptake. Zinc distribution at the surface enamel resembles that of modern teeth; there is a suggestion of some Zn enrichment in the dentine horns and along the EDJ. No pulp chamber is preserved in this section, although dentine tubules within the cusps are Fe-rich, suggesting that Fe has penetrated up from the pulp chamber, as there is no evidence of Fe enrichment in the enamel of this unworn tooth. This differs from the distribution of Mn in this tooth that shows no evidence of penetrating either enamel or cuspal dentine, although the unpreserved pulp chamber may well, like the other tooth from this individual, have contained a Mn-rich matrix.

**Figure 12:**
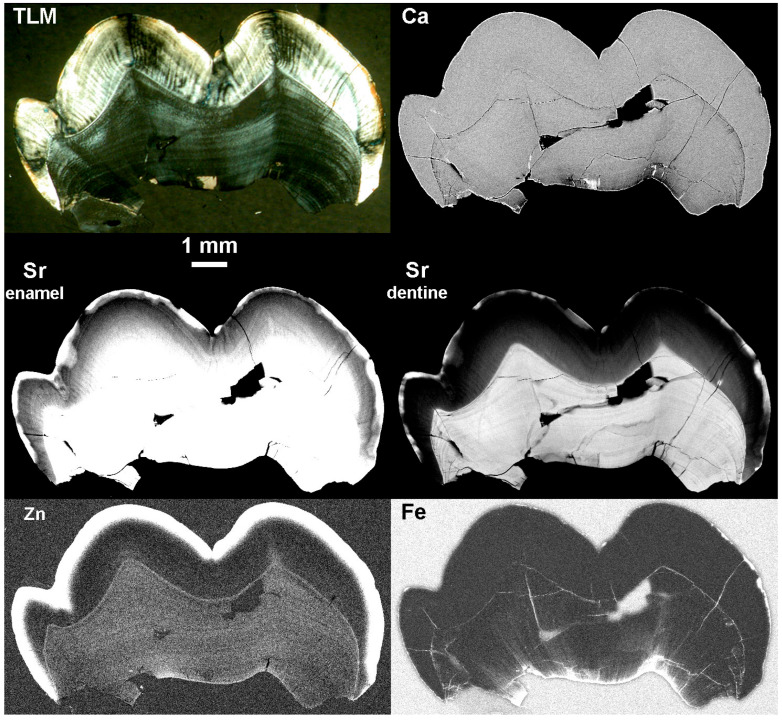
TLM and SXRF intensity maps for Ca, Sr, Zn, and Fe of the URM1 attributed to *Ekembo nyanzae* from Rusinga Island, Kenya, KNM-RU 1721. While the Ca signal in the enamel is now indiscernible from that of dentine, Zn distribution and enrichment of the outer enamel surface and at the EDJ still resembles that in modern teeth. Sr intensity is far greater in dentine than in enamel and has diffused into surface enamel and enamel closest to the dentine. Despite this, a trace of the incremental tooth formation pattern is still discernible in the Sr maps. Relative greyscale with black representing low elemental fluorescence intensity, and white representing high intensity.

In their original description of the histology of several teeth from the Kisingiri site on Rusinga Island, Kenya, all now attributed to *Ekembo* sp. [87], Beynon et al. [66] described what appeared to be regular accentuated markings preserved in the coronal dentine. These were approximately 90–100 days apart in the larger teeth, now attributed to *E. nyanzae,* and approximately 50–55 days apart in the smaller teeth, now attributed to *E. heseloni*. No obvious physiological, seasonal or cyclical fruiting/feeding cause for these accentuations seemed likely, although they resemble stress events in some modern teeth. In the SXRF maps for the URM1, KNM-RU 1721, attributed to *E. nyanzae*, the Ca map (Figure 12) shows little evidence of these markings, but both the enamel and dentine are now heavily calcified to a near identical degree and any previously visible hypocalcified markings would likely be obliterated. The dentine map shows much greater Sr intensity than enamel through diagenetic uptake *post-mortem* and with Sr diffusion also occurring into surface enamel and into dentine from the pulpal surface via dentine tubules. Despite this, something of the incremental formation pattern and of the cyclical stress markings in both the enamel and dentine of this specimen can be discerned in the Sr maps. The distribution of Zn, however, still resembles that of modern teeth with Zn enrichment of the surface enamel, of the EDJ and with a Zn incremental formation pattern still visible in the coronal dentine. Again, something of the cyclical stress markings remains visible in the Zn map. No Mn map was available for this tooth section, but the Fe map shows surface diffusion from the pulpal dentine surface, from surface enamel into the deeper tissues and along long-standing cracks and fissures. 

### 3.6. Zinc Distribution at the Neonatal Line in Fossil Teeth

Three fossil teeth that preserve the NNL were imaged with transmitted light microscopy. The LLM1 of KNM-RU individual IV shows a clear NNL in both enamel and dentine, exactly where they would be positioned in a modern human or great ape M1 protoconid. When mapped with SXRF for Zn at high-resolution, the enamel NNL is clearly visible but with the suggestion that prenatal enamel is Zn-depleted relative to postnatal enamel in a way that resembles some modern human teeth (Figure 3). However, the dentine NNL is not visible as a Zn-rich line but rather is demarcated by a prenatal Zn-depleted region and a Zn-rich postnatal cuspal dentine region. This differs from the usual Zn distribution in modern teeth but resembles some of the modern great ape deciduous teeth imaged in Figure 3. The EDJ in this LRdm2 specimen is clearly demarcated as Zn-rich, as it is in modern deciduous teeth.

The LRdm2 of KNM-RU individual IV shows clear NNLs in both enamel and dentine when imaged with transmitted light microscopy. The SXRF map for Zn intensity shows an enamel NNL, with the suggestion again that prenatal enamel is Zn-depleted relative to postnatal enamel. The prenatal dentine is more clearly Zn-depleted relative to the post-natal dentine and so resembles the situation observed in the LM1 of the same individual and in some modern great ape teeth (Figure 3). The EDJ in this LRdm2 is again clearly Zn-rich, as it is in modern deciduous teeth (Figure 4).

**Figure 13:**
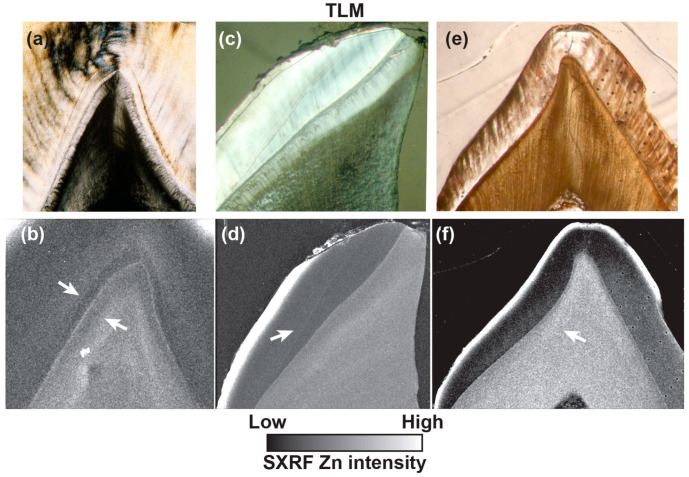
Pairs of transmitted light micrograph images and SXRF Zn intensity maps of the cuspal region of three fossil deciduous teeth containing the neonatal line (NNL; white arrows) and cuspal EDJ. In TLM (**a**) and SXRF (**b**), the cusp of the LLM1 of KNM-RU individual IV (attributed to *Ekembo heseloni* from Rusinga Island, Kenya) shows a clear NNL in both enamel and dentine. These and the EDJ remain Zn-rich even after ~17 million years. Similarly, (**c**,**d**) show matching images of the LRdm2 of KNM-RU individual IV attributed to *Ekembo heseloni*. Again, the NNLs and EDJ remain Zn-rich. As in the modern human deciduous teeth (see Figure 4 and Figure 5), the impression from the Zn SXRF intensity maps (**b**,**d**) is that prenatal enamel is Zn-depleted relative to postnatal enamel. However, prenatal dentine in both the LRdm2 and LLM1 (**b**,**d**) resemble the modern great ape specimens described here (Figure 3) and also appear Zn-depleted and not Zn-rich as in the modern humans described (Figure 3 and Figure 4). In Amud 7 Udm1, the NNL is visible in enamel in TLM (**e**). The SXRF intensity image of Amud 7 Udm1 (**f**) however, does not preserve a Zn-rich NNL in either enamel or dentine but does suggest Zn-rich prenatal dentine as in the modern human deciduous teeth (Figure 3 and Figure 4). Images are not to scale.

The cusps of the Amud 7 Udm1 both show a clear NNL in enamel when imaged with transmitted light microscopy. Just one cusp is imaged here (Figure 13). While the outer enamel is clearly Zn-rich in this specimen, the higher resolution Zn SXRF map shows no evidence of a Zn-rich enamel NNL nor of Zn-enrichment at the EDJ in this specimen. There is, however, the suggestion of a Zn-rich zone of prenatal dentine in the cusp tips, as is usually observed in modern human teeth (Figure 3).

### 3.7. Quantification of Zinc Distribution in the Outer Enamel

**Figure 14:**
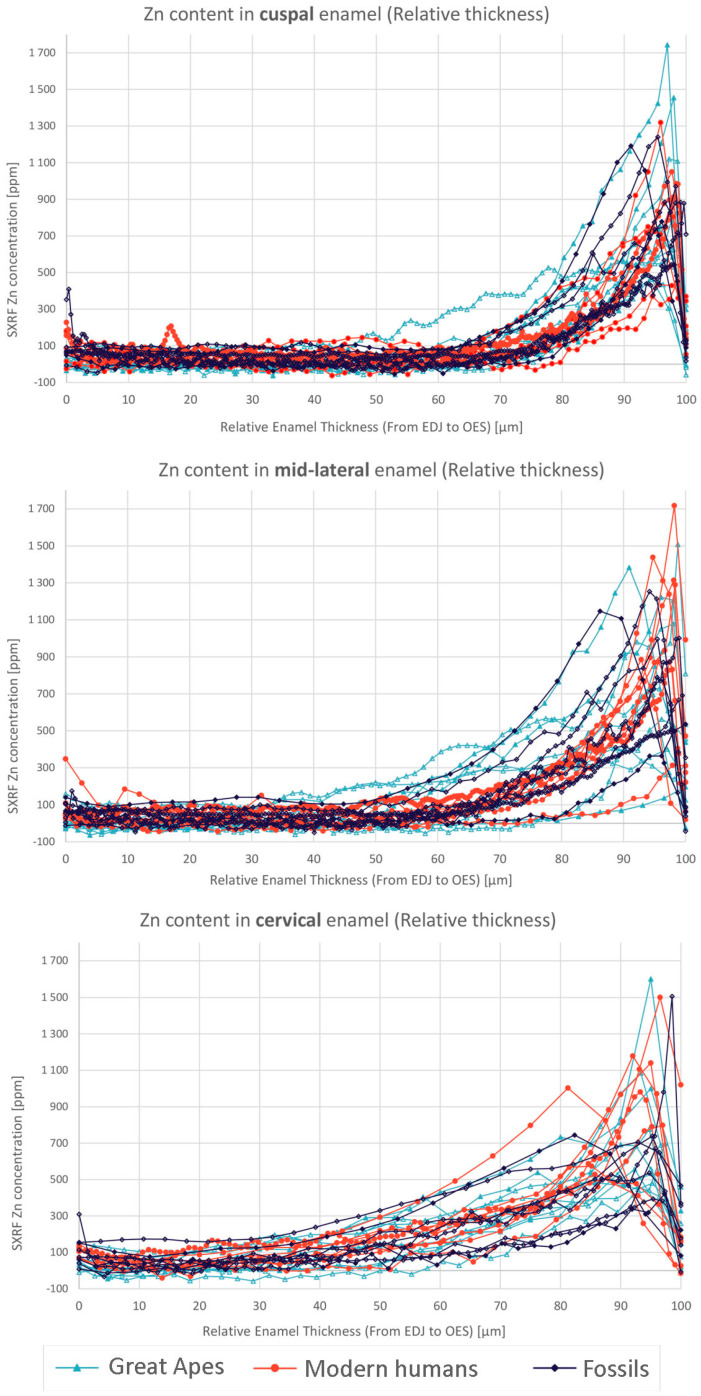
Enamel thickness varies greatly, so the rise in Zn concentration towards the outer enamel surface (OES) is plotted here against relative thickness of enamel. This was calculated as the linear percentage distance from the EDJ to the OES at three positions. These three transects through enamel are cuspal, mid-lateral and cervical. Each tooth used and each transect are shown in Appendix SA. The modern *Homo sapiens* sample (*n* = 8) are plotted in red, the modern great ape sample (n = 10) in light blue and the fossil hominoid sample (*n* = 8) in black. In cuspal enamel, the rise in Zn concentration begins 70–80% through the enamel but occurs earlier (40–50%) in cervical enamel. Zn concentration in inner enamel is always below ~200 ppm. Maximum values attained in outer enamel rise to between 434–1743 ppm (cuspal), 197–1717 ppm (mid-lateral) and 393–1602 ppm (cervically).

**Figure 15:**
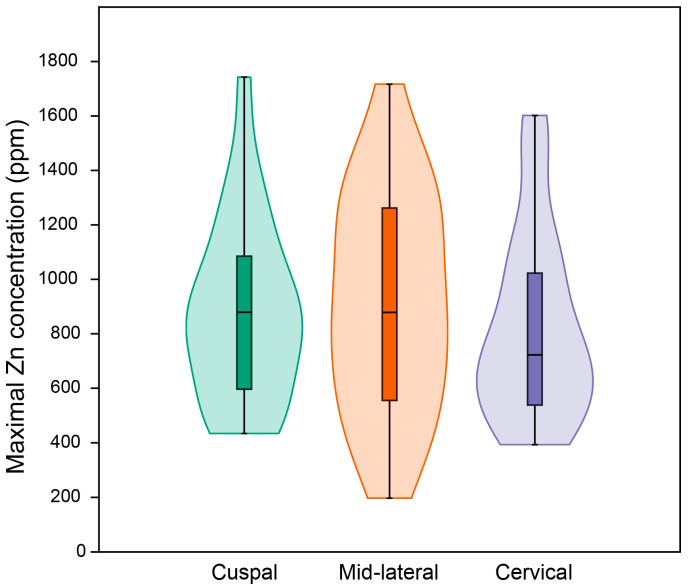
Violin and box (median) plots of the maximum values (peak) of Zn concentration within the Zn-enriched enamel band. All plots are for the whole sample of *n* = 26 modern and fossil, deciduous and permanent teeth. (See Appendix SA for raw data and Figures S1–S6 for further analysis of the data).

**Figure 16:**
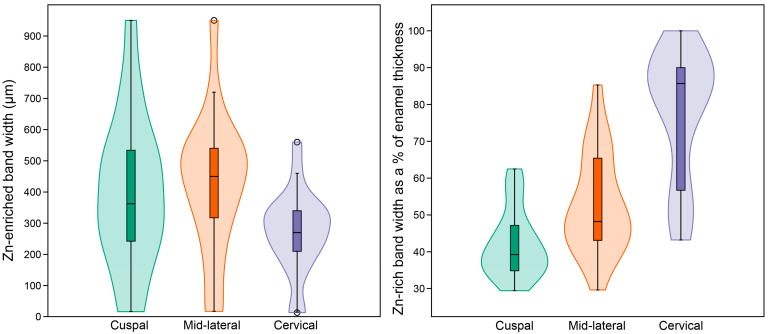
Violin and box (median) plots, split by enamel region (cuspal, mid-lateral and cervical) for the width of the enriched Zn band and the percentage proportion of the Zn-rich surface band relative to the regional enamel thickness. All plots are for the whole sample of *n* = 26 modern and fossil, deciduous and permanent teeth. (See Appendix SA for raw data and Figures S1–S6 for further analysis of the data).

Figure 14 demonstrates that in all teeth studied here, the Zn concentration is less than ~200 ppm in inner enamel and then rises to a maximum value towards the enamel surface. Appendix SA presents data for all regions of all individual teeth studied here. The rise in Zn concentration begins at about 70–80% of the total linear enamel thickness in cuspal enamel, or 40–50% in cervical enamel, and increases exponentially to maximum values, that close to the outer enamel surface, reach 434–1743 ppm (cuspal), 197–1717 ppm (mid-lateral) and 393–1602 ppm (cervically) (Figure 15, see Table on page 4 of Appendix SA). Maximum Zn concentration values in surface enamel within an individual tooth are not always equivalent between regions and are occasionally twice that in one region compared to another region. This broad range of maximum regional Zn concentrations in outer enamel overlaps across permanent and deciduous teeth. It also overlaps between fossil and modern teeth and cannot then be used to distinguish between them (See Figures S1–S6). Even between deciduous teeth from the same individual (specimens S6, S7 and S8, Appendix SA), there are low values in an Udm1 (451, 313, 574 ppm, for cuspal, mid-lateral and cervical, respectively), intermediate values in an Udm2 (1050, 1289, 1179 ppm) and even higher values in a Ldc (724, 1717, 1501 ppm). Between teeth of all tooth types, the band of Zn-enrichment at the enamel surface varies in width narrowing towards the cervix (Figure 16). However, in an individual tooth, the width of the surface band of high Zn concentration varies and may be relatively broad or narrow (see Appendix SA). Across all teeth in this sample (both deciduous and permanent) the proportion of the Zn-enriched band relative to the regional linear enamel thickness rises markedly from cuspal to mid-lateral to cervical enamel (Figure 16; also see Figures S1–S6).

To explore further the characterisation of this Zn-enriched zone, statistical tests were performed, for which details are provided in Appendix SB. Principal component analyses reveal that, for all teeth pooled together and the three anatomical regions (cuspal, midlateral and cervical) combined, it is enamel thickness (µm), the width of the Zn-enriched band of enamel (µm), and the distance between the take-off of the Zn gradient to the peak concentration in Zn (µm) that contribute the most to the first principal component which separates modern humans from the *Gorilla* and *Pan* group (see PCA results in Figures 19–23 in Appendix SB). Fossil specimens are rather isolated from these two main clusters. PC1 always accounts for 60–65% of the total variance, while PC2 represents 20–25% of the variance, and is mainly explained by the maximal concentration in Zinc. The distance between the outer enamel surface and the peak in Zn concentration seems to be more weakly involved in the total variance (Figures 19–23 in Appendix SB).

Correlation tests further demonstrate that, when all tooth types and anatomical regions are pooled together, the strongest significant (positive) correlations are found first, between the width of the Zn-enriched band and the distance between the take-off of the gradient to the Zn concentration peak, and second, between the total enamel thickness and the width of the Zn-enriched band, as well as between the distance from the take-off of the gradient to the peak in Zn concentration. Much more weakly correlated is the distance between the OES and the peak in Zn concentration (Figures 1–9 in Appendix SB). The correlations between the distance between the Zn peak and the OES, and the distance between the take-off of the Zn gradient to the Zn peak or the total width of the Zn gradient, are more moderately positively correlated, when significant at all (Figures 1–9 in Appendix SB).

When the three anatomical regions are considered together, the correlation coefficient between enamel thickness and the width of the Zn-enriched band is significantly higher than the one between enamel thickness and the distance from the OES to the Zn concentration peak. This means that the position of the Zn concentration peak varies more freely within the Zn-rich band than the width of the band does in relation to the total enamel thickness.

Overall, permanent teeth show a significantly lower maximal Zn concentration than deciduous teeth (Figure 24 and Wilcoxon test: *p* ≈ 0.01, in Appendix SB). Yet, when permanent and deciduous teeth are pooled together, there is no significant difference in the maximum Zn concentration between cuspal, midlateral and cervical regions (Figure 25 and ANOVA test: *p* ≈ 0.53, in Appendix SB). There is no significant interaction between tooth type (deciduous vs. permanent) and enamel region (cuspal, midlateral or cervical) (*p* ≫ 0.05, permutations tests on pp. 28–29 in Appendix SB). To summarise, permanent teeth have consistently lower Zn concentrations than deciduous teeth, and all regions (cuspal, midlateral and cervical) in permanent teeth are reduced to the same degree.

When all tooth types (posterior, anterior, permanent and deciduous), and all three anatomical regions are combined, the maximum peak in Zn concentration does not correlate with any of the measured enamel distances (i.e., total enamel thickness, width of the Zn-enriched enamel band, distance from the OES to the Zn concentration peak, or distance between the take-off point of the gradient to the Zn concentration peak) (Figures 1–9 in Appendix SB).

## 4. Discussion

A consistent finding of this study is that in both modern and fossil hominoid teeth, the outer enamel, the cementum and the secondary dentine surrounding the pulp chamber are all Zn-rich. This confirms the findings of several previous studies on modern human teeth [7,17,25,27,28,38,39,88]. But Zn may be present in dental tissues for different reasons. Previously, it has been suggested that where mineralising tissue formation is slow, as in peritubular dentine, secondary dentine and cementum, a greater amount of Zn may be taken up from direct contact with tissue fluid over time [28,35,36,37]. Equally likely in cementum, however, is that as with osteoblasts in bone formation, [10,11,12], Zn is associated with the processes involved with periodic cycles of cementoblast differentiation, proliferation and matrix secretion. At the future EDJ, odontoblast differentiation precedes ameloblast differentiation and Zn may be a residual component of growth and transcription factors originally involved with the regulation of odontoblast differentiation at the EDJ [13,18]. Similarly, at the enamel surface, it has been suggested that Zn may be a residual degradation product of specific serine protease enzymes involved in the enamel maturation process or perhaps be involved in controlling KLK-4 activity during the later phase of enamel maturation [11,16,17,19].

### 4.1. Zinc Incorporation into Enamel and Dentine

The evidence presented in this study for dentine, and possibly for secondary den-tine, but not convincingly for outer surface enamel, suggests that there are fluctuations in Zn concentration that broadly follow the incremental formation pattern of the tooth, and so would seem likely to reflect changes to levels of ingested Zn. Zinc enrichment in prenatal dentine and at the NNL also seem to reflect physiological shifts in blood plasma Zn levels [1,23]. However, previous experimental work where rats were fed diets containing high levels of Zn failed to influence Zn levels in molar enamel that remained as low as 190 ppm [46]. It also seems clear that teeth from individuals from the same and different geographical areas have marked differences in Zn concentration [7] that are not obviously explained by levels of Zn ingestion. It follows that the fluctuations in Zn intensity (concentration) in prenatal dentine, dentine and secondary dentine observed in this study appear to run counter to previous findings on enamel that have concluded that variation in Zn concentration does not seem to be directly related to dietary intake [46]. Future studies that are able to track both shifts in Zn concentration in dentine alongside other isotopic evidence for seasonality [89,90,91] and dietary shifts [32] throughout tooth formation in hominoids may shed light on whether or not, for example, seasonal and/or dietary changes underlie the kind of incremental Zn fluctuations observed in this study.

The evidence from the results of this study that this holds true for enamel and cementum is less clear than for dentine, but in horse enamel [89], seasonal fluctuations in several trace elements, including Zn/Ca detected by micro-X-ray Fluorescence scanning, reliably track shifts in stable oxygen isotope ratios, that in turn directly reflect temperature and precipitation. de Winter et al. [89] have proposed that the source of these raised trace element levels in horse teeth is an increase in the levels of dust that increase through the summer months. Both dust and soil ingestion have previously been suggested as a source of trace elements in teeth [92,93,94,95]. Moreover, in mammoth teeth, where the enamel plates take many years to form, there is clear evidence of a seasonal fluctuation in Zn concentration, especially in inner enamel towards the cervix of the long enamel plates [90].

In the case of enamel, given the findings for both unerupted extant and fossil tooth germs reported here, the Zn-rich outer layer seems now to be clearly established prior to eruption into the oral environment, even though subsequently there would potentially be some additional post-eruptive acquisition of Zn that may or may not alter the isotopic composition at the surface [61]. The higher resolution images of Zn in modern human outer enamel (Figure 8) show Zn-rich long-period striae of Retzius. If Zn in the outer enamel is indeed residual and retained from the enzymatic degradation process that occurs during the enamel maturation phase, then the most porous or least dense structures that retain the most organic material [96,97,98], to which Zn also readily binds, appear to retain the most Zn. However, given that this could also be interpreted as an incremental fluctuation in Zn concentration established during the secretory process, the role of the secretory ameloblast may need to be reconsidered [47] rather than simply assuming that the Zn-rich outer enamel layer is the result of an entirely maturational enzymatic degradation process. As with fluoride incorporation into enamel [37,99], as well as Mg and carbonate content [100], the distribution of Zn in enamel may reflect the composition of mineral formed both during the earlier secretory phase and during the late maturational phase via paracellular ion diffusion between smooth-ended maturational ameloblasts [15]. The assumption, however, that any change in ion concentration to higher or lower levels along an isochronous-accentuated line passing from the EDJ to the OES is always indicative of an enamel maturational effect, independent of the secretory phase ameloblasts, may not hold true for all elements [90], given the complex resorptive and ion transport functions of secretory ameloblasts [47].

Gradients of Na^+^, Mg^2+^, CO_3_^2−^ and Sr^2+^ concentration exist within enamel and are each the highest in concentration towards the EDJ and lowest in concentration towards the enamel surface [17,37,88,100,101,102]. The reverse is true of Zn^2+^, which is the highest in concentration towards the surface enamel [17,29]. As such, Zn^2+^ and Sr^2+^ levels have been observed to have a reciprocal relationship to one another in outer enamel. Strontium has a greater ionic radius (1.12 Å) than Ca (0.99 Å) and expands the hydroxyapatite crystal lattice when exchanged with Ca. However, when there is co-substitution of Sr^2+^ and CO_3_^2−^, especially in the presence of fluoride ions, this difference is offset, thus improving the stability of the lattice [4,48,103,104]. This scenario might be favoured to the exclusion of Zn in inner enamel where the concentration of both Sr^2+^ and CO_3_^2−^ ions is greatest. When both Zn and Sr are co-substituted, then the smaller Zn ions (ionic radius 0.074 Å) have a destabilising influence on both the hydroxyapatite lattice composition and its degree of distortion [105]. It follows that, in outer enamel, where Sr concentration is relatively low, Zn substitution for Ca may be favoured here to the exclusion of Sr and that it may be the role of the secretory ameloblast to control the ionic composition of the enamel fluid [47]. In this regard, it has been reported that Zn concentration in the ameloblast nucleus increases during the secretory phase, reaching a maximum during the early maturation phase [11,106]. On the other hand, in outer enamel, Zn may predominantly bind with PO_4_^3−^ sites at the crystal surface, where it forms a Hopeite-like phase, in which case there would be less conflict with other trace elements that substitute within the crystal lattice.

### 4.2. Zinc at the NNL and as a Marker of Stress Events

Zinc levels before and after birth seem to be reflected in the enamel and dentine of modern human deciduous teeth formed at this time [34]. Elevated Zn levels perinatally may be incorporated into forming hydroxyapatite crystals and/or bind with components of the dentine protein matrix at a time when Zn levels are exceptionally high in the neonate. The apparent Zn-depleted enamel in some modern teeth, as imaged in Zn SXRF intensity maps, may in fact be illusory, as the quantification of the concentration of Zn in enamel shows no difference between pre- and postnatal enamel [34]. Zinc levels may, however, be lower than in prenatal dentine if there is conflict in prenatal enamel between Zn incorporation and the presence of high Sr and carbonate levels [105]. Zinc in dentine not only binds, in one way or another, to hydroxyapatite but also to the non-collagenous dentine protein matrix [35]; this may also account for the greater difference in Zn concentrations apparent between pre- and postnatal dentine and enamel in modern human teeth reported in Dean et al. [34]. In both enamel and dentine, the NNL usually shows a peak in Zn concentration that likely corresponds with the release of stored Zn from the liver at birth and, in breastfed infants, with high levels of Zn in colostrum [34]. 

That Zn should be preserved at the NNL in fossil enamel and dentine is remarkable, and especially so given that the teeth attributed to *Ekembo* date to ~17 Ma (Figure 13). This attests to the tenacity of Zn in both enamel and dentine over such a long period of time when other trace elements in the same teeth show clear evidence of diagenetic change. Fossil teeth are likely to be almost entirely anorganic and it may be that the Zn preserved in fossil dentine is partially residual following the loss of the non-collagenous protein matrix. Given that the NNL in both enamel and dentine and the EDJ are deeply positioned within the tooth, it seems that Zn here is the most likely to preserve a true biogenic isotopic composition.

Neither the modern great ape deciduous teeth in this study nor the fossil teeth attributed to *Ekembo* show any evidence of prenatal Zn-enrichment in either enamel or dentine. This difference between the modern human deciduous teeth and the great ape and *Ekembo* teeth sampled here may relate to a longer gestation period in modern humans and to a unique pattern of growth. In the same way that a greater amount of fat is accumulated in the last weeks of human gestation than in non-human primates [107], Zn also appears to accumulate at the same time prenatally, as judged by the greater amount incorporated into dentine, and both may relate to the greater energetic demands of brain growth [107,108]. Chimpanzees have a shorter gestation period (around 237 days versus roughly 266 days in humans) and Zn transfer to the infant, again as evidenced by the greater concentration present in postnatally formed dentine, may now occur after birth via breastmilk, although no data are available to support any potential difference in neonatal physiology or breastmilk composition. Notably, in the Amud 7 Neanderthal Udm1, no Zn-rich NNL was visible at all in the SXRF intensity maps although, as in the modern human teeth sampled here, some evidence of Zn-enrichment prenatally in the dentine is apparent (Figure 13).

Birth is a stressful event that involves major physiological changes, but other stressful events may also be associated with raised Zn levels that become incorporated into tooth tissues. Figure 5 illustrates a Zn marking in dentine associated with a known stress event that occurred shortly after birth [85]. Exactly whether the specific stress was a general anaesthetic, a post-operative raised temperature or a response to surgery and/or the subsequent wound healing process is unknown, but both after the NNL at birth and the stress event there is a zone of Zn depletion in dentine, suggesting a similar response to both.

### 4.3. Quantification of Zinc Distribution in the Outer Enamel

The results presented here for the pattern of Zn distribution in enamel confirm those from previous studies [7,17,28,29,30,31,32,34,75]. In their sample of modern and archaeological human teeth, Brudevold et al. [7] reported concentrations in outer enamel that ranged between 430 ppm (from Greenland), 900 ppm (from Big Lake), 1700 ppm (from Schenectady) to 2100 ppm (from Augusta). However, the maximum value for the latter sample was the only one that exceeded the range of values reported in this study, which otherwise overlap with those of the present comparative sample of modern and fossil hominoid teeth (197–1743 ppm). Humphrey et al. [30] found elevated Zn/Ca ratios in the subsurface enamel of fully erupted archaeological M1, M2 and M3 from the same individual to a depth of 300–400 µm and noted that this was greater than the depth to which elevated Pb/Ca levels were observed (50–70 µm). Bourgon et al. [32], in a study of modern and fossil teeth, reported a distribution pattern of Zn concentration matching the one reported here, with maximum values of between ~200 and 700 ppm but with levels in inner enamel generally below 100 ppm and none rising to 200 ppm, as in this study. In the present study, the outer 20–30% of cuspal enamel was Zn-rich, and as much as 70% in the cervical region of some teeth (Figure 15). Also in their sample, the outer 20–30% enamel is relatively Zn-rich above the baseline level of ~100 ppm. However, the sample only contained teeth from one primate species (*Macaca* sp.), the majority being teeth from thin-enamelled omnivores, herbivores and carnivores (See SI Figure S30 on page 38 in [32]). The maximum values for Zn concentration reported in their study fall towards the lower end of the range for hominoids reported here. While in this study there was no relationship between enamel thickness and Zn concentration level (see Appendix SB), the effects of shorter linear enamel formation times and shorter enamel maturation times in the thin-enamelled non-primate enamel may warrant further consideration. In the same way, with regard to the primate teeth studied here, it remains possible that longer enamel maturation times in permanent teeth are associated with greater amounts of Zn removal following protein degradation than in deciduous teeth, resulting in lower maximal Zn levels in outer enamel. The very similar values and distribution pattern of Zn concentration levels in unerupted tooth germs compared with erupted teeth in this study support the conclusion that Zn levels in outer enamel are established prior to exposure to the oral environment and do not obviously become further enriched even through diagenetic change *post-mortem* in the fossil teeth reported here.

### 4.4. Diagenetic Changes in Fossil Tooth Tissues

In each of the fossil teeth studied here, there was evidence that the distribution of Zn in dental tissues resembled that in modern teeth, either those of modern humans or great apes. In many fossil teeth, Ca levels are higher in dentine than in modern teeth, and in geologically older fossils, these often match levels in fossil enamel. Loss of Ca and Sr in the root dentine of the *Hispanopithecus laietanus* UM1 specimen, IPS-1781, suggests there has been demineralisation and here there is the strongest evidence among any of the fossil teeth studied of Zn diagenesis and re-incorporation into dentine. Strontium uptake and overprinting in dentine and cementum, which in many older fossils even comes to exceed Sr levels in enamel, appears not to have altered the distribution of Zn in secondary dentine or cementum when present. In one tooth, SK 835, attributed to *Paranthropus robustus* from Swartkrans, South Africa, where there is evidence of Zn diagenesis in dentine penetrating tubules from the root surface to the pulp chamber, there is the least diagenesis apparent in Sr distribution than in other fossil teeth, again suggesting that there is no direct association between changes in Zn and Sr diagenesis. Neither does there seem to be any consistent pattern in the way Fe and Mn become incorporated into fossil tooth tissues, and there is no evidence of Zn incorporation *post-mortem* being associated with either. Apart from the evidence of demineralisation, it would seem that any diagenetic changes to Zn are not consistently associated with diagenetic changes to Ca, Sr, Fe or Mn.

Bourgon et al. [32] have previously noted that Zn in the outer enamel of fossils distributes in the way it does in modern teeth and appears also to preserve its original isotopic composition. Anczkiewicz et al. [90] have also shown this to be true for ^87^Sr/^86^Sr and for a number of other trace elements including Zn in more recent Upper Paleolithic mammoth enamel, all of which fluctuate seasonally. Even though Weber et al. [61] have demonstrated that the outer 200–300 µm of enamel is susceptible to changes in isotopic composition, the deeper enamel may not be [90]. In this respect, the Zn^2+^ ion in enamel may resemble the F^−^ ion, where changes to fluoride levels in the water during enamel formation (from 0.2–6 ppm) have a considerable effect on surface enamel concentrations in fully formed teeth that increase from 1500 to 5000 ppm, but do not change the overall pattern of F_2_ distribution through enamel [109]. However, after enamel completion, even when topical fluorides exceeding 500 ppm are applied regularly to mature enamel surfaces over years, only the outer 1 µm of enamel shows any increase in F_2_ concentration with enamel just 5 µm deep, showing no significant increase in F concentration at all [110]. The fact that peak Zn concentration is rarely if ever, even in fossil specimens, at the actual surface, but is always immediately deep to the outer enamel surface, is further evidence for minimal Zn uptake from the oral or *post-mortem* environment (see Appendix SA). The implications for the findings of this study are that Zn preservation, distribution and concentration in the outer enamel in fossil teeth more than likely remains stable, and that except for a thin layer of surface enamel, it is tenacious and persists, potentially unaltered, for millions of years. 

### 4.5. Sampling Zinc in Tooth Tissues

Zinc is concentrated to levels in teeth that are much higher than average levels in soils, foodstuffs and water. This, alone, favours its preservation in tooth tissues in most archaeological and palaeontological environments. Except for Ca, P, O and F, Zn is present at higher concentrations than any other element or trace element at specific locations within each of the mineralised tooth tissues. This also favours its preservation and stability over long periods of time. In modern teeth, Zn in secondary dentine as well as Zn in surface enamel is present in the highest concentrations; sampling here can be conducted relatively easily. However, despite high concentrations in the outer enamel, the diffuse nature of Zn distribution here precludes any fine sequential temporal resolution, whatever the sampling methods, beyond the overall enamel formation period. However, with high-resolution sampling methods, such as Laser Ablation-Inductively Coupled Plasma-Time of Flight Mass Spectrometry (LA-ICP-TOFMS), it is now a real possibility that Zn isotopes can be tracked and sampled through cementum, and even perhaps through secondary dentine, from histological sections of teeth when the Zn increments are sufficiently spaced apart to allow good temporal and chronological resolution of annual or other increments [26]. The prospects for sampling Zn in modern teeth, in enamel, dentine and cementum, are good, with perhaps the best potential chrono-resolution in dentine and cementum. This use of Zn preserved in dentine and cementum would allow either shifts or consistencies in diet to be determined over many years beyond dental maturity and so provide additional information to that derived from enamel where, at least in outer enamel, the chrono-resolution is limited to the crown formation period. Within the fossil enamel, and even in fossil dentine, Zn at the EDJ and at the NNL appears to be retained deep within these tissues in a way that resembles that in modern teeth, and so as well as being markers for birth, they might also better retain their isotopic composition here over millions of years. It is also notable that Zn concentration in the outer enamel of deciduous teeth was generally higher than in the permanent teeth studied here, suggesting that they may be a favourable sampling source among modern and fossil teeth. In the future, the high-resolution sampling of Zn isotopes across prenatal enamel and dentine, the NNLs, and across postnatal enamel and dentine, would seem possible, and would then be a further test of any diagenetic change in the isotopic composition within fossil dentine relative to enamel formed at the same time. This might also reveal information about the nursing history of the individual. Zinc in fossil cementum and dentine, however, is the most likely to undergo diagenetic change [61,90], but Zn in the surface or immediate sub-surface enamel of fossil teeth, as well as Zn close to the EDJ, appears to be in the most promising and easily accessible locations for studies intending to sample stable Zn isotopes designed to study diets in the past.

## 5. Conclusions

Our results show that Zn concentration in outer enamel is universally high across all the deciduous, permanent and fossil hominoid teeth studied. Where secondary dentine has formed, or is preserved, Zn concentrations are also higher than in primary dentine. Besides enamel and dentine, cementum layers also contain Zn at greater concentrations than the underlying root dentine. It therefore may seem paradoxical that the tooth surfaces directly exposed to the external environment *post-mortem*, the enamel surface, root surface and pulp cavity margins, best preserve Zn at the highest concentrations and that Zn in these locations appears to persist in the fossil record, apparently little altered, if at all, in inner enamel, despite considerable diagenetic alteration to many of the other elemental components of fossilised enamel and dentine. The thickness of the Zn-enriched surface enamel layer was observed not to be proportionate in thickness to the regional linear enamel thickness and varied considerably between and within teeth in both the maximum Zn concentration measured in outer enamel (197–1743 ppm), and in the rate of the exponential rise of concentration towards the surface. Zinc levels at the cusp, mid-crown and cervix of the same tooth may vary but mid-crown measurements are as good a representation of the overall average concentrations as any, and perhaps the most consistently high. The Zn concentration range and maximum values in outer enamel overlap in the modern human, great ape and fossil hominoid deciduous and permanent tooth samples studied here. Zinc laid down prenatally and at the NNL and along the EDJ is tenacious and as we show can even be preserved in fossil teeth and used as a marker of birth, with the caveat that Zn lines in enamel and dentine may also be indicative of other stress events in life. New high-resolution sampling and analytical techniques now enable access to the chronological record of Zn laid down in dentine and cementum layers through life with good prospects for tracking dietary shifts, stress events and seasonal changes far back into the archaeological and fossil records. 

## Figures and Tables

**Table 1:** List of specimens used in the present study.

Taxon	Specimen	Specimen Code (Graphs)	Site/Collection	Tooth	Thin Section Thickness (µm)	Resolution (µm)	Dwell Time (ms)	Total Scan Time
Fossils								
*Ekembo nyanzae*	KNM-RU 1721	-	Rusinga Island, Kenya	URM1	120	15	10	55 min
*Ekembo heseloni*	KNM-RU individual IV	S89	Rusinga Island, Kenya	LRdm2	120	15 1.5	10	36 min 1.9 h
*Ekembo heseloni*	KNM-RU individual IV	S90	Rusinga Island, Kenya	LLM1	110	10 1.5	4 10	28 min 1.9 h
*Hispanopithecus laietanus*	IPS 1781	S46	Can Llobateres, Spain	UM1	100	15	10	2.3 h
*Australopithecus anamensis*	KNM-KP 30748	S205	Kanapoi, Kenya	LP4 (frag.)	100	10	10	3.1 h
*Paranthropus robustus*	SK 835	S40	Swartkrans, South Africa	UM3	100	10	10	2.3 h
Neanderthal	Amud 7	S44	Amud Cave, Israel	Udm1	100	10 2.7	10 3	2.3 h 2.2 h
early *Homo sapiens*	Skhūl IV	S45	Skhūl, Wadi el-Mughara, Mount Carmel, Israel	LLM3	100	15	10	2.8 h
Modern specimens								
*Pongo*	UCL-CA28JS7	S16	UCL Elliot Smith	URdm2	80–100	15	10	1.9 h
*Pongo*	UCL-CA28JS7	S18	UCL Elliot Smith	URdm2	80–100	12	10	1.9 h
*Pongo*	UCL-CA28JS3	S23	UCL Elliot Smith	ULC	100	25	10	3.0 h
*Gorilla*	UCL-CA1F1472	S19	UCL Elliot Smith	LRdm2	80–100	15	10	2.1 h
*Gorilla*	UCL-CA1F1472	S20	UCL Elliot Smith	LLdc	100	15	10	1.5 h
*Gorilla*	UCL-CA4	S27	UCL Elliot Smith	LRM2	100	20	10	2.6 h
*Gorilla*	UCL-CA4	S28	UCL Elliot Smith	LRM2	100	20	10	2.6 h
*Gorilla*	UCL-CA4	S29	UCL Elliot Smith	LRM3	100	20	10	2.0 h
*Pan*	UCL-CA11	S22	UCL Elliot Smith	LLM1	100	15	10	2.2 h
*Pan*	UCL-CA19B	S31	UCL Elliot Smith	LLM3	100	20	10	1.5 h
*Pan*	UCL-CA198	S30	UCL Elliot Smith	LLM1	100	20	10	1.4 h
*Pan*	UCL-CA11D	S34	UCL Elliot Smith	LM3	100	10	10	1.3 h
*Pan*	UCL-CA14E		UCL Elliot Smith	UI1	100	5	25	2.0 h
*Homo sapiens*	NCL 2	S3	NCL Anat	Udm2	120	2.5	10	2.1 h
*Homo sapiens*	modern	S6	UCL Anat	Ldc	80–100	10	10	1.3 h
*Homo sapiens*	modern	S7	UCL Anat	Udm1	80–100	10 1	10 10	1.6 h 50 min
*Homo sapiens*	modern	S8	UCL Anat	Udm2	80–100	10	10	2.5 h
*Homo sapiens*	modern	S9	UCL Anat	Udm2	80–100	15	10	1.0 h
*Homo sapiens*	modern	S50	UCL Anat	LLdc	80–100	1	15	1.1 h
*Homo sapiens*	modern	S51	UCL Anat	URdm1	80–100	1	15	1.1 h
*Homo sapiens*	modern	S209	UCL Anat	Udm2	80–100	2.5	4	3.2 h
*Homo sapiens*	modern	Tr	UCL Anat	Udm2	80–100	10	10	1.0 h
*Homo sapiens*	modern	B40	UCL Anat	LM3	80–100	4	10	1.8 h
*Homo sapiens*	modern	B11	UCL Anat	UC	80–100	11	7	4.3 h
*Homo sapiens*	modern	B9CD2	UCL Anat	UC	80–100	5 3	2 8	23 min 2.6 h

## Data Availability

Raw data supporting the reported results are available on Zenodo (https://zenodo.org/record/8403018, accessed on 1 November 2023). The R code used to produce Appendix SB is available online (https://gitlab.com/f-santos/dean-et-al-2023-supporting-files, accessed on 1 November 2023). Requests for additional data not presented in the Appendix and Supplementary Materials should be made to the first and/or last authors (M.C.D. and A.L.C.).

## Supplementary Materials

The following supporting information can be downloaded at: https://www.mdpi.com/article/10.3390/biology12121455/s1, Figure S1: Stacked chart showing the percentages of the width Zn band (purple), the distance from the Zn peak to the outer enamel surface (dark green), and distance from the start of the Zn band (“take-off”) to the Zn peak, all variables in relation to the cuspal enamel thickness. Figure S2: Stacked chart showing the percentages of the width Zn band (purple), the distance from the Zn peak to the outer enamel surface (dark green), and distance from the start of the Zn band (“take-off”) to the Zn peak, all variables in relation to the mid-lateral enamel thickness. Figure S3: Stacked chart showing the percentages of the width Zn band (purple), the distance from the Zn peak to the outer enamel surface (dark green), and distance from the start of the Zn band (“take-off”) to the Zn peak, all variables in relation to the cervical enamel thickness. Figure S4: Stacked chart showing the absolute measurements in enamel thickness (µm; blue) and Zn peak concentrations (µm; green) for cuspal enamel. Figure S5: Stacked chart showing the absolute measurements in enamel thickness (µm; blue) and Zn peak concentrations (µm; green) for mid-lateral enamel. Figure S6: Stacked chart showing the absolute measurements in enamel thickness (µm; blue) and Zn peak concentrations (µm; green) for cervical enamel. Table S1: Raw data and graphs for enamel thickness and width of the outer enamel Zn-rich band. Appendix SA: Methods and detailed Zn maps used for measuring enamel thickness at the cuspal, mid-lateral and cervical portions of the crowns, Zn concentrations and width of the Zn-enriched band at the outer enamel surface. Appendix SB: Statistical methods and results for the correlation analyses between the measured variables. References [82,83,84,111,112] are cited in the supplementary materials.
