# Supplementary material for: The Distribution and Biogenic Origins of Zinc in the Mineralised Tooth Tissues of Modern and Fossil Hominoids: Implications for Life History, Diet and Taphonomy
Source: Biology (Basel). 2023 Nov 21;12(12):1455. doi: 10.3390/biology12121455 (PMC10740576; doi:10.3390/biology12121455)
Supplement: Supplementary file 1 [file biology-12-01455-s001.zip › Dean_et_al_2023_Biology_Appendix SA.pdf]

**Appendix SA**

# Quantitative assessment of Zn in the outer enamel surface in relation to total enamel thickness

Dean et al. *Biology*

# Outline

Methods ………………………………………………………………………… 3

Zinc maps and ROIs

Modern deciduous teeth ……………………………………………………………. 4

Modern permanent teeth …………………………………………………………… 16

Fossil deciduous teeth ……………………………………………………………… 22

Fossil permanent teeth ……………………………………………………………… 25

# Methods

For each specimen, the Zn map was recorded as a 32-bit tiff file in Geopixe for Proposals I-20160686 and I-20180047 (Maia detector), and in HDIP for Proposals I-20190203 and I-20210133 (Vortex EM silicon drift detectors).

The Zn map was then opened in ImajeJ (version 1.53t), the image scale was set according to the scan resolution (e.g., 1 pixel equals 10 μm), and the contrast and brightness adjusted to best visualize the Zn variations.

Then for each of the enamel areas of interest (cuspal, mid-lateral and cervical), a segmented line going in the direction of the prisms was drawn from beyond the enamel-dentine junction (EDJ) in the dentine, to in the air, beyond the outer enamel surface (OES). To make sure the ROIs respect the direction of the prisms, the line started deeper in the dentine than the EDJ, and ended in the air, beyond the OES.

These 3 regions of interest (ROIs) were saved using the ROI manager, and an image (see below) was saved as a record. The raw data along the 3 ROIs were saved as a csv file, and a multiplot was drawn and saved as a pdf for easy visualization of the data and detection of mistakes.

For relative enamel thickness, the measurements were restricted to start from the EDJ and end on the OES, and Zn contents were plotted against a percentage of distance from the EDJ (0%) to the OES (100%).

Summary graphs were then build in Excel by compiling the raw data of all specimens.

From the subset of values for relative enamel thickness, the maximum Zn value (Zn peak) was computed in Excel, as well as the width of the Zn-enriched band, the distance between "take-off" and the Zn peak, and the distance between the Zn peak and the OES, all in relation to total enamel thickness (absolute and relative [percentage] values reported hereafter).

**Table - Descriptive statistics in enamel thickness, maximal Zn concentration (peak) and width of the Zn-enriched band of enamel in the three anatomical regions (cuspal, mid-lateral, and cervical).**

| | Enamel thickness [µm] | Zn peak (ppm) | Width of Zn-enriched band (µm) |
|---|---|---|---|
| **Cuspal** | Min = 44<br>Max = 2820<br>Mean = 962.2<br>SD = 614.1 | Min = 434<br>Max = 1743<br>Mean = 879.2<br>SD = 334.3 | Min = 16<br>Max = 950<br>Mean = 388.7<br>SD = 219.6 |
| **Mid-lateral** | Min = 38<br>Max = 1970<br>Mean = 806.28<br>SD = 484.1 | Min = 197<br>Max = 1717<br>Mean = 915.9<br>SD = 412.4 | Min = 17<br>Max = 950<br>Mean = 412.9<br>SD = 203.6 |
| **Cervical** | Min = 14<br>Max = 840<br>Mean = 370.4<br>SD = 208.8 | Min = 393<br>Max = 1602<br>Mean = 821.6<br>SD = 351.3 | Min = 13<br>Max = 560<br>Mean = 272.2<br>SD = 123.5 |
| **Overall** | Min = 14 (cervical)<br>Max = 2820 (cuspal) | Min = 197 (mid-lateral)<br>Max = 1743 (cuspal) | Min = 13 (cervical)<br>Max = 950 (cuspal) |

# Modern deciduous teeth

Tr (*H. sapiens*) Udm2

Resolution: 10 µm

| | Enamel thickness [µm] | Zn peak (ppm) | Zn peak width (µm [%]) |
|---|---|---|---|
| **Cuspal** | 1120 | 896 | 400 [35.7%] |
| **Mid-lateral** | 910 | 872 | 490 [53.8%] |
| **Cervical** | 330 | 798 | 320 [97.0%] |

| | "Take-off" to Zn peak (µm [%]) | Zn peak to OES (µm [%]) |
|---|---|---|
| **Cuspal** | 360 [32.1%] | 40 [3.6%] |
| **Mid-lateral** | 440 [48.4%] | 50 [5.5%] |
| **Cervical** | 300 [90.9%] | 20 [6.1%] |

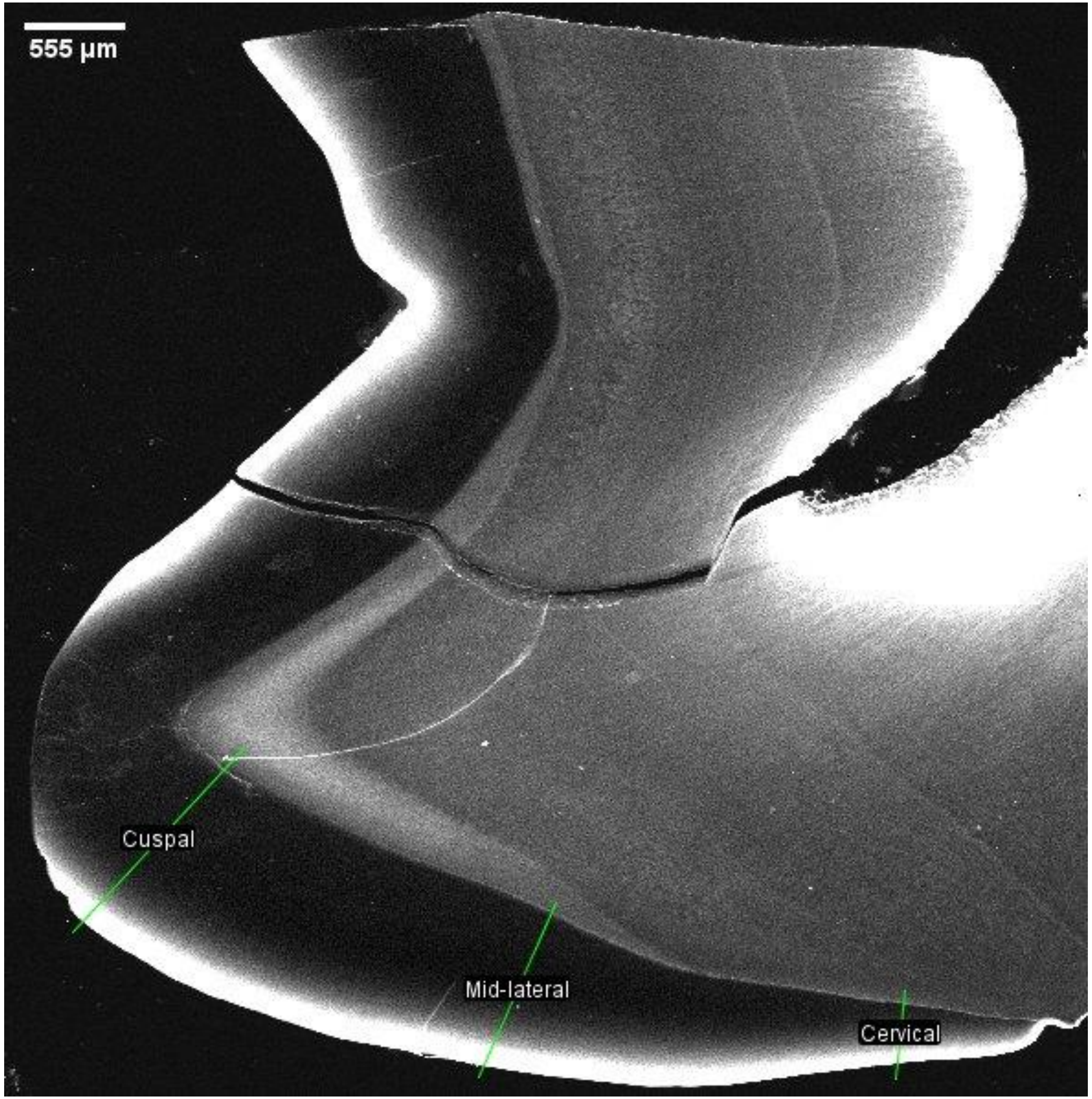

Modern deciduous teeth

S6 (*H. sapiens*) Ldc

Resolution: 10 µm

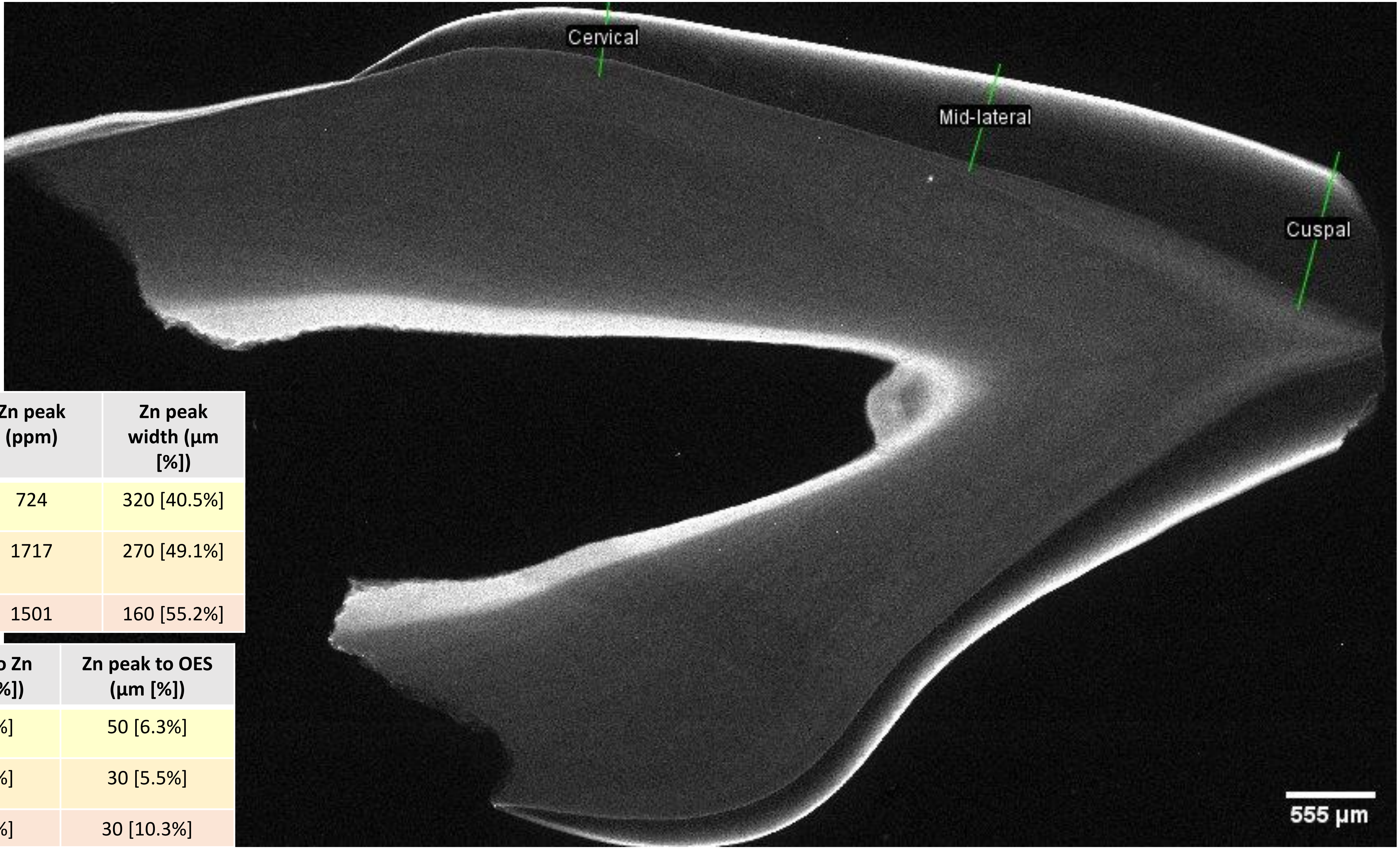


| | Enamel thickness [µm] | Zn peak (ppm) | Zn peak width (µm [%]) |
|---|---|---|---|
| **Cuspal** | 790 | 724 | 320 [40.5%] |
| **Mid-lateral** | 550 | 1717 | 270 [49.1%] |
| **Cervical** | 290 | 1501 | 160 [55.2%] |

| | "Take-off" to Zn peak (µm [%]) | Zn peak to OES (µm [%]) |
|---|---|---|
| **Cuspal** | 270 [34.2%] | 50 [6.3%] |
| **Mid-lateral** | 240 [43.6%] | 30 [5.5%] |
| **Cervical** | 130 [44.8%] | 30 [10.3%] |

Modern deciduous teeth

S7 (*H. sapiens*) Udm1

Resolution: 10 μm

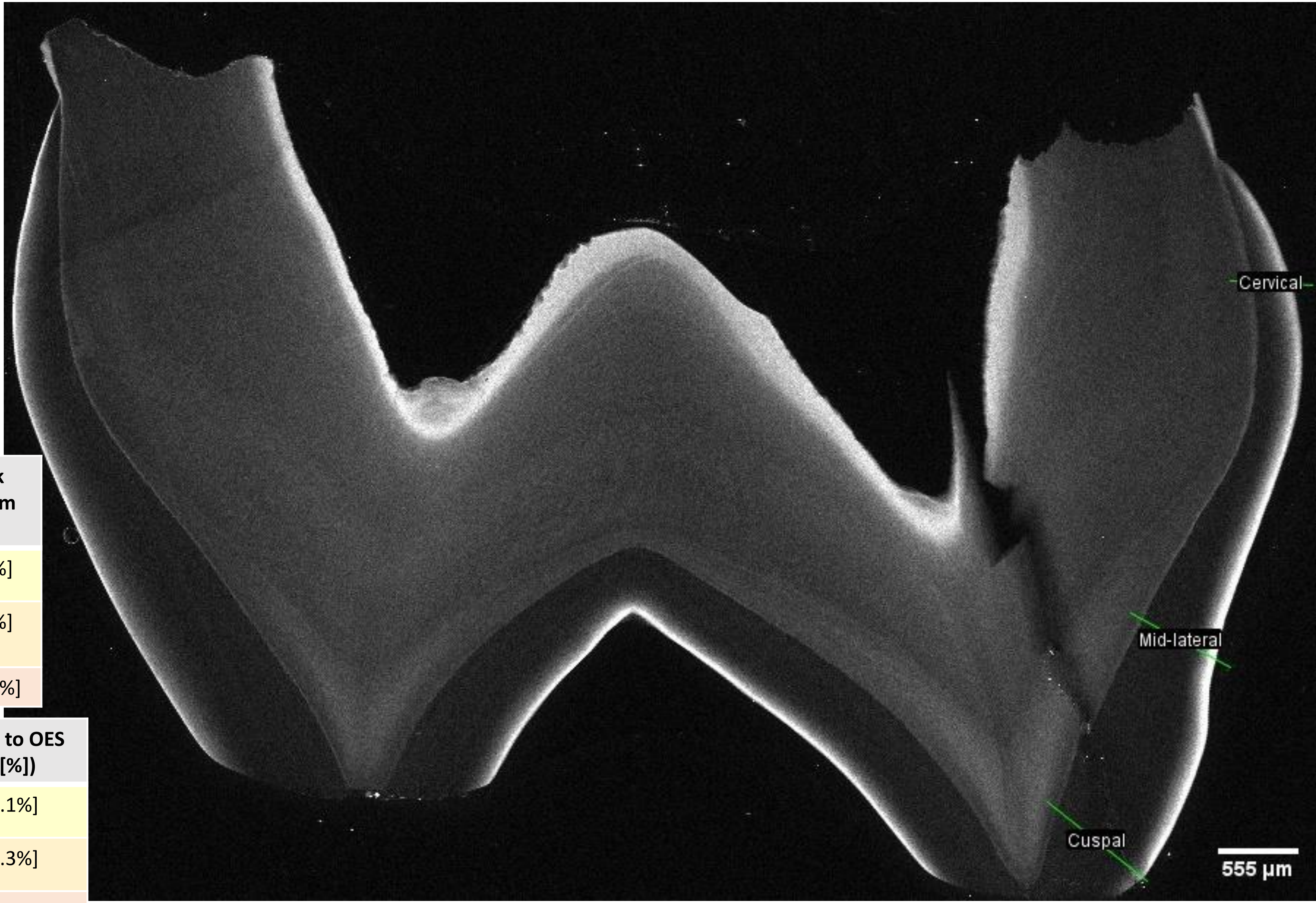


| | Enamel thickness [μm] | Zn peak (ppm) | Zn peak width (μm [%]) |
|---|---|---|---|
| **Cuspal** | 740 | 451 | 200 [27%] |
| **Mid-lateral** | 480 | 313 | 240 [50%] |
| **Cervical** | 270 | 574 | 210 [77.8%] |

| | "Take-off" to Zn peak (μm [%]) | Zn peak to OES (μm [%]) |
|---|---|---|
| **Cuspal** | 170 [23%] | 30 [4.1%] |
| **Mid-lateral** | 200 [41.7%] | 40 [8.3%] |
| **Cervical** | 160 [59.3%] | 50 [18.5%] |

Modern deciduous teeth

S8 (*H. sapiens*) Udm2

Resolution: 10 µm

| | Enamel thickness [µm] | Zn peak (ppm) | Zn peak width (µm [%]) |
|---|---|---|---|
| **Cuspal** | 870 | 1050 | 380 [43.7%] |
| **Mid-lateral** | 600 | 1289 | 320 [53.3%] |
| **Cervical** | 250 | 1179 | 230 [92%] |

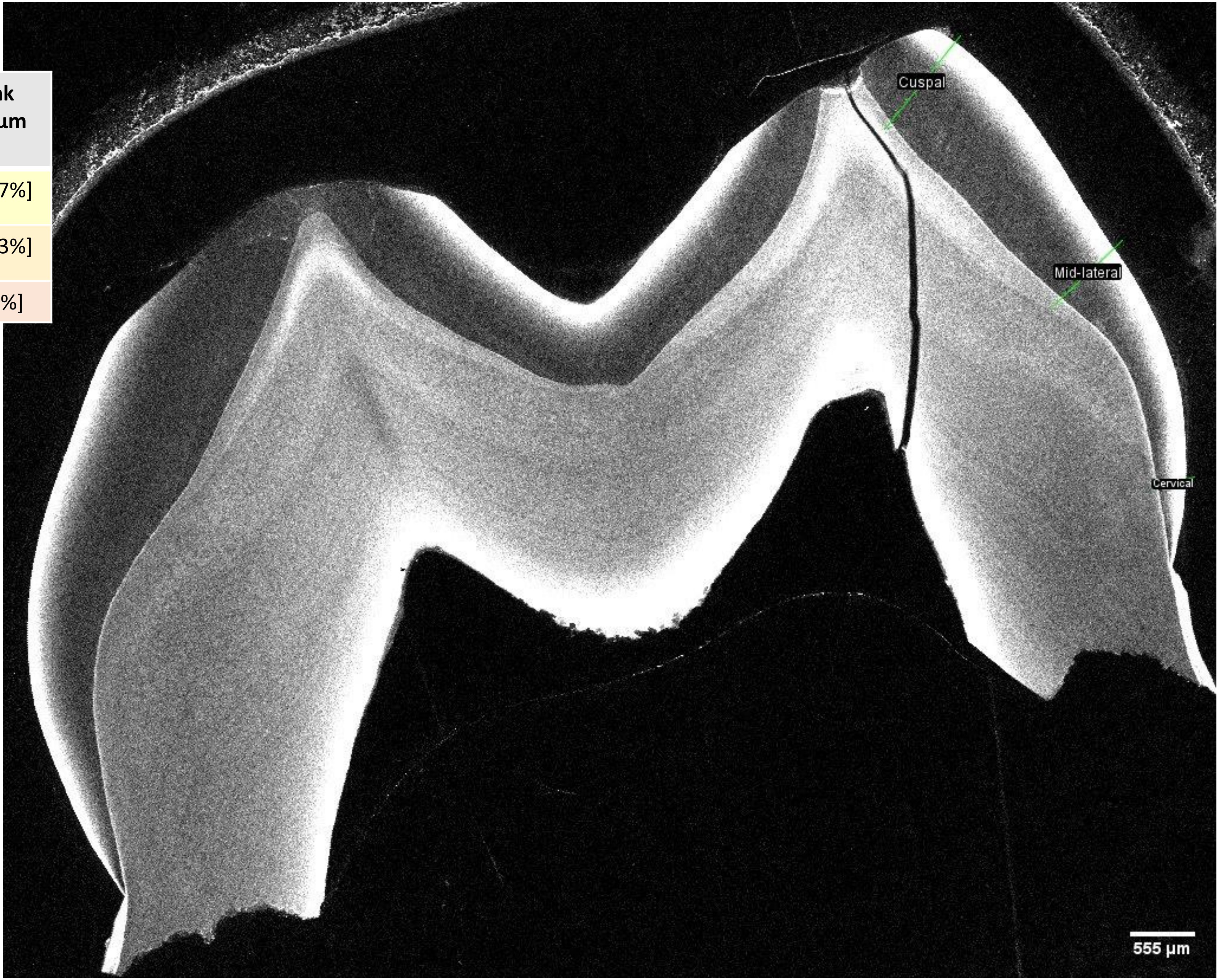


| | "Take-off" to Zn peak (µm [%]) | Zn peak to OES (µm [%]) |
|---|---|---|
| **Cuspal** | 350 [40.2%] | 30 [3.4%] |
| **Mid-lateral** | 300 [50%] | 20 [3.3%] |
| **Cervical** | 200 [80%] | 30 [12%] |

S9 (*H. sapiens)* Udm2

Resolution: 15 µm

| | Enamel thickness [µm] | Zn peak (ppm) | Zn peak width (µm [%]) |
|---|---|---|---|
| **Cuspal** | 735 | 904 | 345 [46.9%] |
| **Mid-lateral** | 795 | 1314 | 375 [47.2%] |
| **Cervical** | 240 | 1002 | 210 [87.5%] |

| | "Take-off" to Zn peak (µm [%]) | Zn peak to OES (µm [%]) |
|---|---|---|
| **Cuspal** | 315 [42.9%] | 30 [4.1%] |
| **Mid-lateral** | 315 [39.6%] | 60 [7.5%] |
| **Cervical** | 165 [68.8%] | 45 [18.8%] |

N.B.: The horizontal grey line is a scanning artifact: a few lines of data are missing, but this does not affect the measurements.

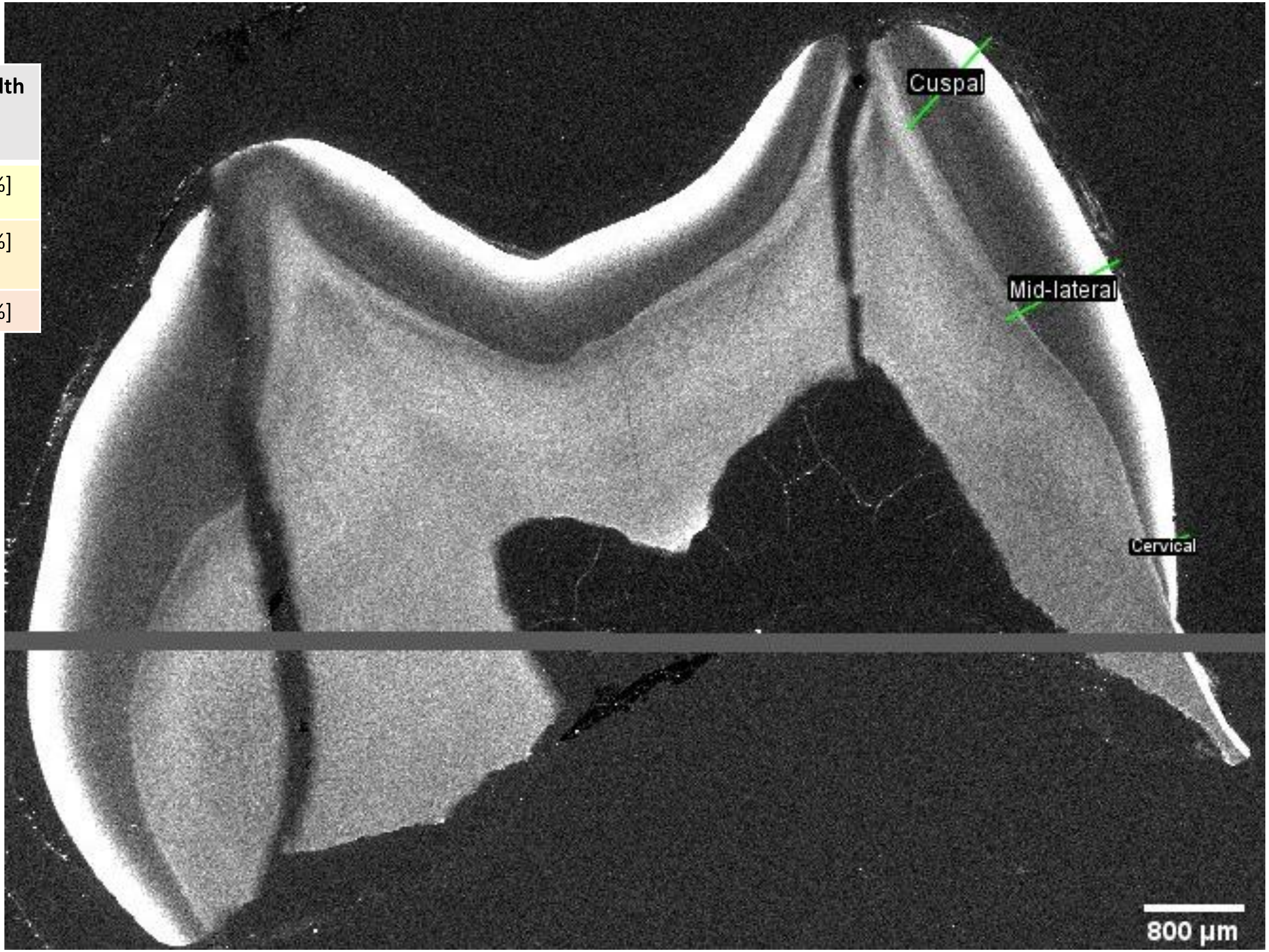

Modern deciduous teeth

S50 (*H. sapiens*) LLdc

Resolution: 1 µm

| | Enamel thickness [µm] | Zn peak (ppm) | Zn peak width (µm [%]) |
|---|---|---|---|
| **Cuspal** | 49 | 1320 | 17 [34.7%] |
| **Mid-lateral** | 38 | 1437 | 18 [47.4%] |
| **Cervical** | 20 | 1140 | 15 [75%] |

| | "Take-off" to Zn peak (µm [%]) | Zn peak to OES (µm [%]) |
|---|---|---|
| **Cuspal** | 14 [28.6%] | 3 [6.1%] |
| **Mid-lateral** | 15 [39.5%] | 3 [7.9%] |
| **Cervical** | 14 [70%] | 1 [5%] |

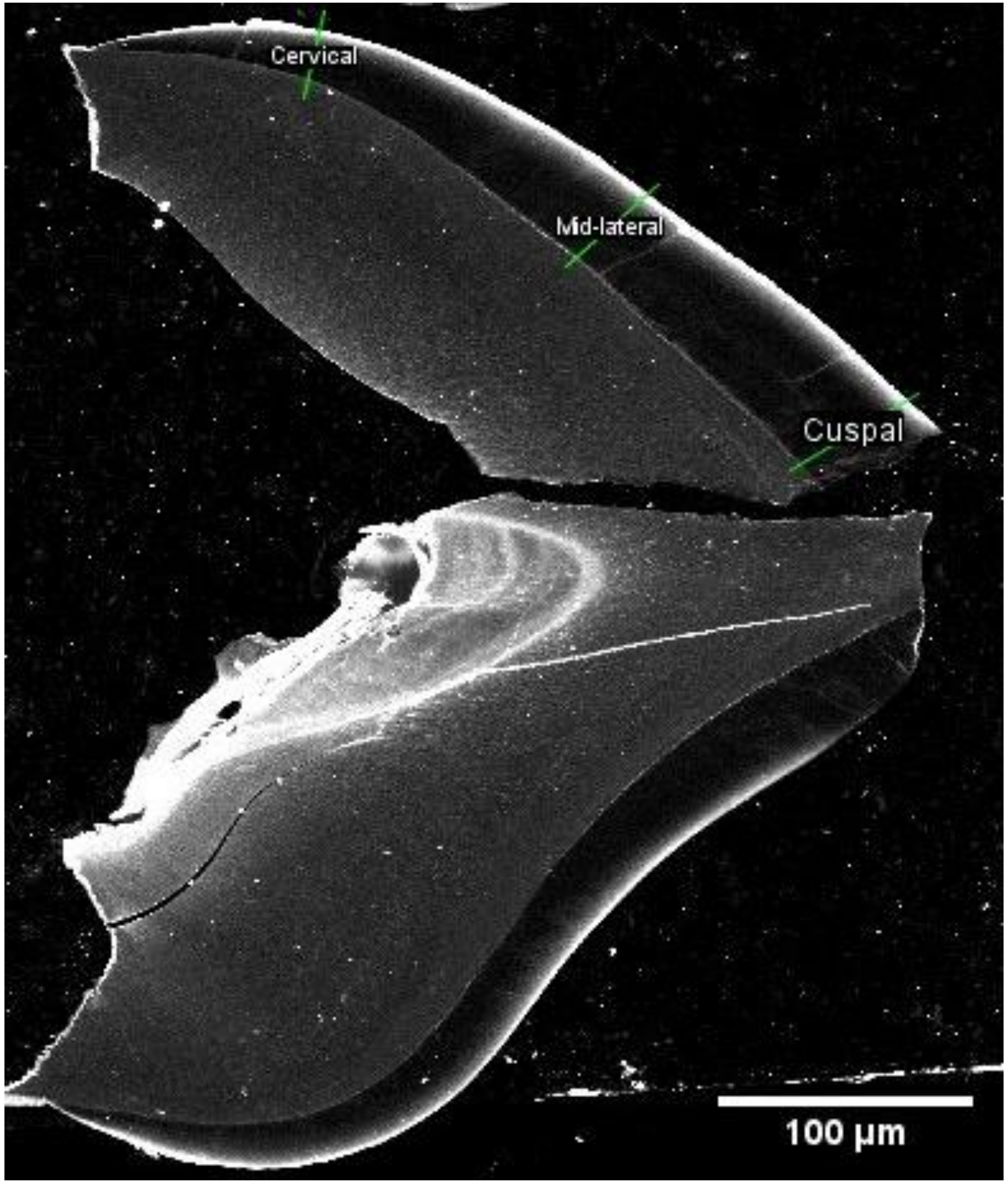

# S51 (*H. sapiens*) URdm1

Resolution: 1 µm

| | Enamel thickness [µm] | Zn peak (ppm) | Zn peak width (µm [%]) |
|---|---|---|---|
| **Cuspal** | 44 | 434 | 16 [36.4%] |
| **Mid-lateral** | 42 | 885 | 17 [40.5%] |
| **Cervical** | 14 | 528 | 13 [92.9%] |

| | "Take-off" to Zn peak (µm [%]) | Zn peak to OES (µm [%]) |
|---|---|---|
| **Cuspal** | 11 [25%] | 5 [11.4%] |
| **Mid-lateral** | 14 [33.3%] | 3 [7.1%] |
| **Cervical** | 9 [64.3%] | 4 [28.6%] |

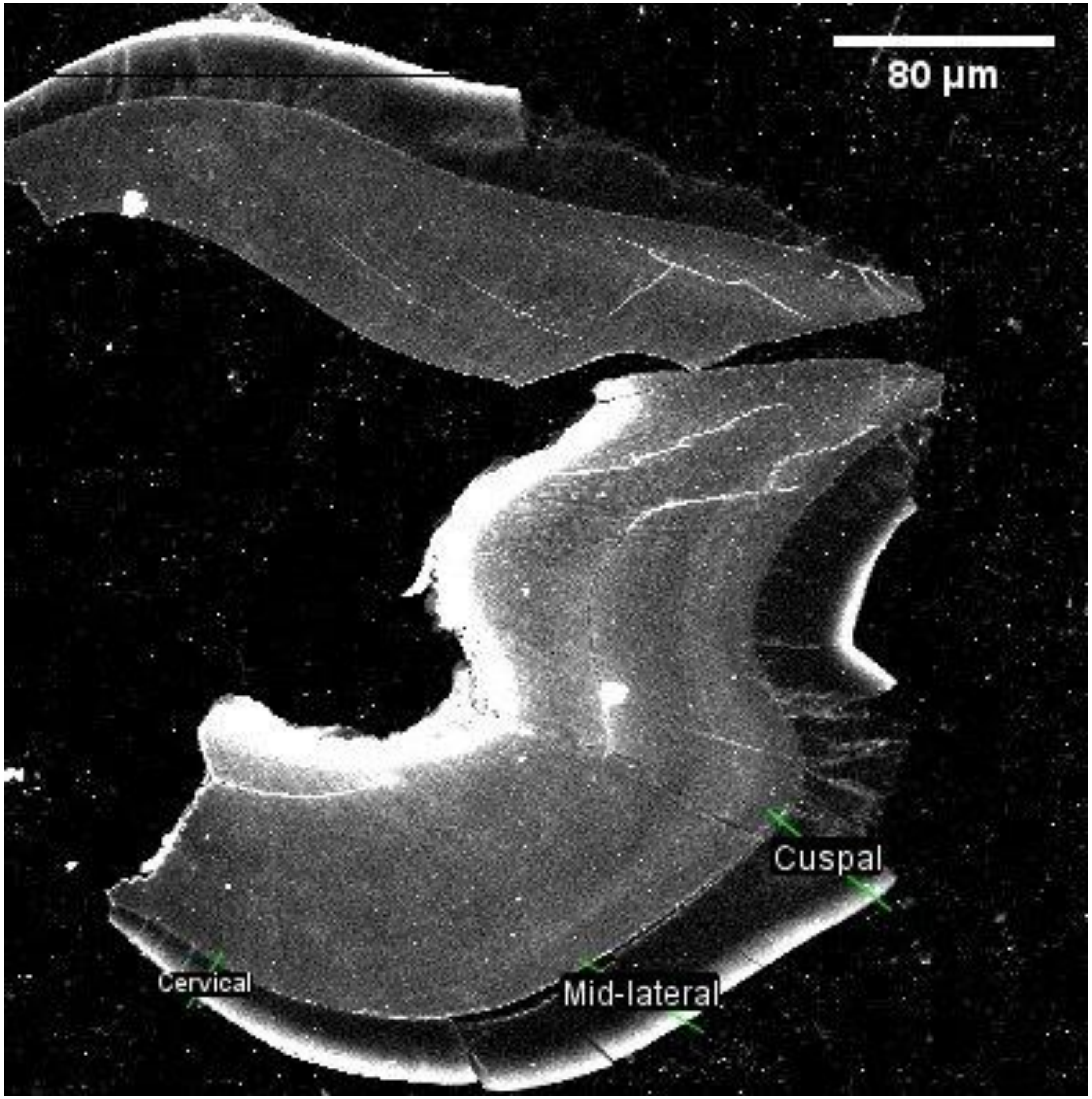

## S209 (*H. sapiens*) Udm2

Resolution: 2.5 µm

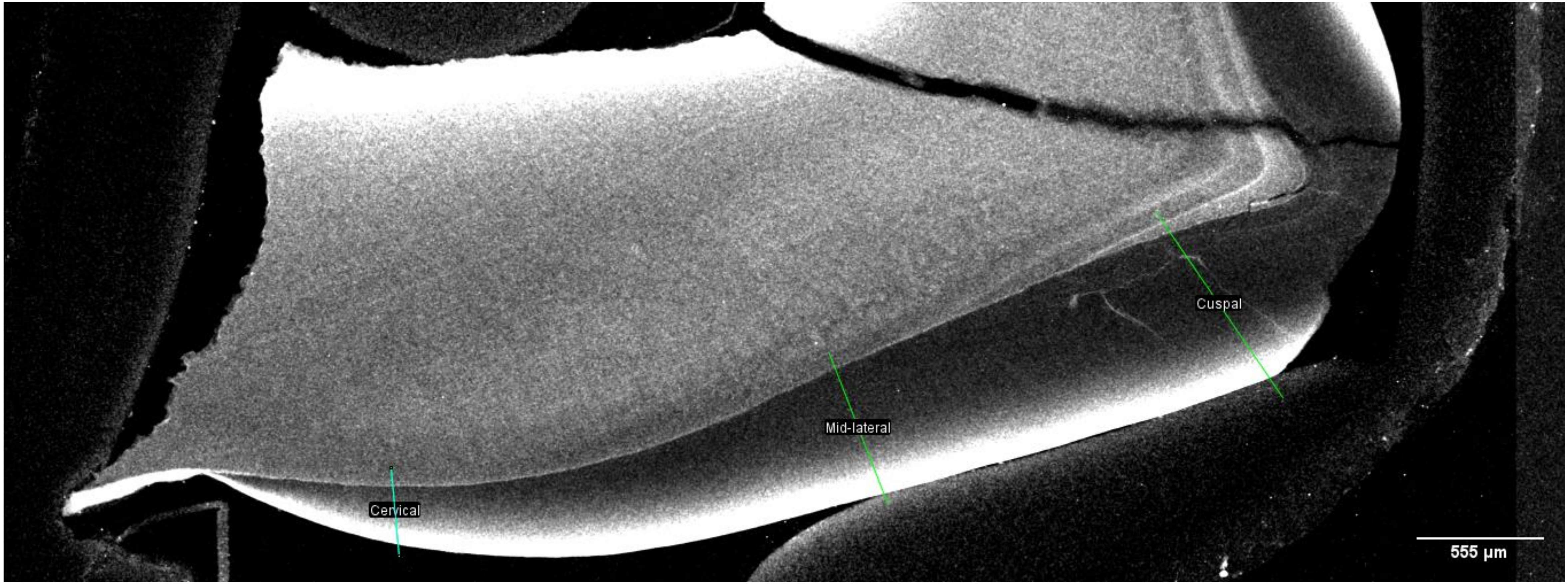


| | Enamel thickness [µm] | Zn peak (ppm) | Zn peak width (µm [%]) | "Take-off" to Zn peak (µm [%]) | Zn peak to OES (µm [%]) |
|---|---|---|---|---|---|
| **Cuspal** | 770 | 986 | 282.5 [36.7%] | 267.5 [34.7%] | 15 [1.9%] |
| **Mid-lateral** | 590 | 871 | 317.5 [53.8%] | 305 [51.7%] | 12.5 [2.1%] |
| **Cervical** | 260 | 981 | 240 [92.3%] | 220 [84.6%] | 20 [7.7] |

# S16 *Pongo* CA28JS7 URdm2

Resolution: 15 µm

| | Enamel thickness [µm] | Zn peak (ppm) | Zn peak width (µm [%]) |
|---|---|---|---|
| **Cuspal** | 750 | 1456 | 390 [52%] |
| **Mid-lateral** | 660 | 1383 | 390 [59.1%] |
| **Cervical** | 300 | 1000 | 255 [85%] |

| | "Take-off" to Zn peak (µm [%]) | Zn peak to OES (µm [%]) |
|---|---|---|
| **Cuspal** | 360 [48%] | 30 [4%] |
| **Mid-lateral** | 330 [50%] | 60 [9.1%] |
| **Cervical** | 225 [75%] | 30 [10%] |

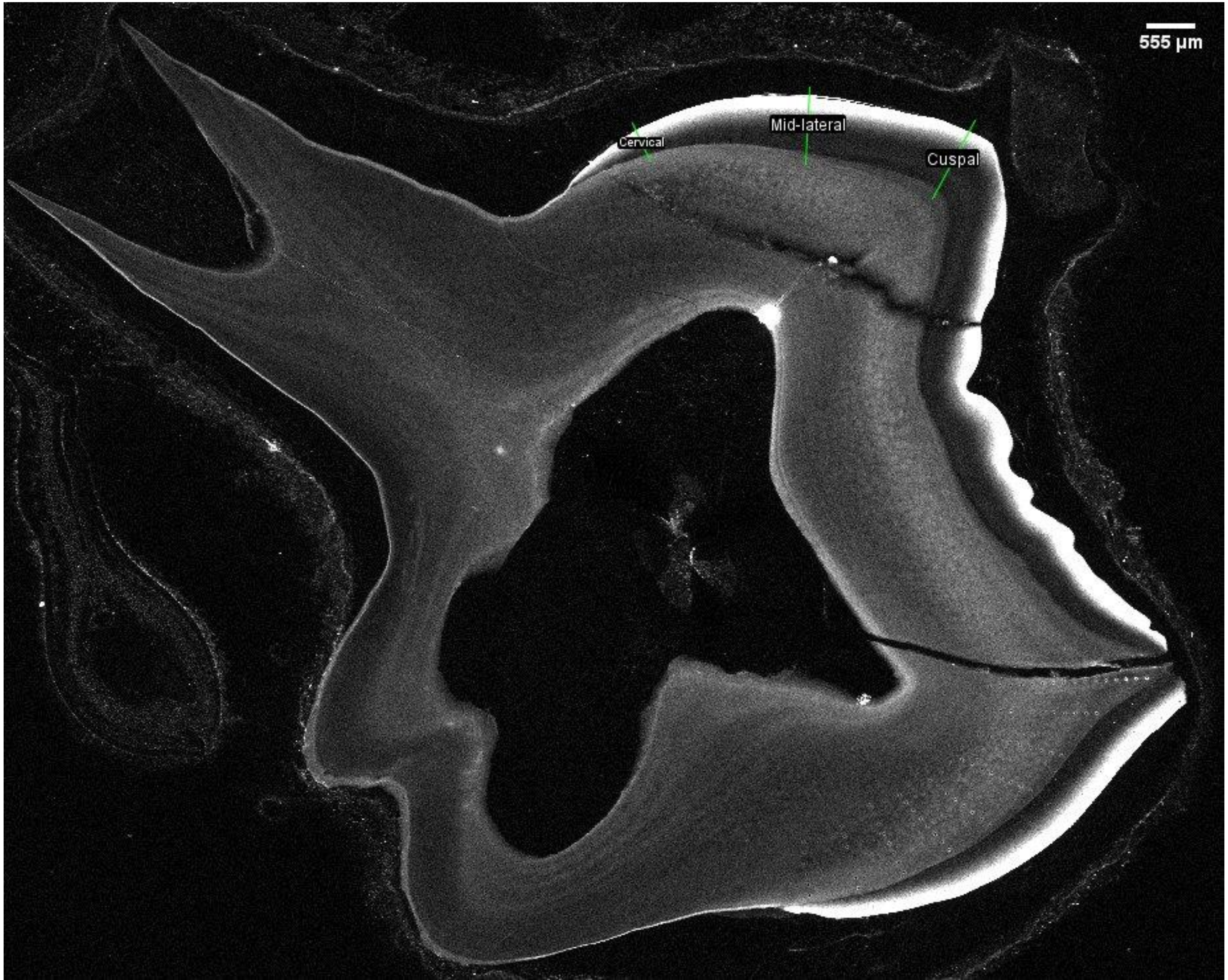

Modern deciduous teeth

# S18 *Pongo* CA28JS7 UCL JS6 LRdm2

Resolution: 12 μm

| | Enamel thickness [μm] | Zn peak (ppm) | Zn peak width (μm [%]) |
|---|---|---|---|
| **Cuspal** | 792 | 1743 | 336 [42.4%] |
| **Mid-lateral** | 612 | 1222 | 348 [56.9%] |
| **Cervical** | 252 | 539 | 240 [95.2%] |

| | "Take-off" to Zn peak (μm [%]) | Zn peak to OES (μm [%]) |
|---|---|---|
| **Cuspal** | 300 [37.9%] | 36 [4.5%] |
| **Mid-lateral** | 312 [51%] | 36 [5.9%] |
| **Cervical** | 156 [61.9%] | 84 [33.3%] |

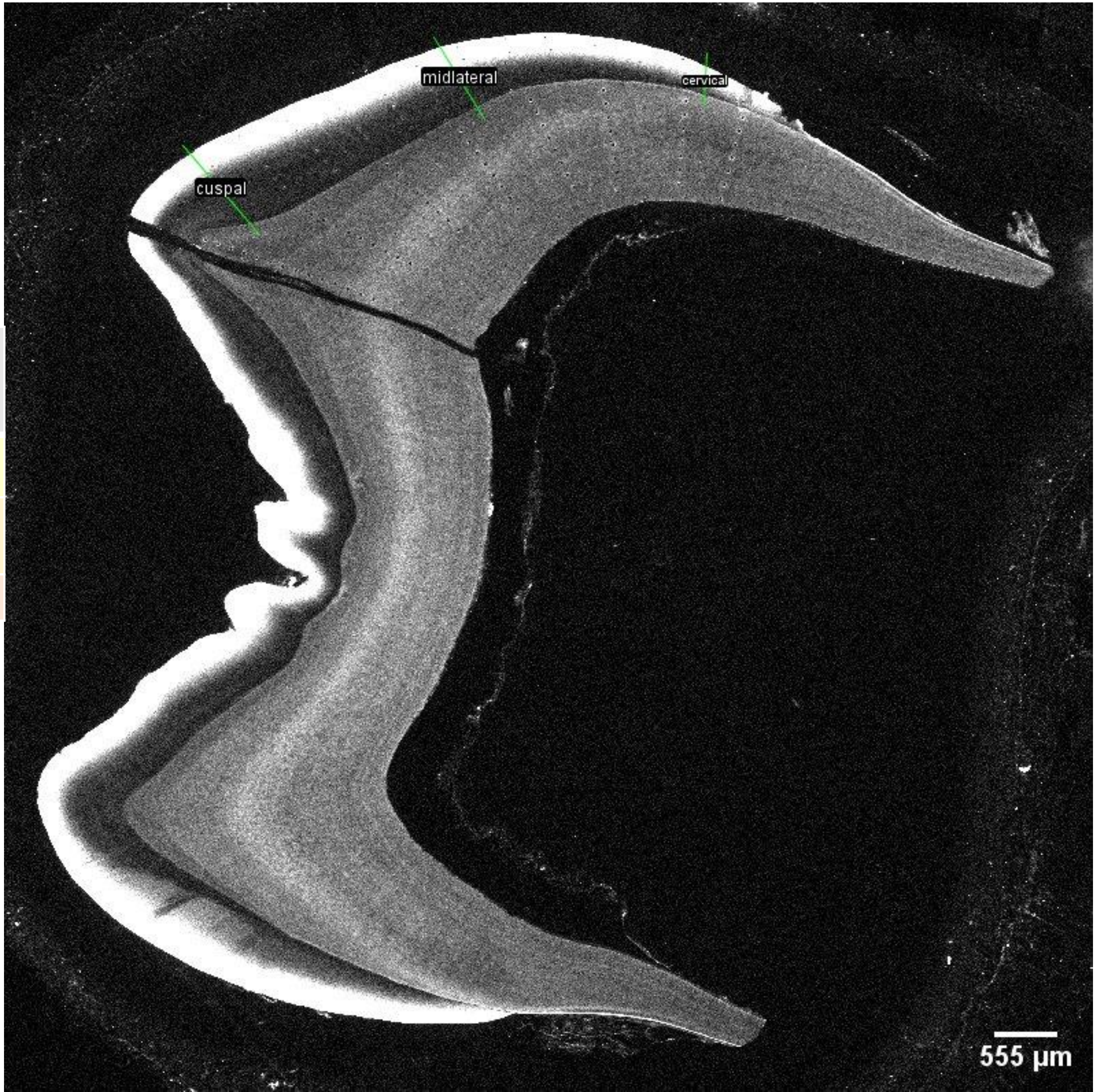

Modern deciduous teeth

S19 *Gorilla* CA1F1472 LRdm2

Resolution: 15 µm

| | Enamel thickness [µm] | Zn peak (ppm) | Zn peak width (µm [%]) |
|---|---|---|---|
| **Cuspal** | 690 | 733 | 255 [37%] |
| **Mid-lateral** | 435 | 197 | 300 [69%] |
| **Cervical** | 300 | 1602 | 240 [80%] |

| | "Take-off" to Zn peak (µm [%]) | Zn peak to OES (µm [%]) |
|---|---|---|
| **Cuspal** | 210 [30.4%] | 45 [6.5%] |
| **Mid-lateral** | 255 [58.6%] | 45 [10.3%] |
| **Cervical** | 210 [70%] | 30 [10%] |

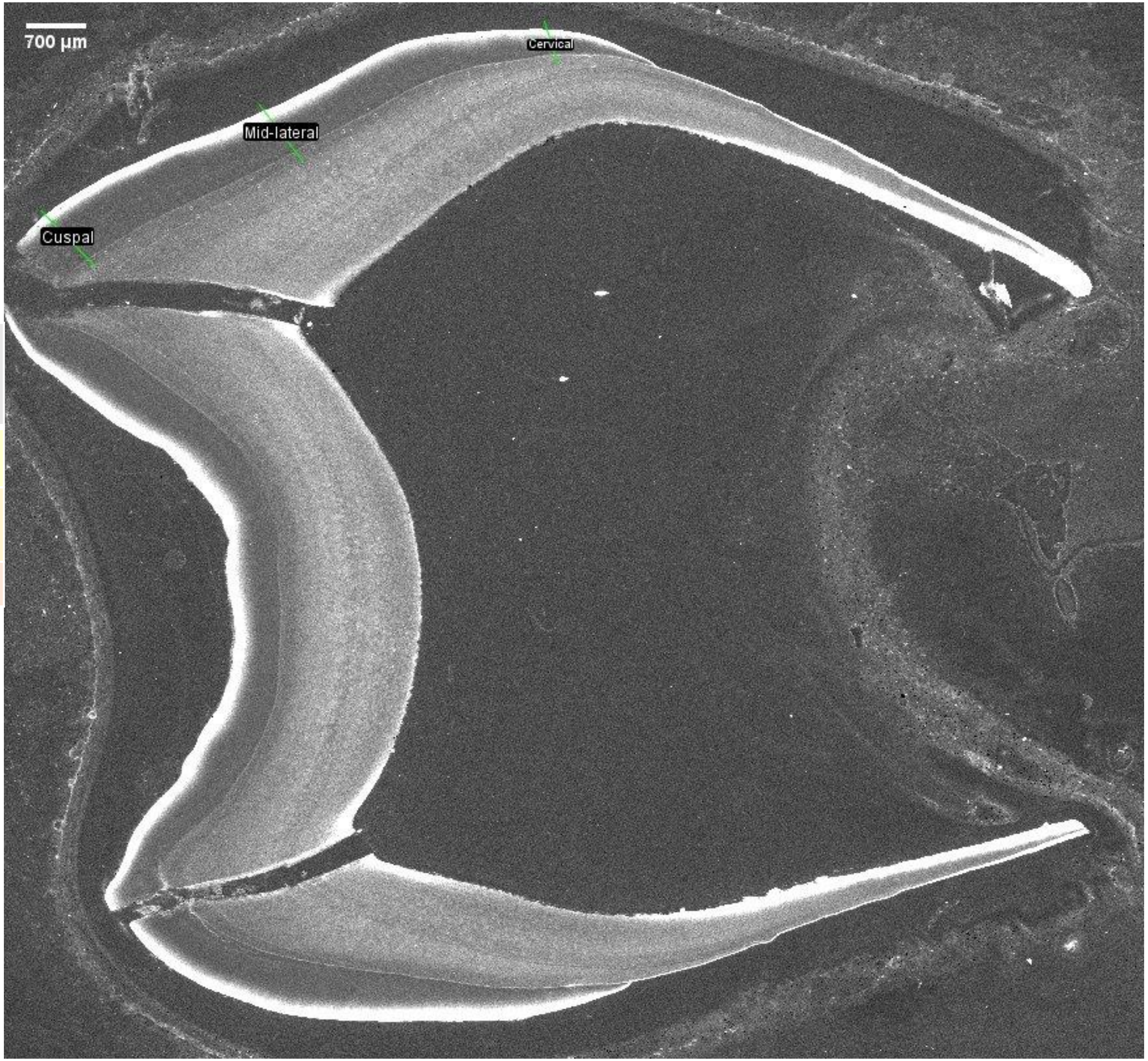

Modern permanent teeth

# S22 *Pan* CA11 LLM1

Resolution: 15 µm

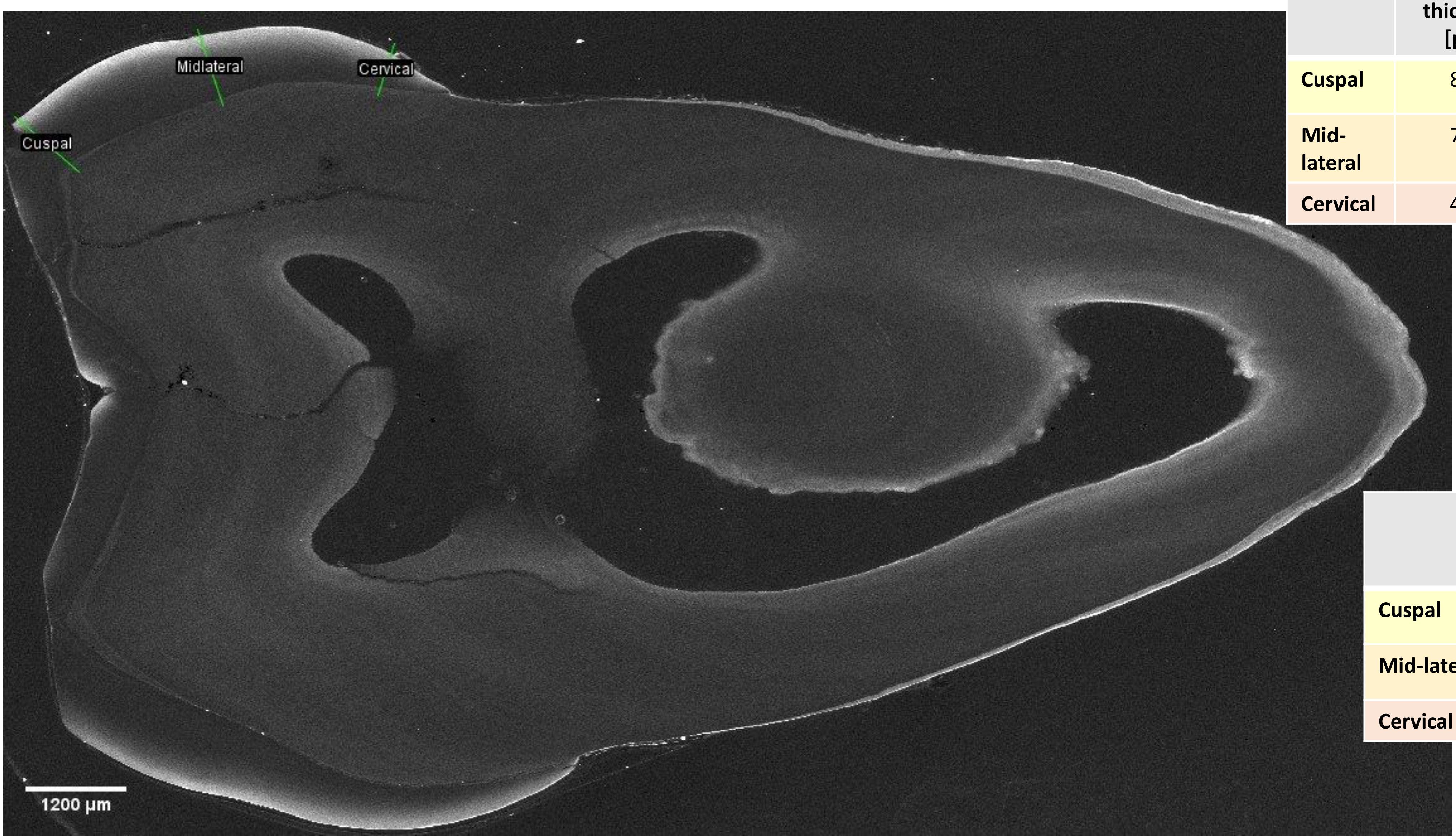


| | Enamel thickness [µm] | Zn peak (ppm) | Zn peak width (µm [%]) |
|---|---|---|---|
| **Cuspal** | 810 | 463 | 345 [42.6%] |
| **Mid-lateral** | 780 | 562 | 510 [65.4%] |
| **Cervical** | 420 | 462 | 330 [78.3%] |

| | "Take-off" to Zn peak (µm [%]) | Zn peak to OES (µm [%]) |
|---|---|---|
| **Cuspal** | 300 [37%] | 45 [5.6%] |
| **Mid-lateral** | 450 [57.7%] | 60 [7.7%] |
| **Cervical** | 285 [67.9%] | 45 [10.7%] |

Modern permanent teeth

# S27 *Gorilla* CA4 LRM1

Resolution: 20 µm

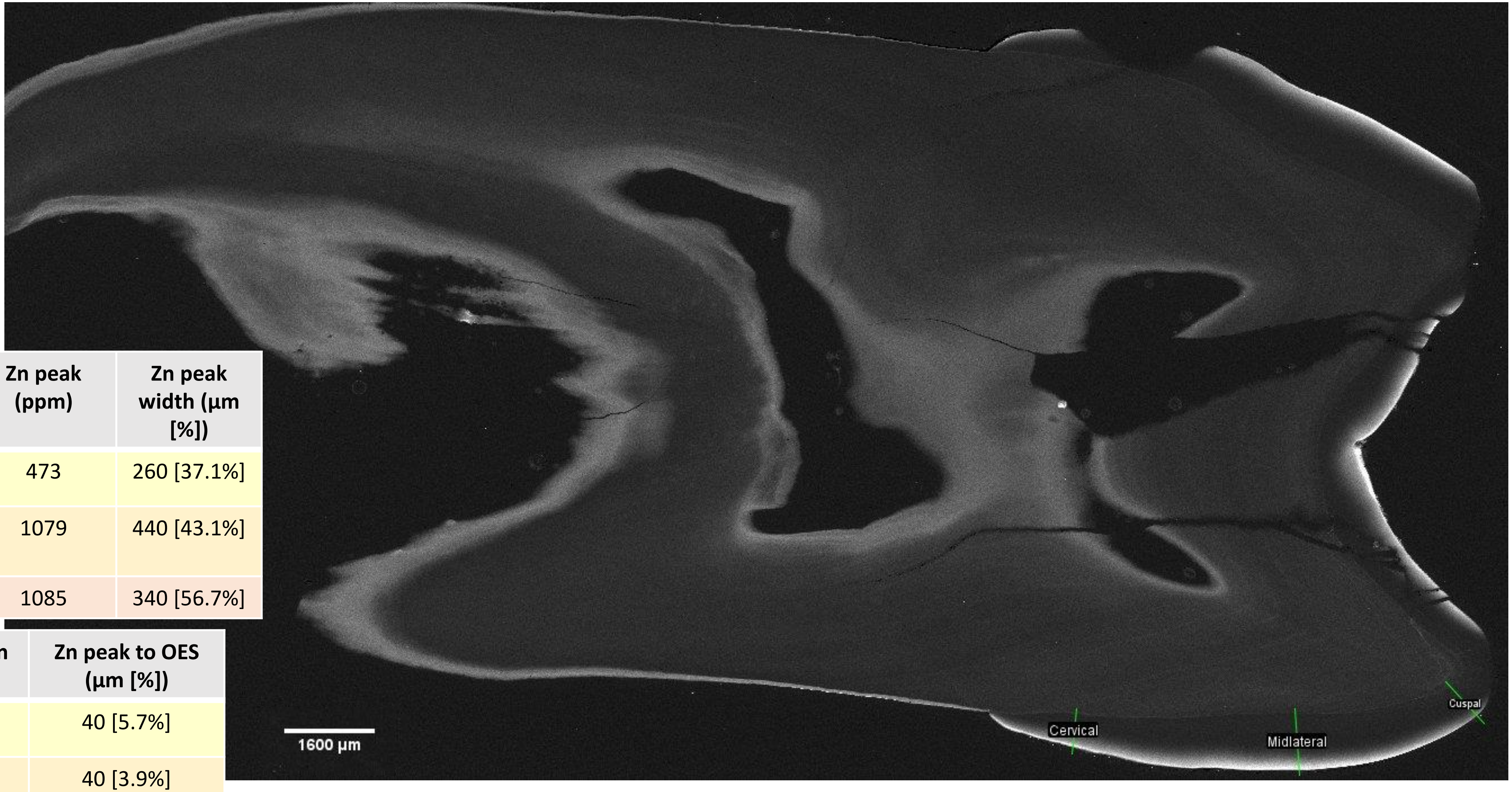


| | Enamel thickness [µm] | Zn peak (ppm) | Zn peak width (µm [%]) |
|---|---|---|---|
| **Cuspal** | 700 | 473 | 260 [37.1%] |
| **Mid-lateral** | 1020 | 1079 | 440 [43.1%] |
| **Cervical** | 600 | 1085 | 340 [56.7%] |

| | "Take-off" to Zn peak (µm [%]) | Zn peak to OES (µm [%]) |
|---|---|---|
| **Cuspal** | 220 [31.4%] | 40 [5.7%] |
| **Mid-lateral** | 400 [39.2%] | 40 [3.9%] |
| **Cervical** | 280 [46.7%] | 60 [10%] |

# S28 *Gorilla* CA4 LRM2

Resolution: 20 µm

| | Enamel thickness [µm] | Zn peak (ppm) | Zn peak width (µm [%]) |
|---|---|---|---|
| **Cuspal** | 1460 | 1120 | 500 [34.2%] |
| **Mid-lateral** | 1620 | 1507 | 480 [29.6%] |
| **Cervical** | 760 | 699 | 360 [47.4%] |

| | "Take-off" to Zn peak (µm [%]) | Zn peak to OES (µm [%]) |
|---|---|---|
| **Cuspal** | 440 [30.1%] | 60 [4.1%] |
| **Mid-lateral** | 440 [27.2%] | 40 [2.5%] |
| **Cervical** | 320 [42.1%] | 40 [5.3%] |

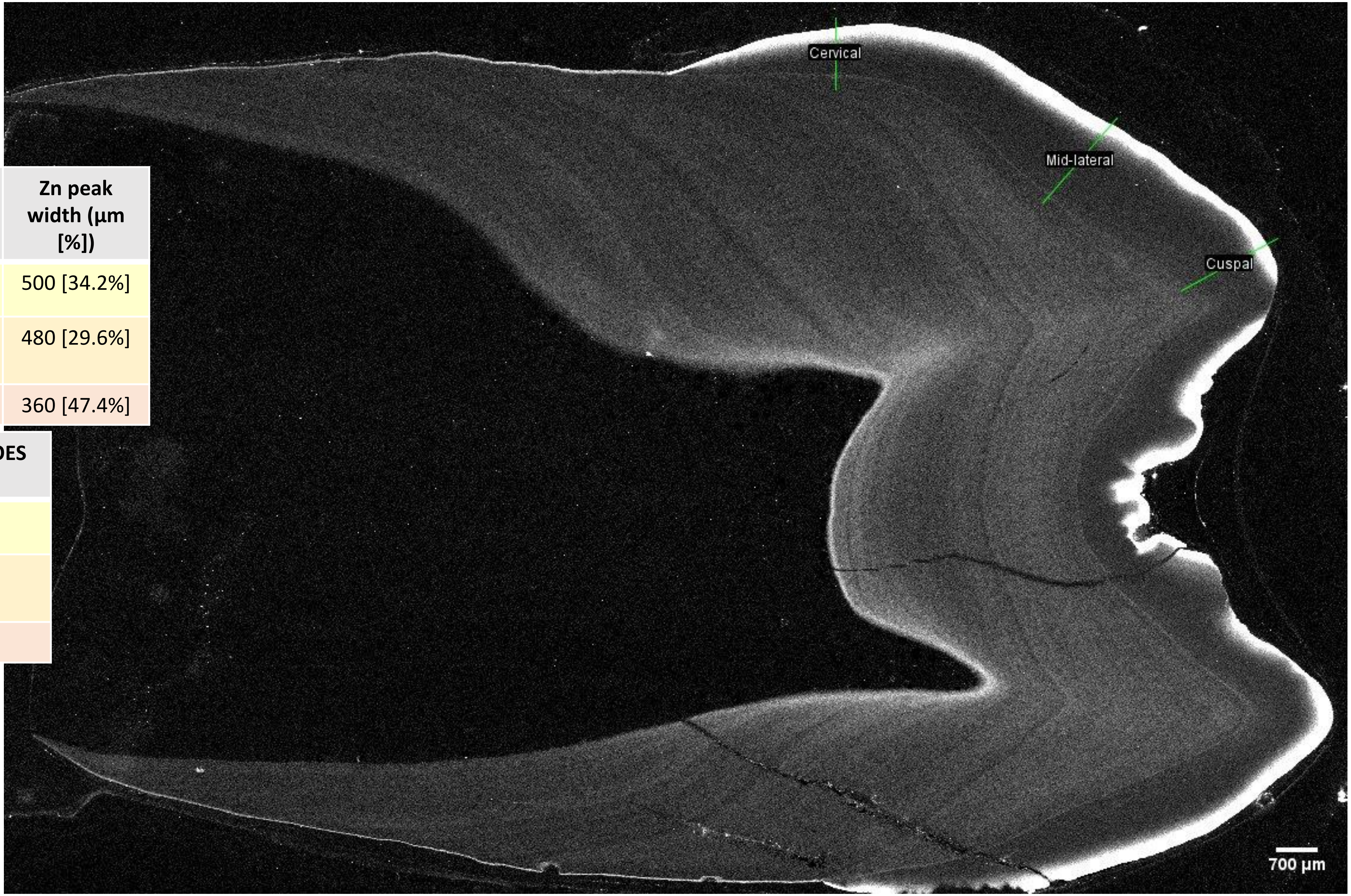

Modern permanent teeth

S29 *Gorilla* CA4 LRM3

Resolution: 20 µm

| | Enamel thickness [µm] | Zn peak (ppm) | Zn peak width (µm [%]) |
|---|---|---|---|
| **Cuspal** | 1740 | 790 | 680 [39.1%] |
| **Mid-lateral** | 1120 | 417 | 580 [51.8%] |
| **Cervical** | 840 | 509 | 460 [54.8%] |

| | "Take-off" to Zn peak (µm [%]) | Zn peak to OES (µm [%]) |
|---|---|---|
| **Cuspal** | 640 [36.8%] | 40 [2.3%] |
| **Mid-lateral** | 380 [33.9%] | 200 [17.9%] |
| **Cervical** | 340 [40.5%] | 120 [14.3%] |

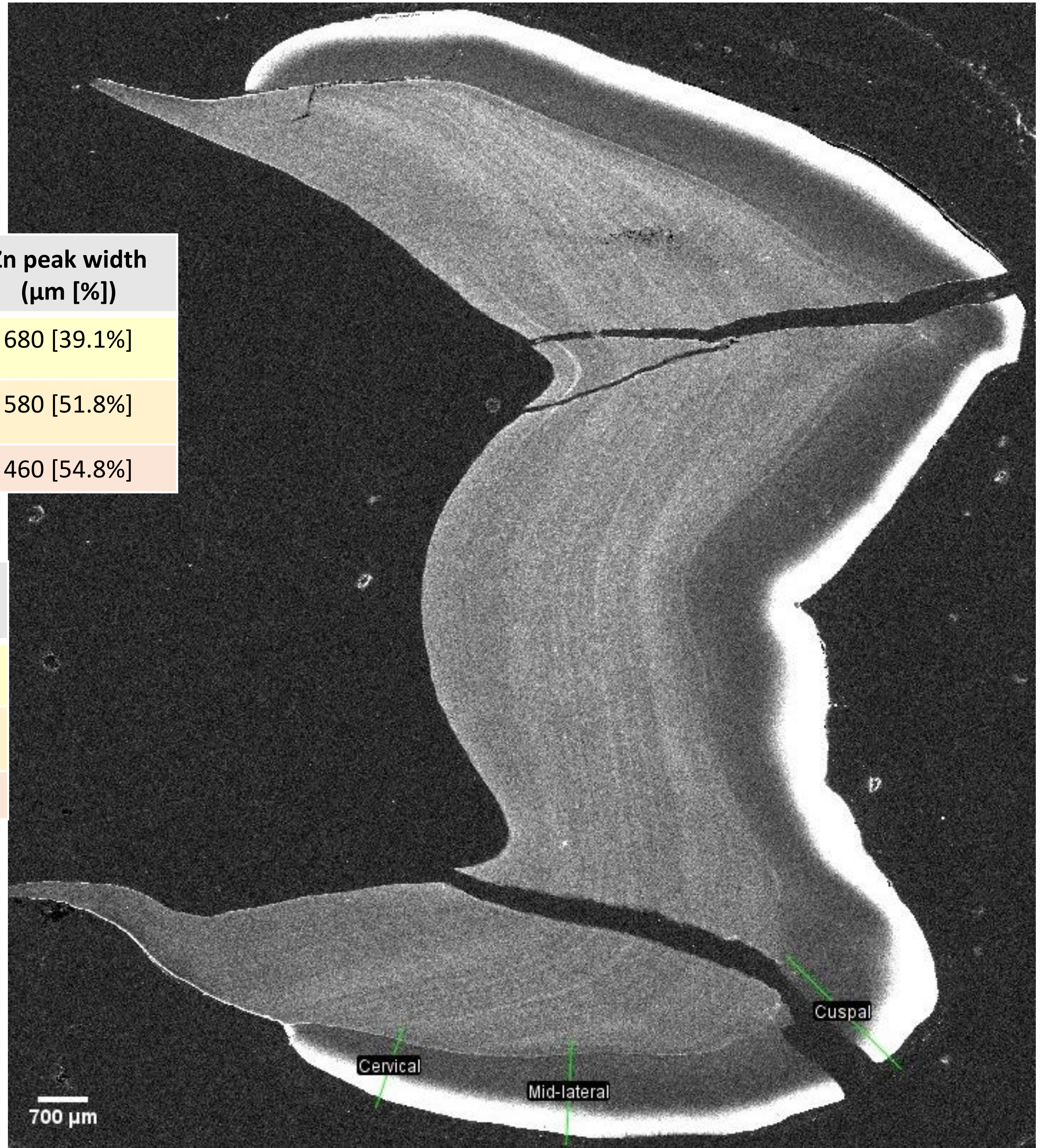

S30 *Pan* CA19B LLM1

Resolution: 20 µm

| | Enamel thickness [µm] | Zn peak (ppm) | Zn peak width (µm [%]) |
|---|---|---|---|
| **Cuspal** | 800 | 622 | 500 [62.5%] |
| **Mid-lateral** | 680 | 400 | 580 [85.3%] |
| **Cervical** | 260 | 556 | 260 [100%] |

| | "Take-off" to Zn peak (µm [%]) | Zn peak to OES (µm [%]) |
|---|---|---|
| **Cuspal** | 440 [55%] | 60 [7.5%] |
| **Mid-lateral** | 540 [79.4%] | 40 [5.9%] |
| **Cervical** | 200 [76.9%] | 60 [23.1%] |

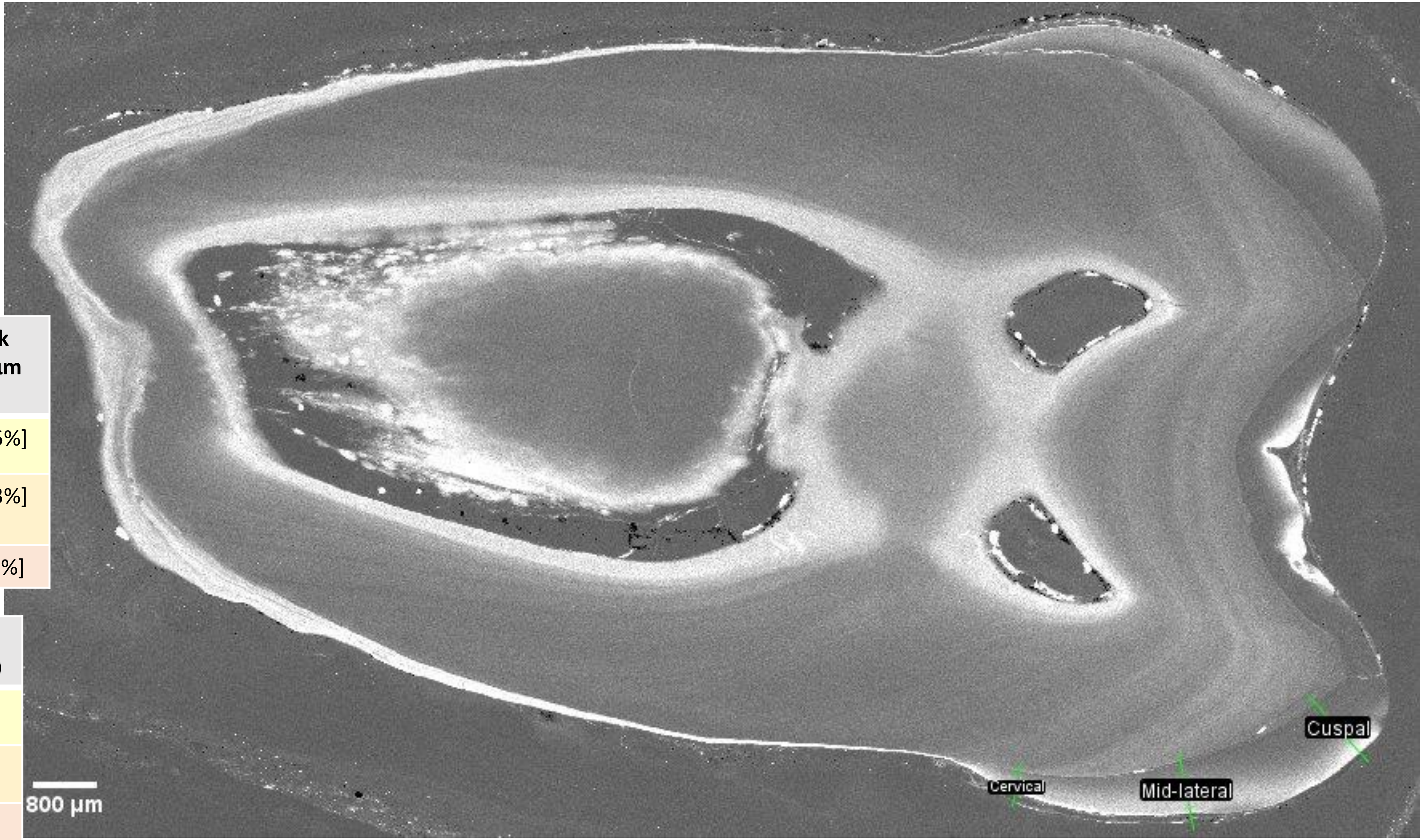

S31 *Pan* CA19B LLM3

Resolution: 20 µm

| | Enamel thickness [µm] | Zn peak (ppm) | Zn peak width (µm [%]) |
|---|---|---|---|
| **Cuspal** | 960 | 752 | 460 [47.9%] |
| **Mid-lateral** | 760 | 696 | 540 [71.1%] |
| **Cervical** | 380 | 776 | 320 [84.2%] |

| | "Take-off" to Zn peak (µm [%]) | Zn peak to OES (µm [%]) |
|---|---|---|
| **Cuspal** | 400 [41.7%] | 60 [6.3%] |
| **Mid-lateral** | 480 [63.2%] | 60 [7.9%] |
| **Cervical** | 300 [78.9%] | 20 [5.3%] |

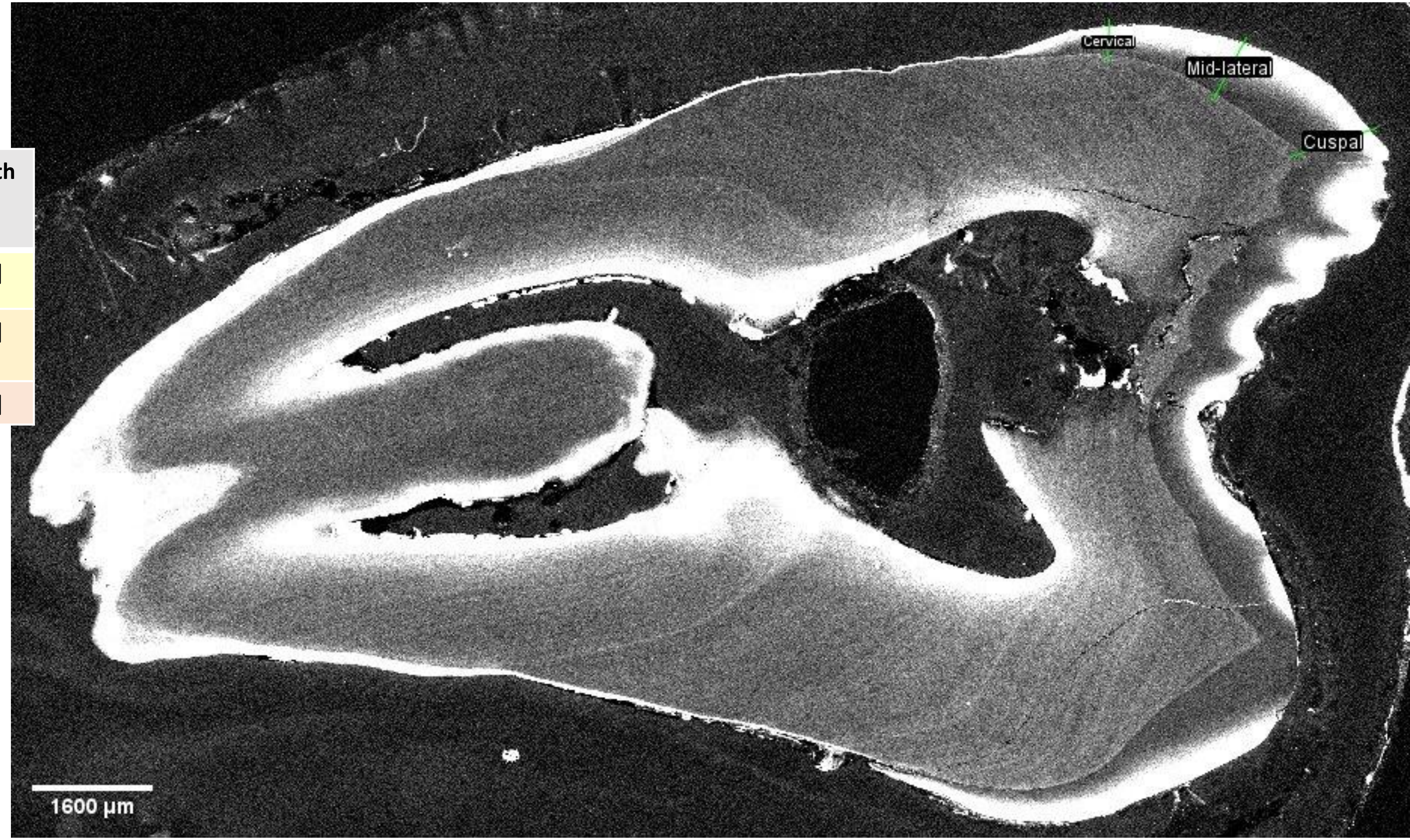

S34 *Pan* CA11D UCL LM3

Resolution: 10 µm

| | Enamel thickness [µm] | Zn peak (ppm) | Zn peak width (µm [%]) |
|---|---|---|---|
| **Cuspal** | 990 | 571 | 560 [56.6%] |
| **Mid-lateral** | 710 | 727 | 500 [70.4%] |
| **Cervical** | 400 | 691 | 360 [90%] |

| | "Take-off" to Zn peak (µm [%]) | Zn peak to OES (µm [%]) |
|---|---|---|
| **Cuspal** | 510 [51.5%] | 50 [5.1%] |
| **Mid-lateral** | 450 [63.4%] | 50 [7%] |
| **Cervical** | 290 [72.5%] | 70 [17.5%] |

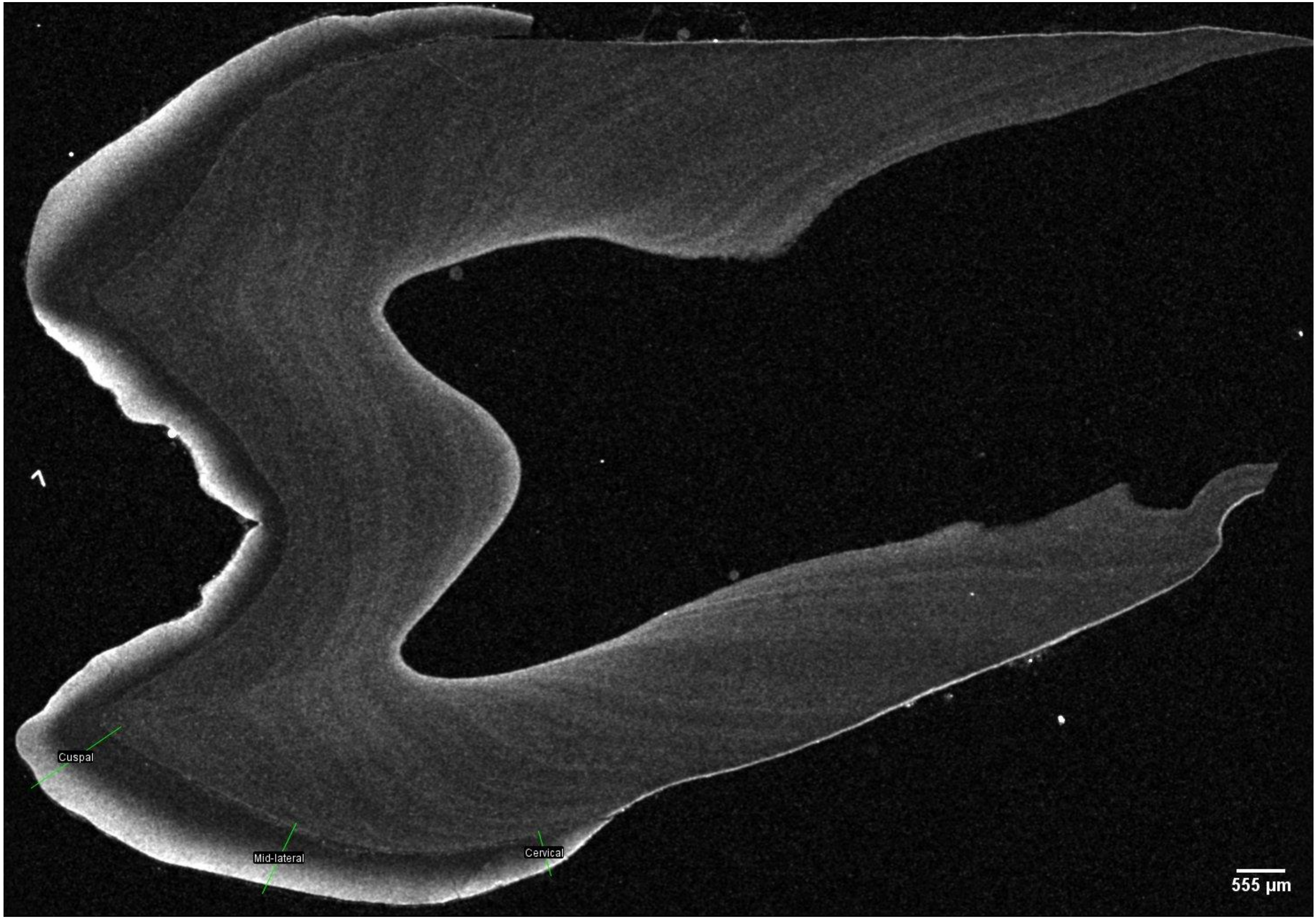

Fossil deciduous teeth

S44 Amud 7 (Neanderthal) Udm1

Resolution: 10 µm

| | Enamel thickness [µm] | Zn peak (ppm) | Zn peak width (µm [%]) |
|---|---|---|---|
| **Cuspal** | 510 | 779 | 150 [29.4%] |
| **Mid-lateral** | 520 | 535 | 210 [40.4%] |
| **Cervical** | 370 | 421 | 160 [43.2%] |

| | "Take-off" to Zn peak (µm [%]) | Zn peak to OES (µm [%]) |
|---|---|---|
| **Cuspal** | 120 [23.5%] | 30 [5.9%] |
| **Mid-lateral** | 180 [34.6%] | 30 [5.8%] |
| **Cervical** | 140 [37.8%] | 20 [5.4%] |

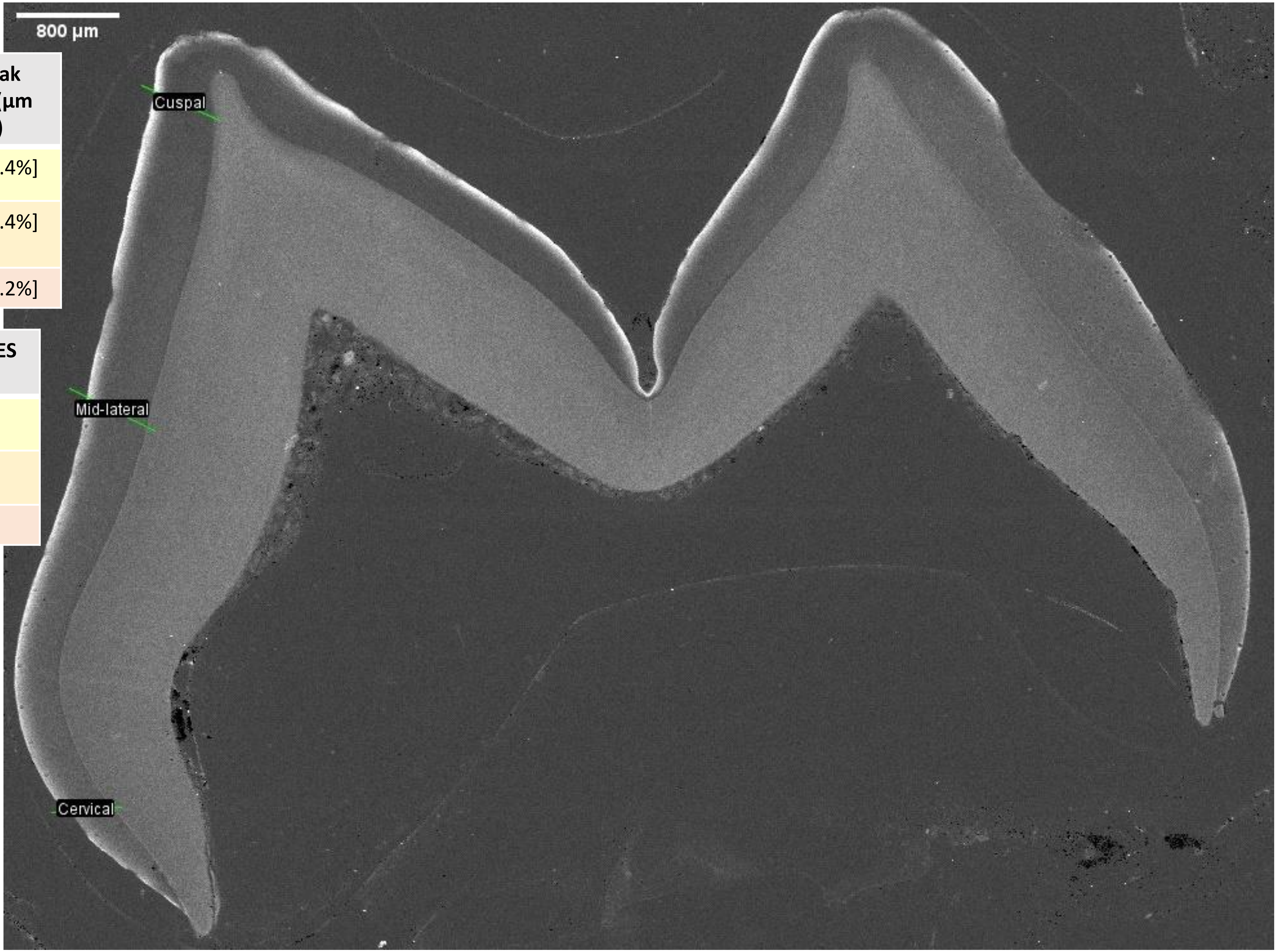

# S89 *Ekembo helesoni* (KNM-RU individual IV) LRdm2 (distal cusp)

Resolution: 10 µm

| | Enamel thickness [µm] | Zn peak (ppm) | Zn peak width (µm [%]) |
|---|---|---|---|
| **Cuspal** | 450 | 1192 | 190 [42.2%] |
| **Mid-lateral** | 290 | 1147 | 170 [58.6%] |
| **Cervical** | 170 | 744 | 150 [88.2%] |

| | "Take-off" to Zn peak (µm [%]) | Zn peak to OES (µm [%]) |
|---|---|---|
| **Cuspal** | 140 [31.1%] | 50 [11.1%] |
| **Mid-lateral** | 120 [41.4%] | 50 [17.2%] |
| **Cervical** | 110 [64.7%] | 40 [23.5%] |

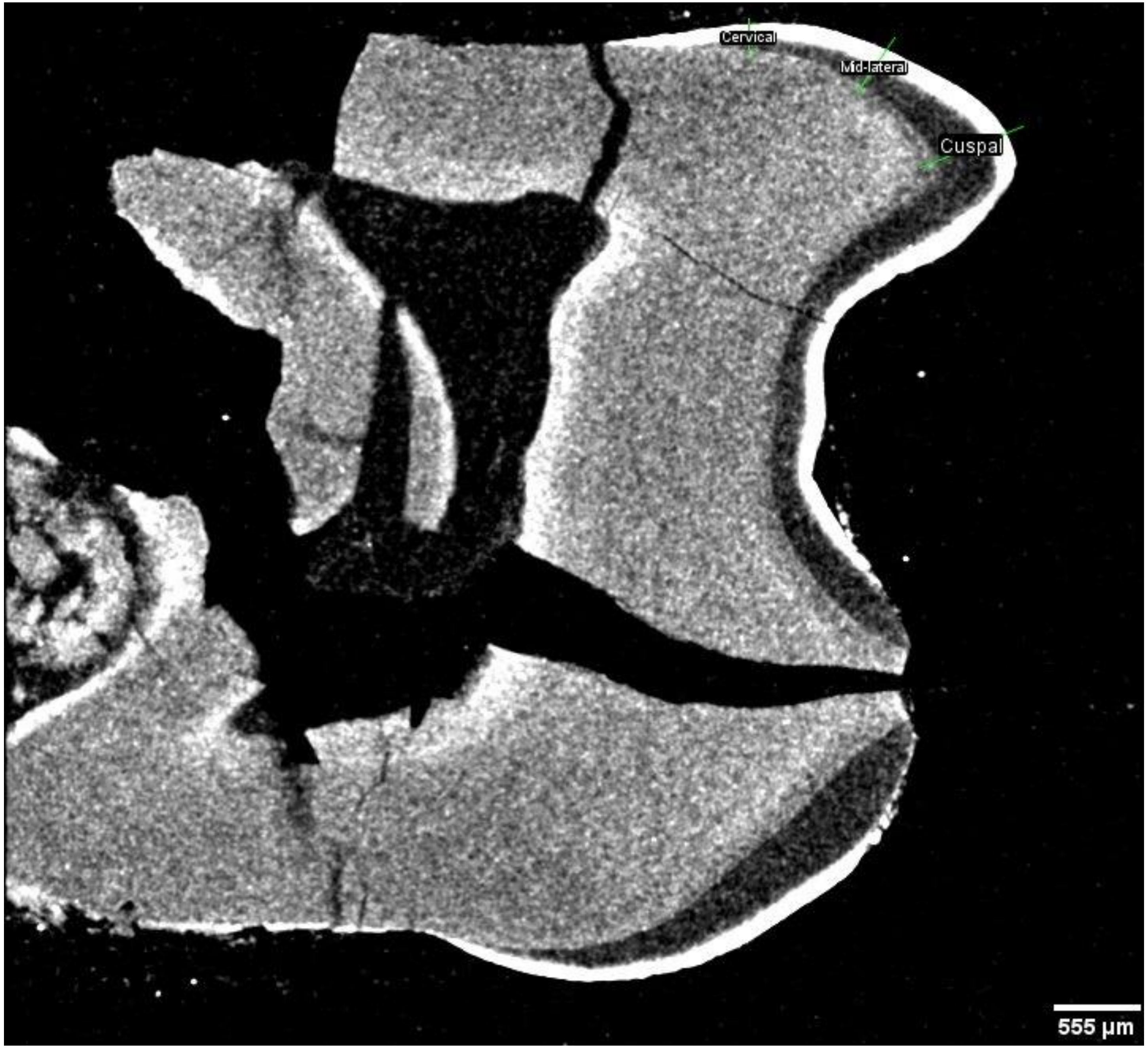

*Ekembo nyanzae* KNM-RU 1721 URM1

Resolution: 15 µm

| | Enamel thickness [µm] | Zn peak (ppm) | Zn peak width (µm [%]) |
|---|---|---|---|
| **Cuspal** | 1755 | 885 | 645 [36.8%] |
| **Mid-lateral** | 1395 | 667 | 600 [43.0%] |
| **Cervical** | 570 | 536 | 480 [84.2%] |

| | “Take-off” to Zn peak (µm [%]) | Zn peak to OES (µm [%]) |
|---|---|---|
| **Cuspal** | 585 [33.3%] | 60 [3.4%] |
| **Mid-lateral** | 555 [39.8%] | 45 [3.2%] |
| **Cervical** | 420 [73.7%] | 60 [10.5%] |

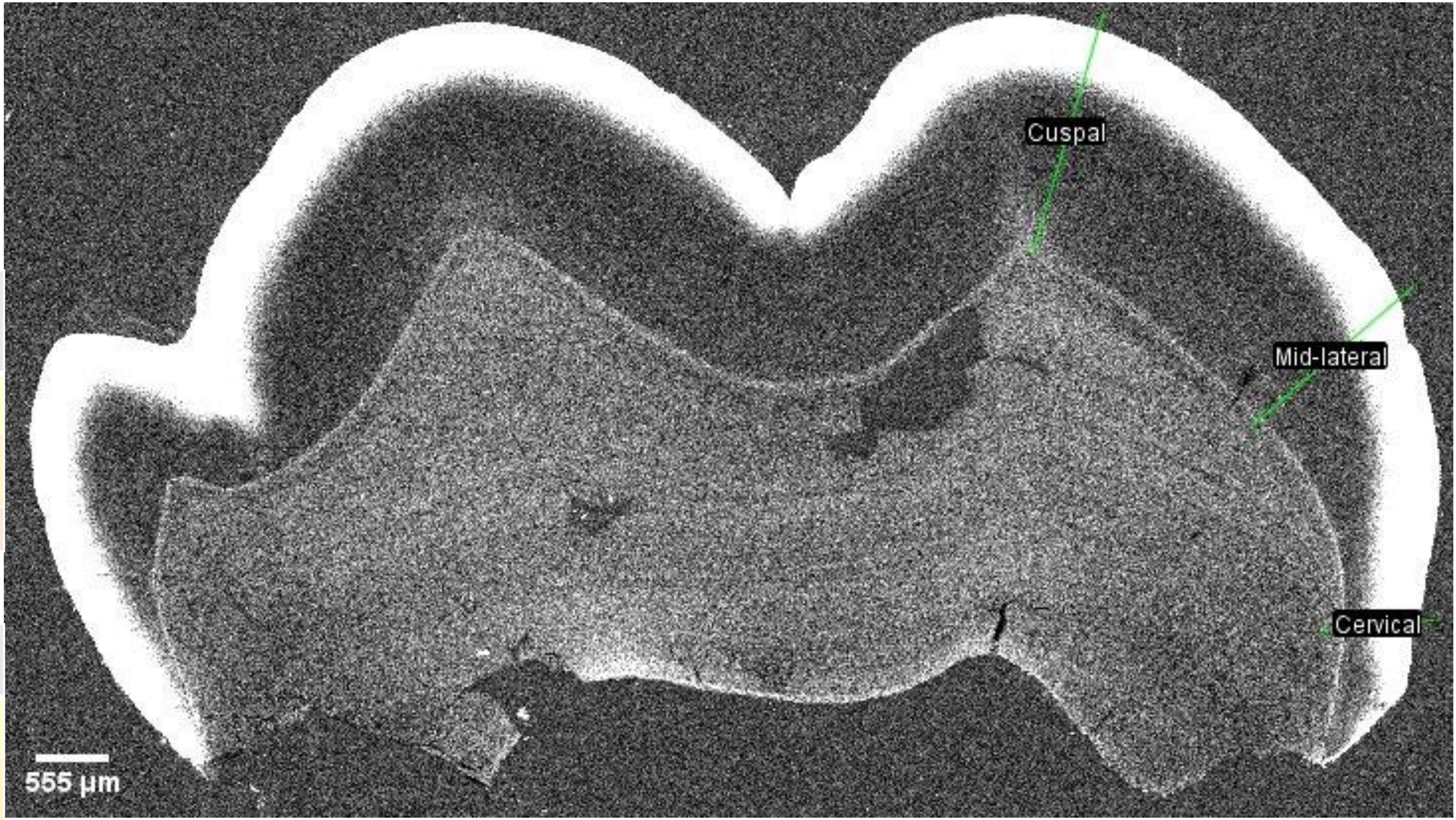

# S90 *Ekembo heseloni* (KNM-RU individual IV) LLM1

Resolution: 10 µm

| | Enamel thickness [µm] | Zn peak (ppm) | Zn peak width (µm [%]) |
|---|---|---|---|
| **Cuspal** | 660 | 1241 | 260 [39.4%] |
| **Mid-lateral** | 860 | 1253 | 380 [44.2%] |
| **Cervical** | 280 | 706 | 220 [89.3%] |

| | "Take-off" to Zn peak (µm [%]) | Zn peak to OES (µm [%]) |
|---|---|---|
| **Cuspal** | 220 [33.3%] | 40 [6.1%] |
| **Mid-lateral** | 320 [37.2%] | 60 [7.0%] |
| **Cervical** | 200 [71.4%] | 50 [17.9%] |

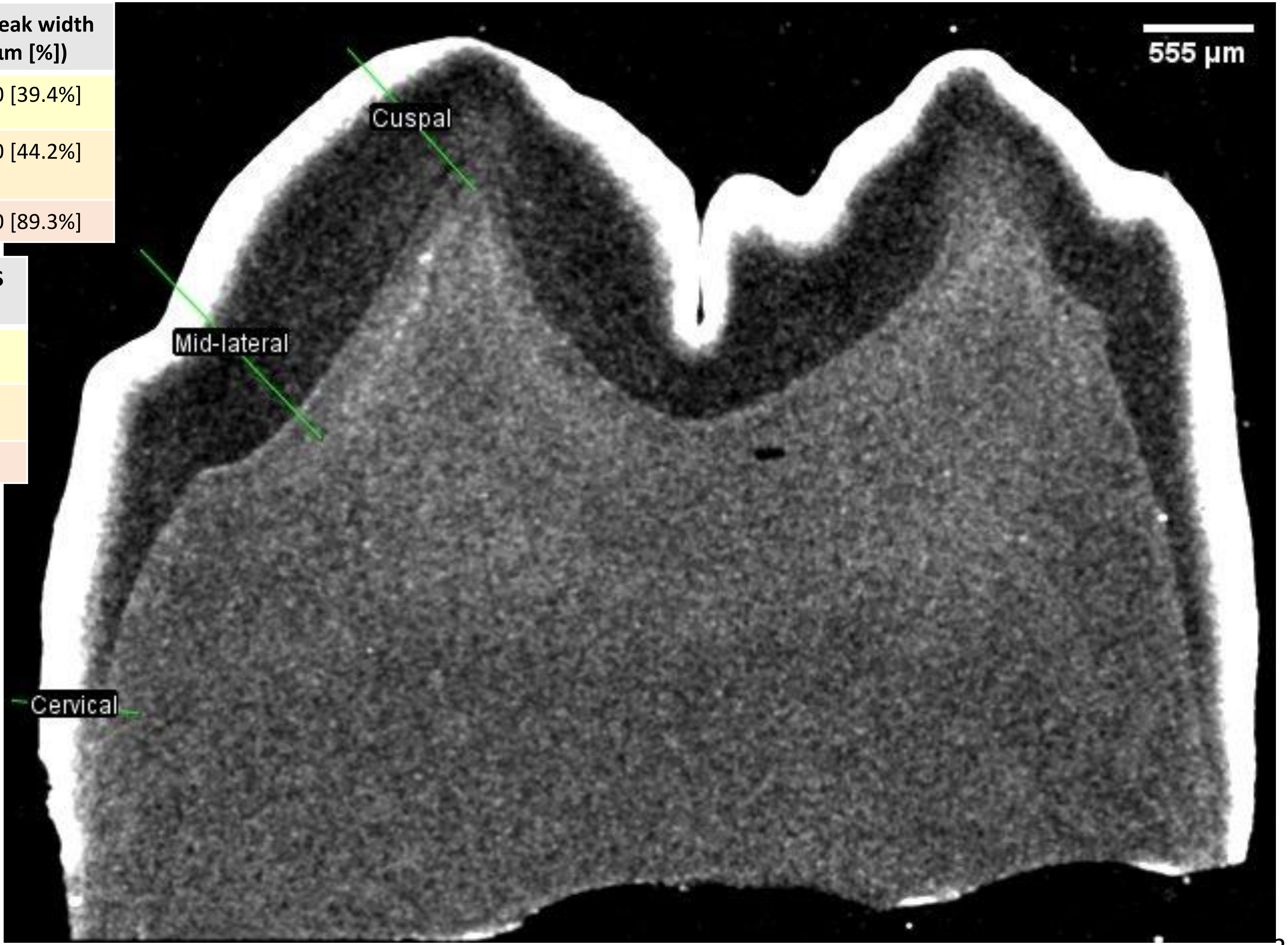

Fossil permanent teeth

# S46 *Hispanopithecus laietanus* IPS 1781 UM1

Resolution: 15 µm

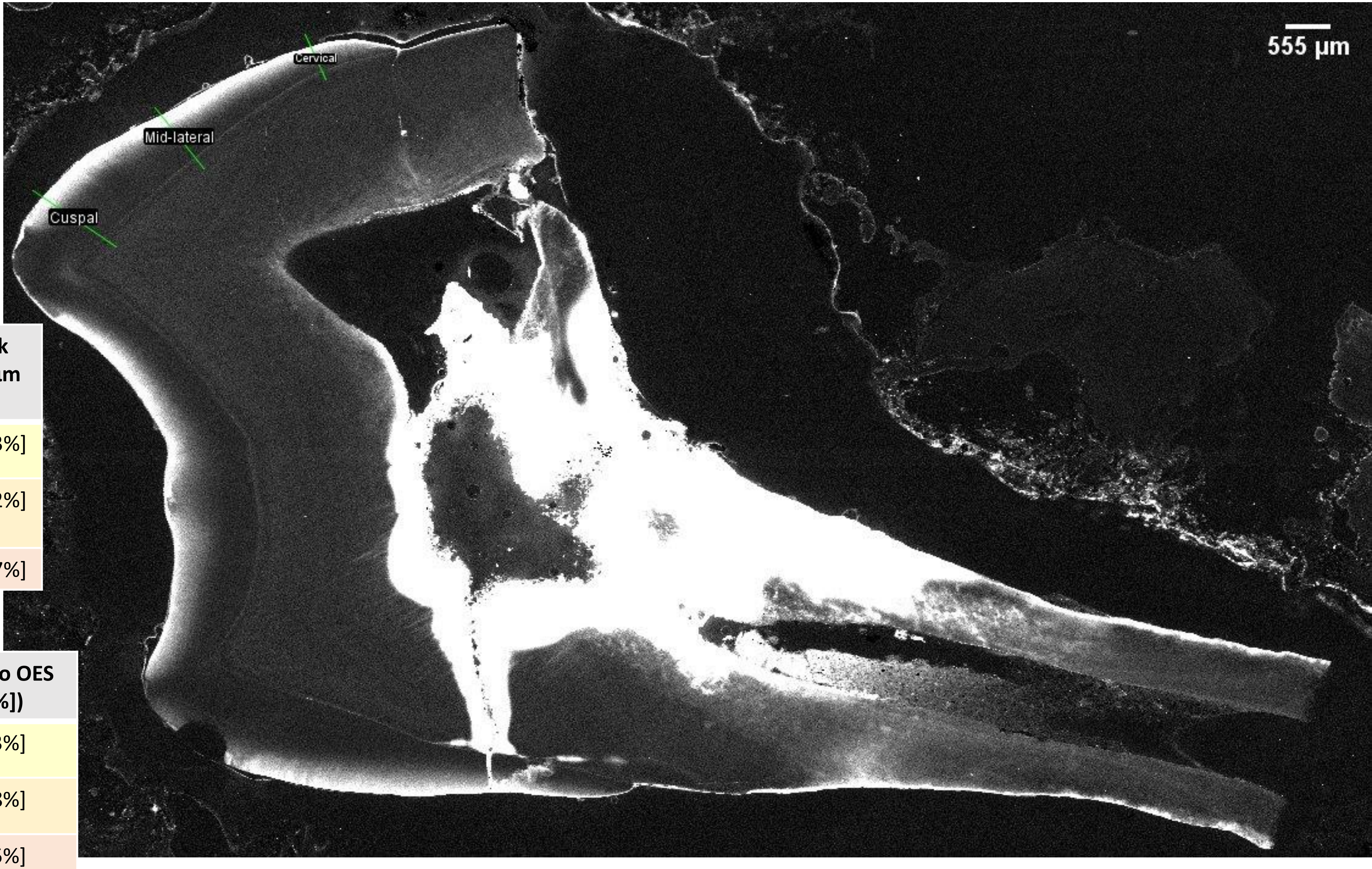


| | Enamel thickness [µm] | Zn peak (ppm) | Zn peak width (µm [%]) |
|---|---|---|---|
| **Cuspal** | 900 | 971 | 525 [58.3%] |
| **Mid-lateral** | 660 | 995 | 450 [68.2%] |
| **Cervical** | 315 | 739 | 270 [85.7%] |

| | "Take-off" to Zn peak (µm [%]) | Zn peak to OES (µm [%]) |
|---|---|---|
| **Cuspal** | 495 [55%] | 30 [3.3%] |
| **Mid-lateral** | 405 [61.4%] | 45 [6.8%] |
| **Cervical** | 240 [76.2%] | 30 [9.5%] |

Fossil permanent teeth

# S205 KNM-ER 30748 *Au. anamensis* LP4

Resolution: 10 µm

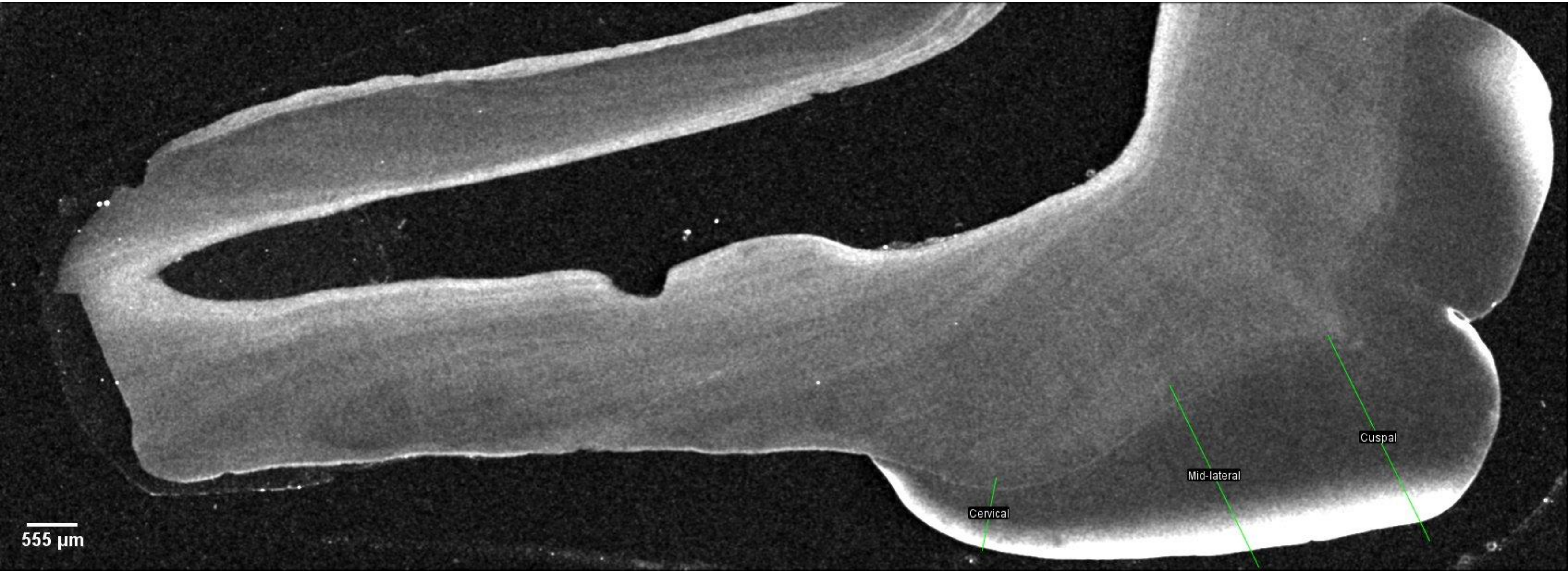


| | Enamel thickness [µm] | Zn peak (ppm) | Zn peak width (µm [%]) | "Take-off" to Zn peak (µm [%]) | Zn peak to OES (µm [%]) |
|---|---|---|---|---|---|
| **Cuspal** | 2150 | 544 | 750 [34.9%] | 670 [31.2%] | 80 [3.7%] |
| **Mid-lateral** | 1770 | 506 | 720 [40.7%] | 650 [36.7%] | 70 [4%] |
| **Cervical** | 650 | 393 | 330 [50.8%] | 280 [43.1%] | 50 [7.7%] |

## S40 SK 835 *P. robustus* UM3 (Buccal)

Resolution: 10 µm

| | Enamel thickness [µm] | Zn peak (ppm) | Zn peak width (µm [%]) |
|---|---|---|---|
| **Cuspal** | 2820 | 879 | 950 [33.7%] |
| **Mid-lateral** | 1970 | 1000 | 950 [48.2%] |
| **Cervical** | 690 | 1506 | 560 [81.2%] |

| | "Take-off" to Zn peak (µm [%]) | Zn peak to OES (µm [%]) |
|---|---|---|
| **Cuspal** | 930 [32.6%] | 30 [1.1%] |
| **Mid-lateral** | 900 [45.7%] | 50 [2.5%] |
| **Cervical** | 540 [78.3%] | 20 [2.9%] |

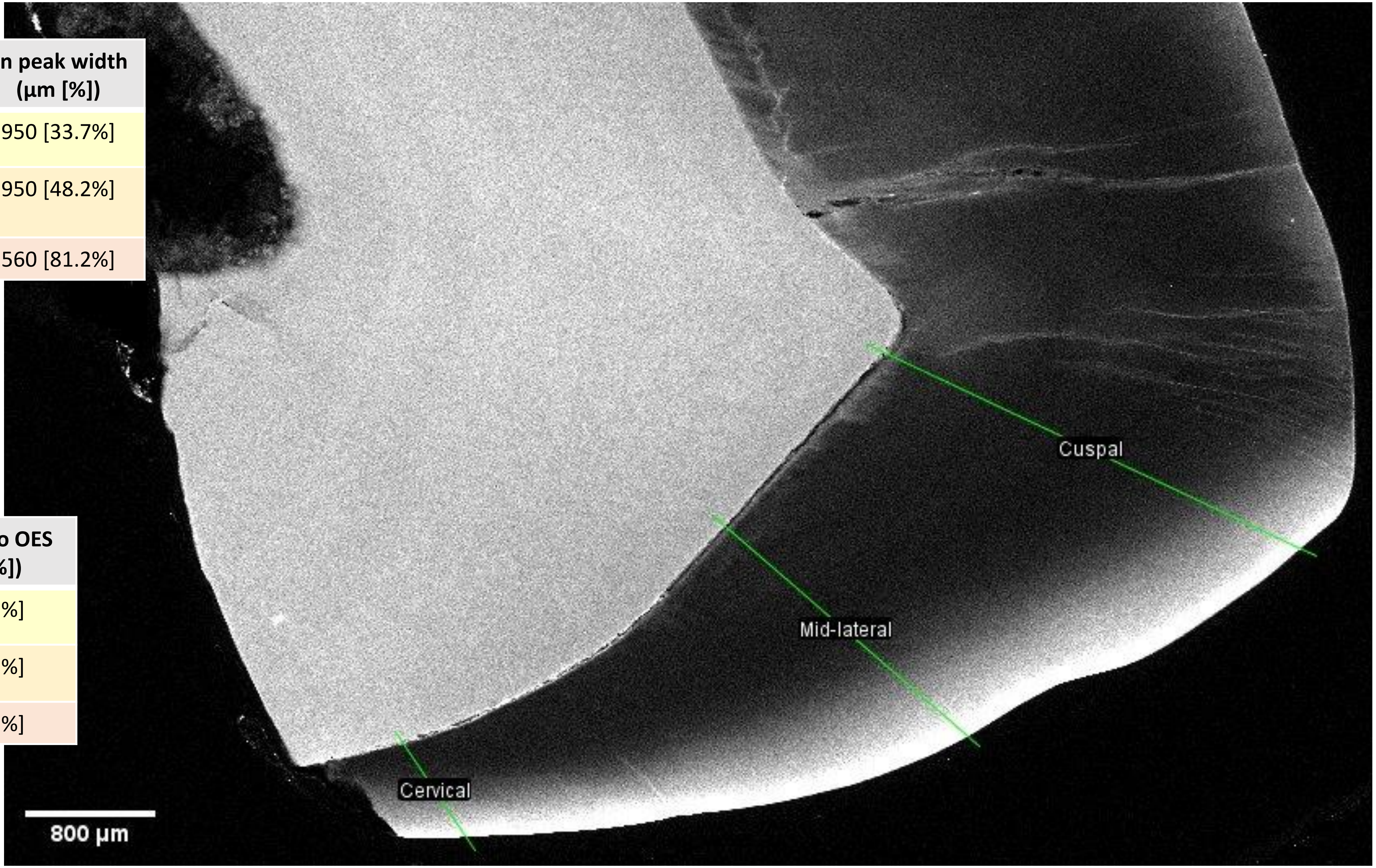

# S45 Skhul IV (early *H. sapiens*) LLM3

Resolution: 15 µm

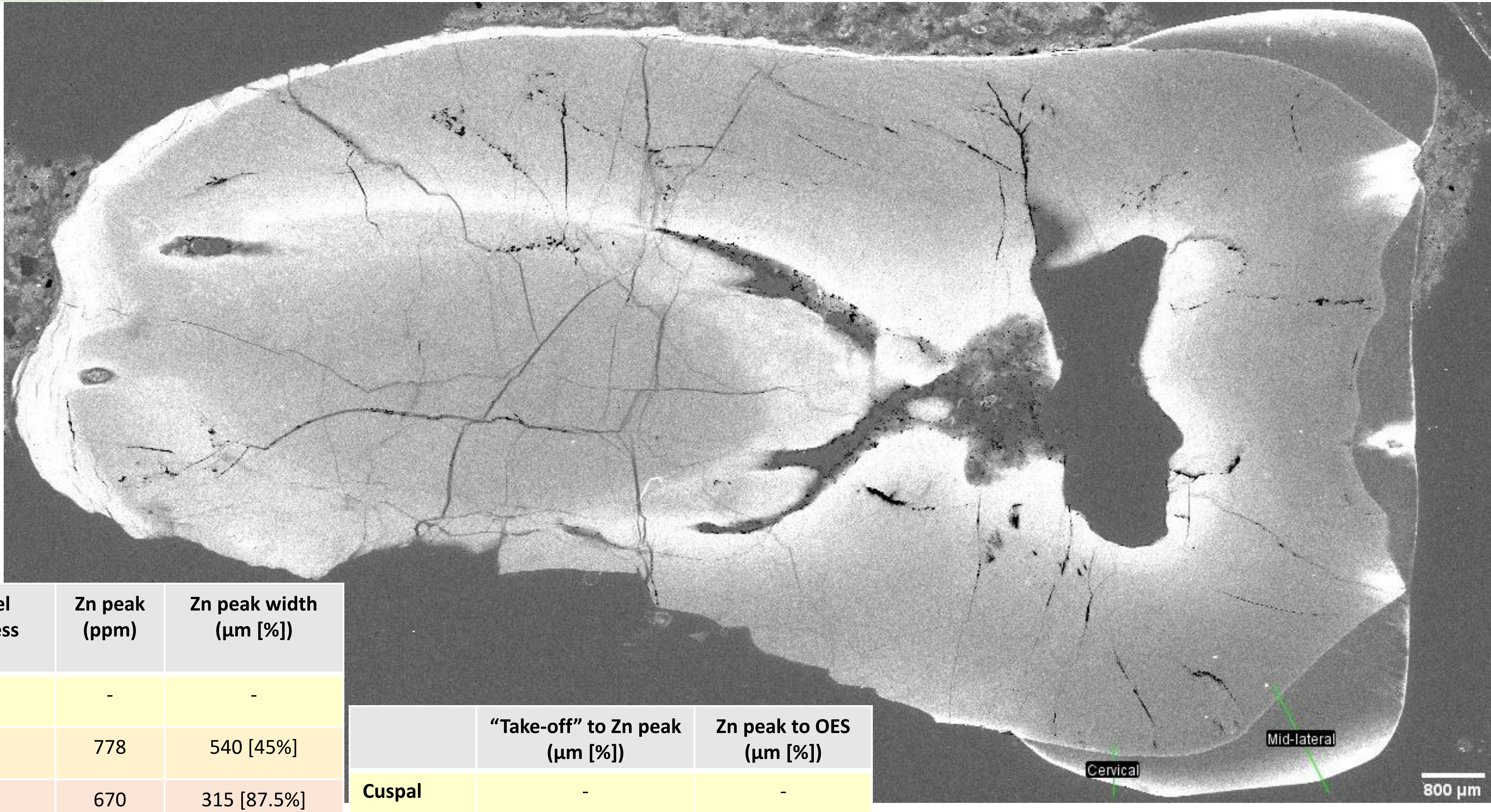


| | Enamel thickness [µm] | Zn peak (ppm) | Zn peak width (µm [%]) |
|---|---|---|---|
| **Cuspal** | Worn | - | - |
| **Mid-lateral** | 1200 | 778 | 540 [45%] |
| **Cervical** | 360 | 670 | 315 [87.5%] |

| | "Take-off" to Zn peak (µm [%]) | Zn peak to OES (µm [%]) |
|---|---|---|
| **Cuspal** | - | - |
| **Mid-lateral** | 495 [41.3%] | 45 [3.8%] |
| **Cervical** | 285 [79.2%] | 30 [8.3%] |
