# Supplementary material for: The Distribution and Biogenic Origins of Zinc in the Mineralised Tooth Tissues of Modern and Fossil Hominoids: Implications for Life History, Diet and Taphonomy
Source: Biology (Basel). 2023 Nov 21;12(12):1455. doi: 10.3390/biology12121455 (PMC10740576; doi:10.3390/biology12121455)
Supplement: Supplementary file 1 [file biology-12-01455-s001.zip › Dean_et_al_2023_Biology_Appendix SB.pdf]

The Distribution and Biogenic Origins of Zinc in the Mineralised Tooth Tissues of Modern and Fossil Hominoids: Implications for Life History, Diet and Taphonomy

Appendix SB: Statistical methods and results for the correlation among variables

M. Christopher Dean, Jan Garrevoet, Stijn J. M. Van Malderen, Frédéric Santos, Marta Mirazón Lahr, Robert Foley and Adeline Le Cabec

October 29, 2023

Contents

1 Introduction **2**

2 Load data **3**

3 Correlation matrices **5**
3.1 All teeth 5
3.2 Deciduous teeth 8
3.3 Permanent teeth 11

4 Scatterplot matrices **14**
4.1 All teeth 14
4.2 Deciduous teeth 17
4.3 Permanent teeth 20

5 Principal component analyses **23**
5.1 By region 23
5.2 By tooth type 25

6 Formal testing of statistical hypotheses **26**
6.1 Maximal Zn concentration by tooth type 26
6.2 Maximal Zn concentration by enamel region 27
6.2.1 Including all teeth 27
6.2.2 Interaction between enamel region and tooth type 28
6.3 Comparisons of correlation coefficients 29

1. Introduction

This document provides additional results that are not presented in the main text of our article. All the analyses were performed using R (R Core Team, 2023), and this document has been built with Org mode 9.6.10 for GNU Emacs 29.1 (Schulte, Davison, Dye, & Dominik, 2012).

Along with R version 4.3.1 (2023-06-16) itself, the following R packages are loaded, using their version available on CRAN at a fixed date (2023-09-22), using the R package {groundhog} (Simonsohn & Gruson, 2021):

```r
## Use groundhog to improve the reproducibility:
library(groundhog)
gday <- "2023-09-22"
## Load the following packages:
groundhog.library("car", date = gday)
groundhog.library("cocor", date = gday)
groundhog.library("corrplot", date = gday)
groundhog.library("cowplot", date = gday)
groundhog.library("dplyr", date = gday)
groundhog.library("ez", date = gday)
groundhog.library("FactoMineR", date = gday)
groundhog.library("factoextra", date = gday)
groundhog.library("GGally", date = gday)
groundhog.library("ggbeeswarm", date = gday)
groundhog.library("ggpubr", date = gday)
groundhog.library("janitor", date = gday)
groundhog.library("lme4", date = gday)
groundhog.library("MASS", date = gday)
groundhog.library("psych", date = gday)
groundhog.library("textshape", date = gday)
```

Additional details about the R session:

```r
print(sessionInfo(), locale = FALSE)
```

```
R version 4.3.1 (2023-06-16)
Platform: x86_64-pc-linux-gnu (64-bit)
Running under: Manjaro Linux

Matrix products: default
BLAS:   /usr/lib/libblas.so.3.11.0
LAPACK: /usr/lib/liblapack.so.3.11.0

attached base packages:
[1] stats     graphics  grDevices utils     datasets  methods   base

other attached packages:
 [1] textshape_1.7.3  psych_2.3.6      MASS_7.3-60      lme4_1.1-34
 [5] Matrix_1.6-1.1   janitor_2.2.0    ggpubr_0.6.0     ggbeeswarm_0.7.2
```

```
 [9] GGally_2.1.2     factoextra_1.0.7 ggplot2_3.4.3    FactoMineR_2.8
[13] ez_4.4-0         dplyr_1.1.3      cowplot_1.1.1    corrplot_0.92
[17] cocor_1.1-4      car_3.1-2        carData_3.0-5    groundhog_3.1.1
```

For all technical questions about this document or the R scripts included hereafter, or for any issue in reproducing the results, feel free to send an email to frederic.santos@u-bordeaux.fr. All the R code written to produce this document can be found online in a GitLab repository (`https://gitlab.com/f-santos/dean-et-al-2023-supporting-files`).

2. Load data

The data file, in CSV format, can be found on Zenodo (Dean et al., 2023).

Here is a short summary of the data file:

```r
summary(dat, maxsum = 11)
```

```
             specimen_id                        taxon    fossil_modern type
 KNM-RU 1721        : 3   Au. anamensis           : 3   f:24          d:39
 S16 UCL CA28JS7    : 3   early H. sapiens        : 3   m:54          p:39
 S18 UCL CA28JS7 JS6: 3   Ekembo helesoni         : 6
 S19 UCL CA1F1472   : 3   Ekembo nyanzae          : 3
 S205 KNM-ER 30748  : 3   Gorilla                 :12
 S209               : 3   Hispanopithecus laietanus: 3
 S22 UCL CA11       : 3   Modern H. sapiens       :24
 S27 UCL CA4        : 3   Neanderthal             : 3
 S28 CA4            : 3   P. robustus             : 3
 S29 CA4            : 3   Pan                     :12
 (Other)            :48   Pongo                   : 6
     tooth     enamel_region enamel_thickness zn_peak_max_conc zn_peak_width
 Udm2   :10   cu:26          Min.   :  14.0   Min.   : 197.0   Min.   : 13.0
 LRdm2  : 9   m :26          1st Qu.: 330.0   1st Qu.: 562.0   1st Qu.:240.0
 LLM1   : 6   ce:26          Median : 660.0   Median : 790.0   Median :330.0
 M3     : 6                  Mean   : 710.6   Mean   : 871.2   Mean   :357.5
 dC     : 3                  3rd Qu.: 840.0   3rd Qu.:1120.0   3rd Qu.:480.0
 dm1    : 3                  Max.   :2820.0   Max.   :1743.0   Max.   :950.0
 dM1    : 3                  NA's   :1        NA's   :1        NA's   :1
 LdC    : 3
 LLM3   : 3
 LM1    : 3
 (Other):29
  zn_peak_oes     take_off_to_peak perc_peak_width_to_eth perc_take_off_to_eth
 Min.   :  1.00   Min.   :  9.0    Min.   : 27.00         Min.   :23.00
 1st Qu.: 30.00   1st Qu.:200.0    1st Qu.: 42.20         1st Qu.:36.70
 Median : 40.00   Median :300.0    Median : 52.00         Median :43.60
 Mean   : 42.76   Mean   :314.8    Mean   : 57.98         Mean   :49.73
 3rd Qu.: 50.00   3rd Qu.:420.0    3rd Qu.: 77.80         3rd Qu.:63.40
 Max.   :200.00   Max.   :920.0    Max.   :100.00         Max.   :90.90
 NA's   :1        NA's   :1        NA's   :1              NA's   :1
```

```
perc_oes_peak_to_eth
Min.   : 1.100
1st Qu.: 4.100
Median : 6.300
Mean   : 8.245
3rd Qu.:10.000
Max.   :33.300
NA's   :1
```

with the following abbreviations for the variables names:

- `type`: type of tooth (`d` for deciduous, `p` for permanent);
- `enamel_region`: region of enamel (`cu` for cuspal, `m` for midlateral, `ce` for cervical) in which the measurement was performed;
- `enamel_thickness`: linear measurement of the total enamel thickness from the EDJ (enamel dentine junction) to the OES (outer enamel surface) (in µm);
- `zn_peak_max_conc`: maximal value of Zn concentration (in ppm);
- `zn_peak_width`: total width of the Zn-enriched band of enamel (in µm);
- `zn_peak_oes`: distance between the OES and the location in enamel where the peak in Zn concentration occurs, close to the enamel sub-surface but still within the Zn-enriched enamel band (in µm);
- `take_off_to_peak`: distance within the Zn-enriched enamel band between the location in enamel where the peak in Zn concentration occurs and the take-off of that curve deeper in enamel (in µm);
- `perc_peak_width_to_eth`: proportion of the total width of the Zn-enriched enamel band (`zn_peak_width`) to the total (linear) enamel thickness (in %);
- `perc_take_off_to_eth`: proportion of the distance between the take-off of the curve representing the Zn-enriched enamel band and the peak in Zn concentration (`take_off_to_peak`) to the total (linear) enamel thickness (in %);
- `perc_oes_peak_to_eth`: proportion of the distance between the OES and the peak in Zn concentration (`zn_peak_oes`) to the total (linear) enamel thickness (in %).

3. Correlation matrices

Various correlation matrices are computed and displayed in Figures 1 to 9, for each enamel region and each type of teeth. Since there are some strong outliers in the data, the values displayed in these figures are Spearman correlation coefficients, in order to increase the robustness of the correlations measured.

Above the diagonal, positive correlations are pictured in blue, while negative correlations are coloured in red; the larger the square the stronger the correlation. Below the diagonal, the actual Spearman r_s values are displayed following the same color scheme. Non-significant correlations (at the 0.05 level) are indicated by a $\times$.

3.1. All teeth

Figures 1 to 3 were computed using the whole set of teeth available.

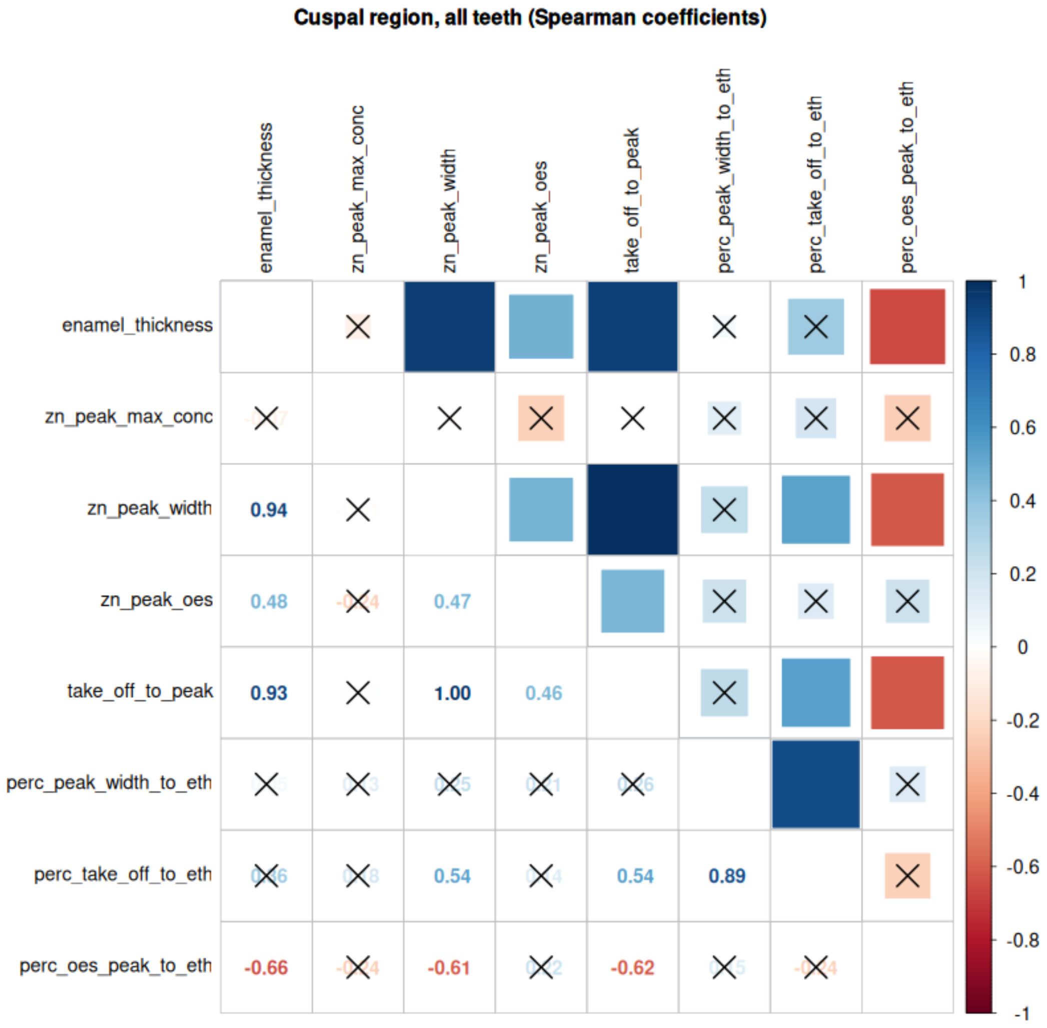

Figure 1: Graphical representation of the correlation matrix among variables for the whole set of teeth, measured in the cuspal region.

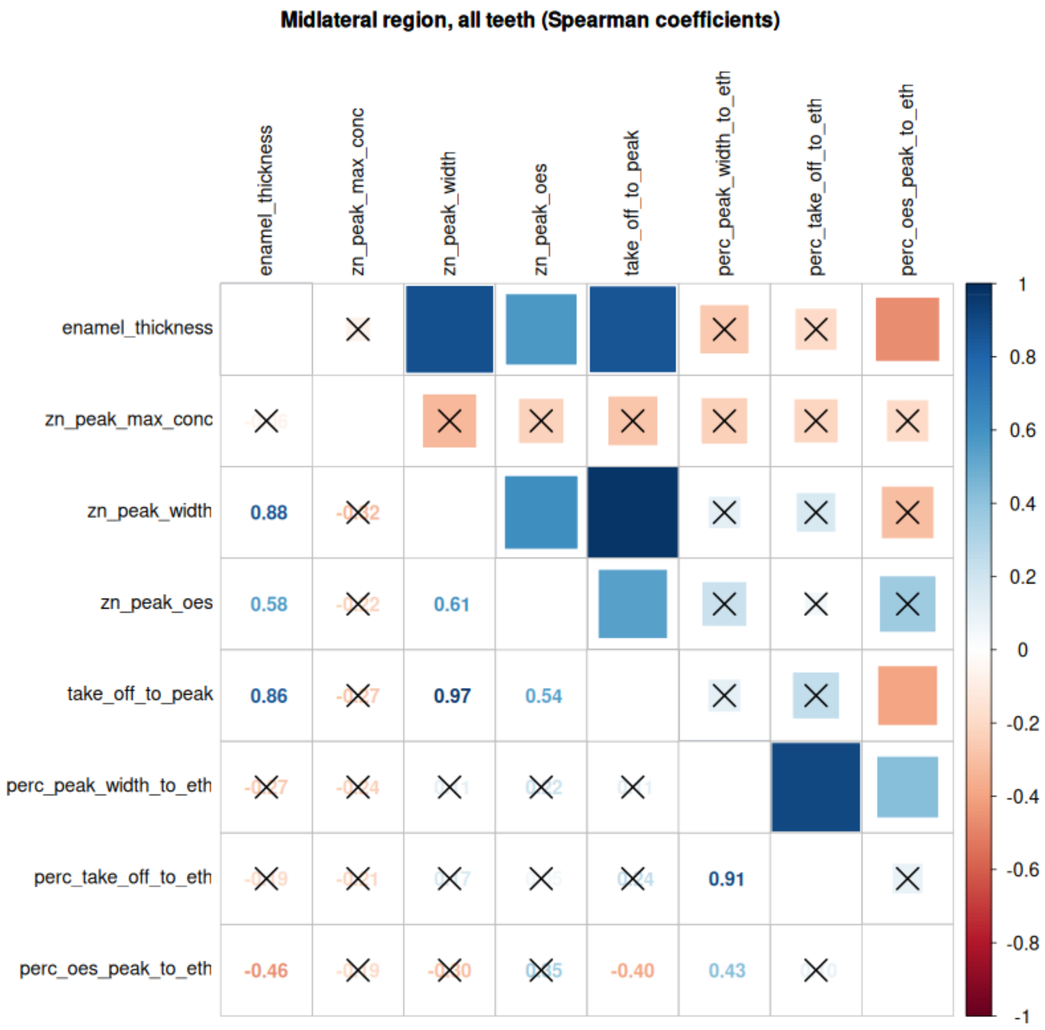

Figure 2: Graphical representation of the correlation matrix among variables for the whole set of teeth, measured in the midlateral region.

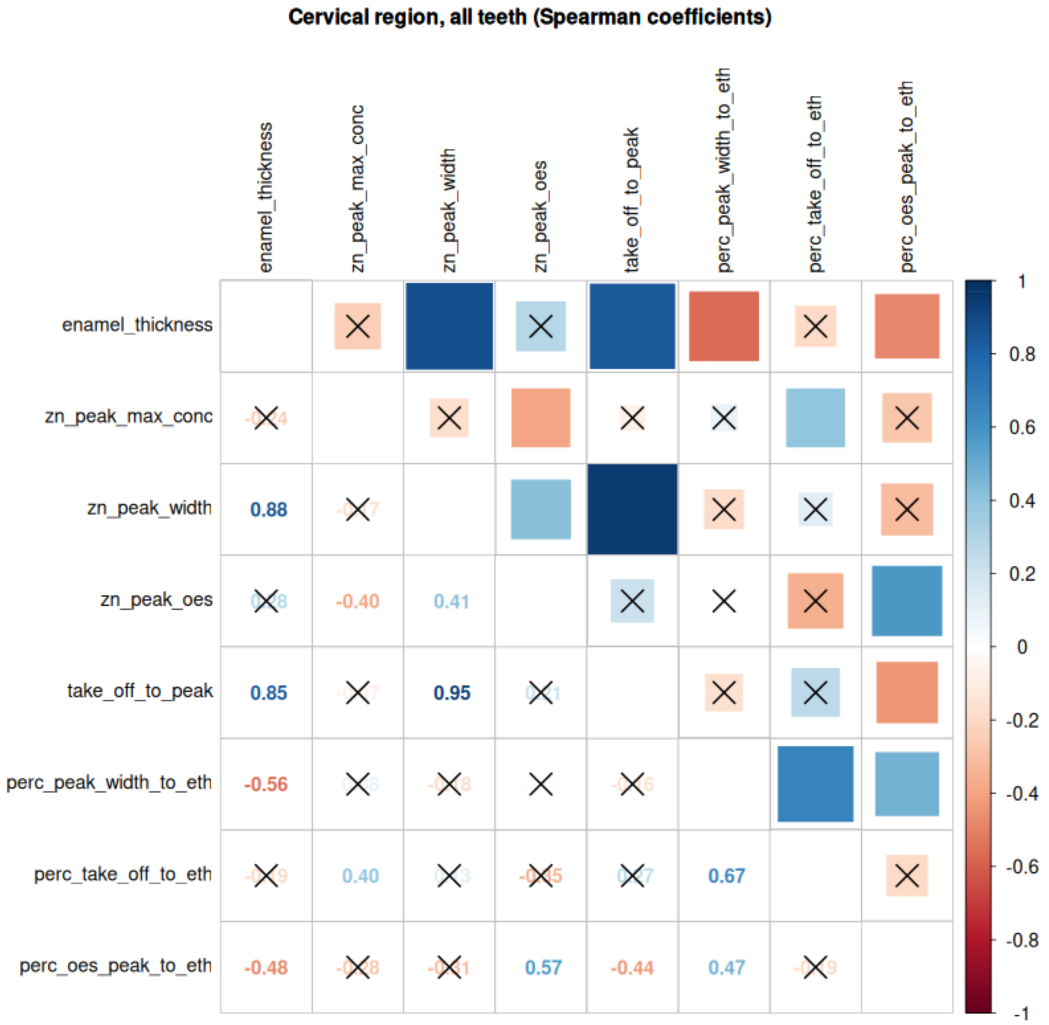

Figure 3: Graphical representation of the correlation matrix among variables for the whole set of teeth, measured in the cervical region.

3.2. Deciduous teeth

Figures 4 to 6 were computed using only deciduous teeth.

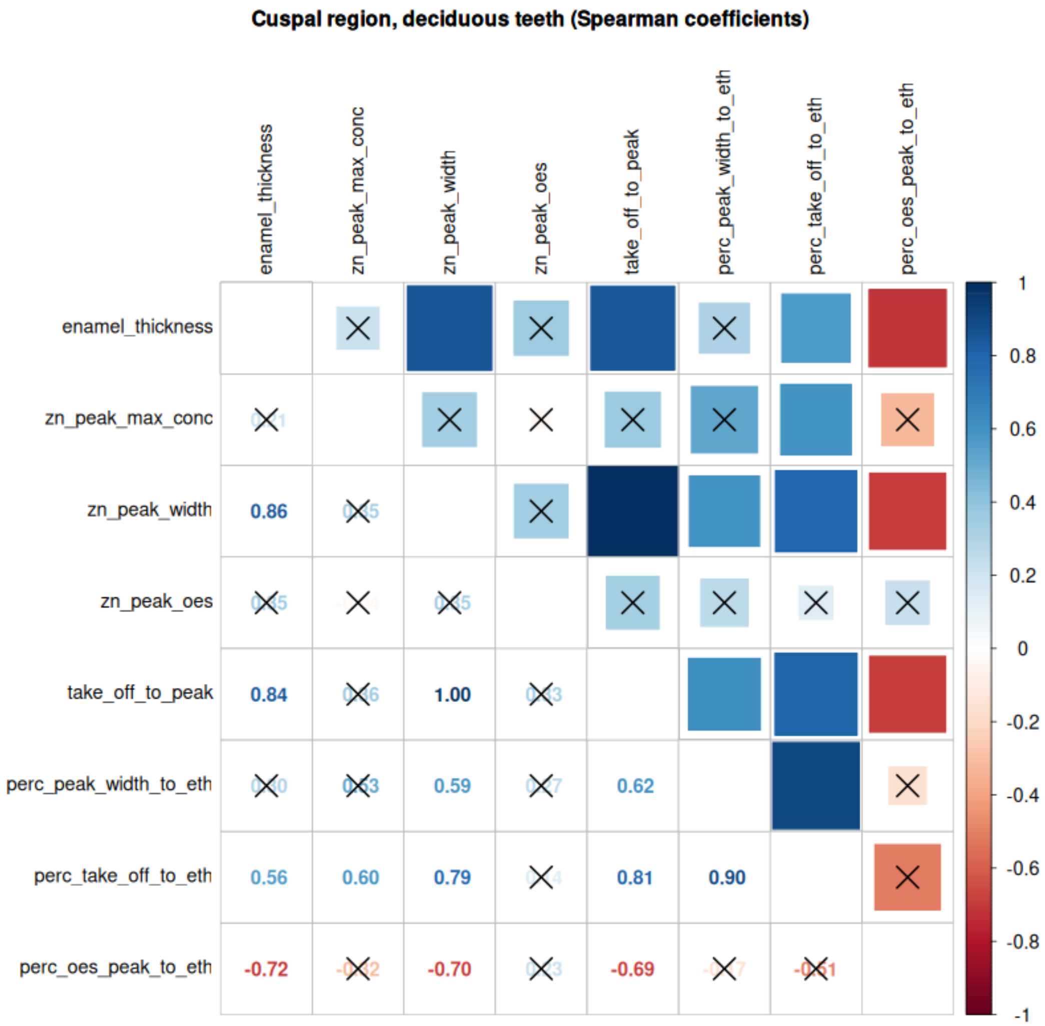

Figure 4: Graphical representation of the correlation matrix among variables for deciduous teeth, measured in the cuspal region.

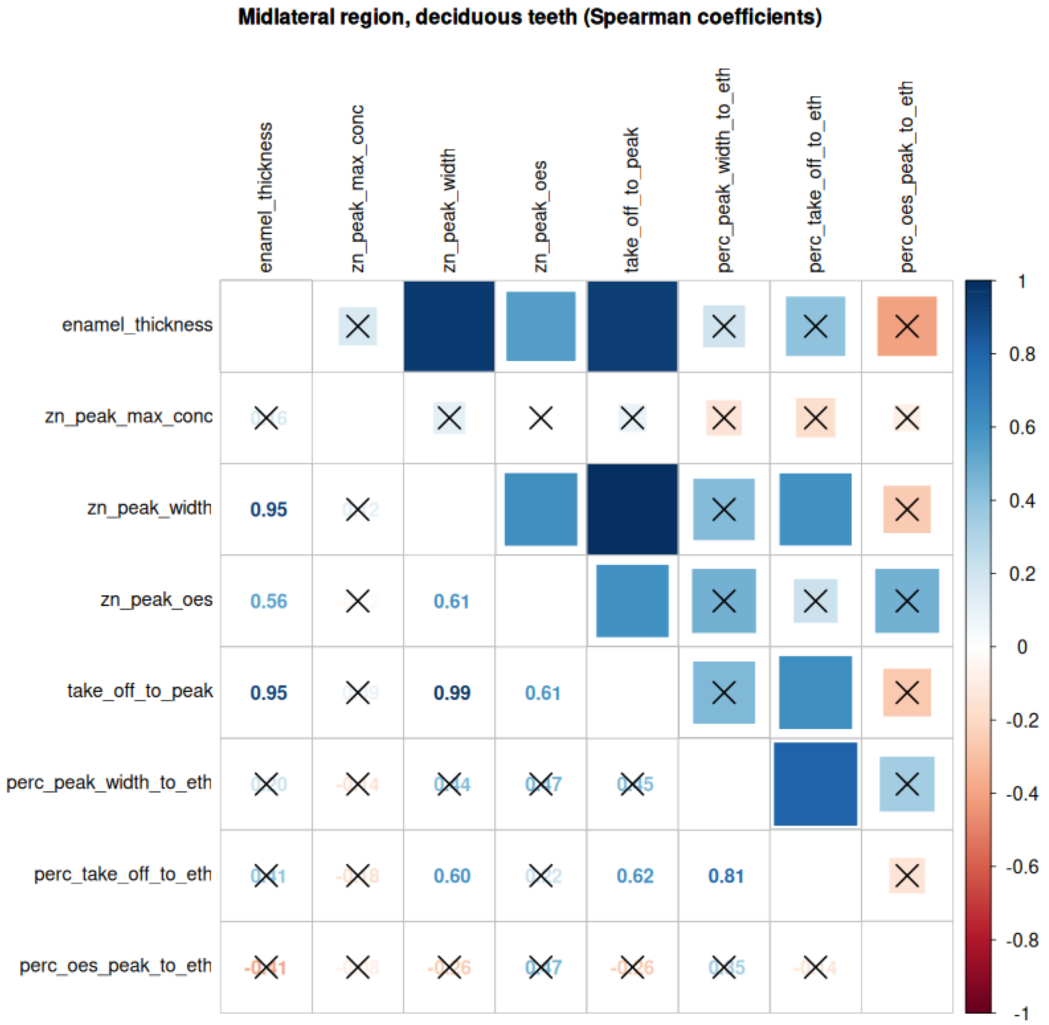

Figure 5: Graphical representation of the correlation matrix among variables for deciduous teeth, measured in the midlateral region.

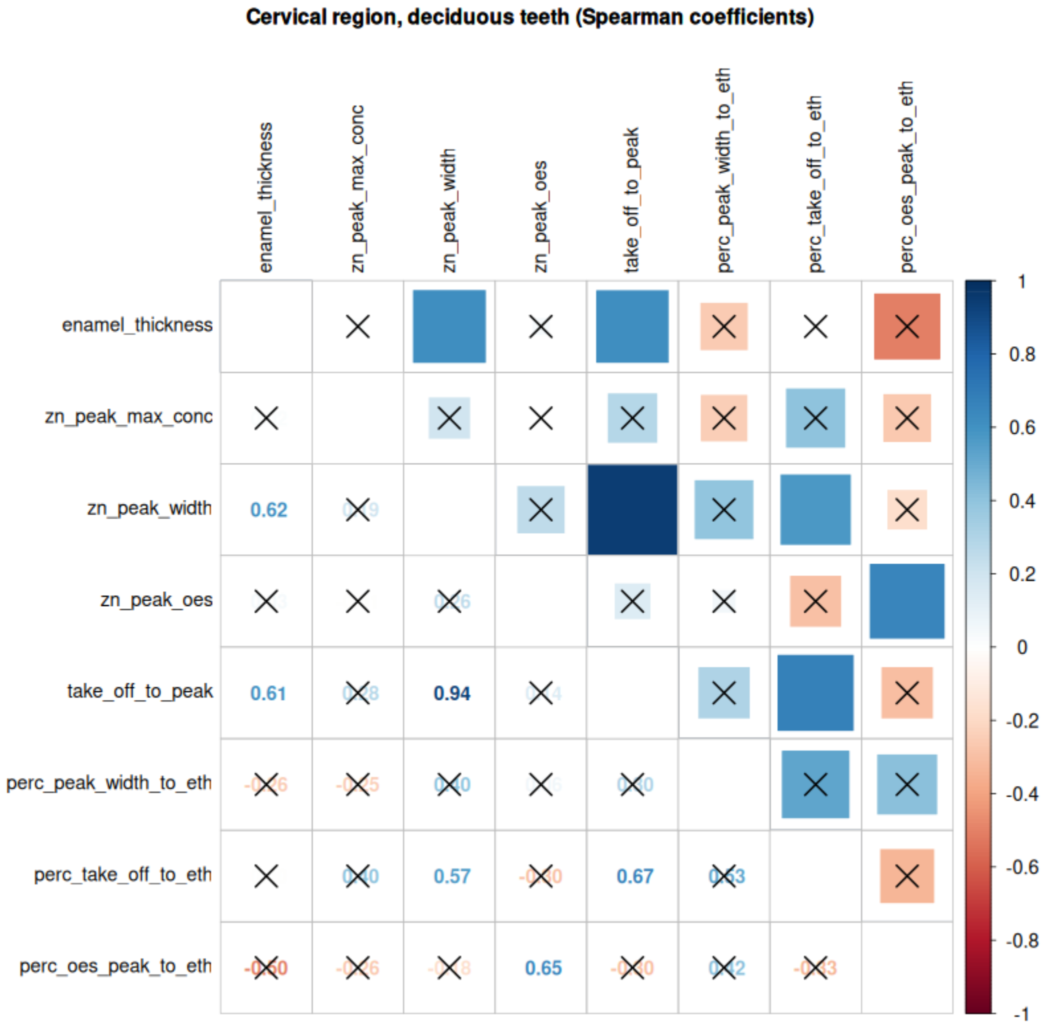

Figure 6: Graphical representation of the correlation matrix among variables for deciduous teeth, measured in the cervical region.

3.3. Permanent teeth

Figures 7 to 9 were computed using only permanent teeth.

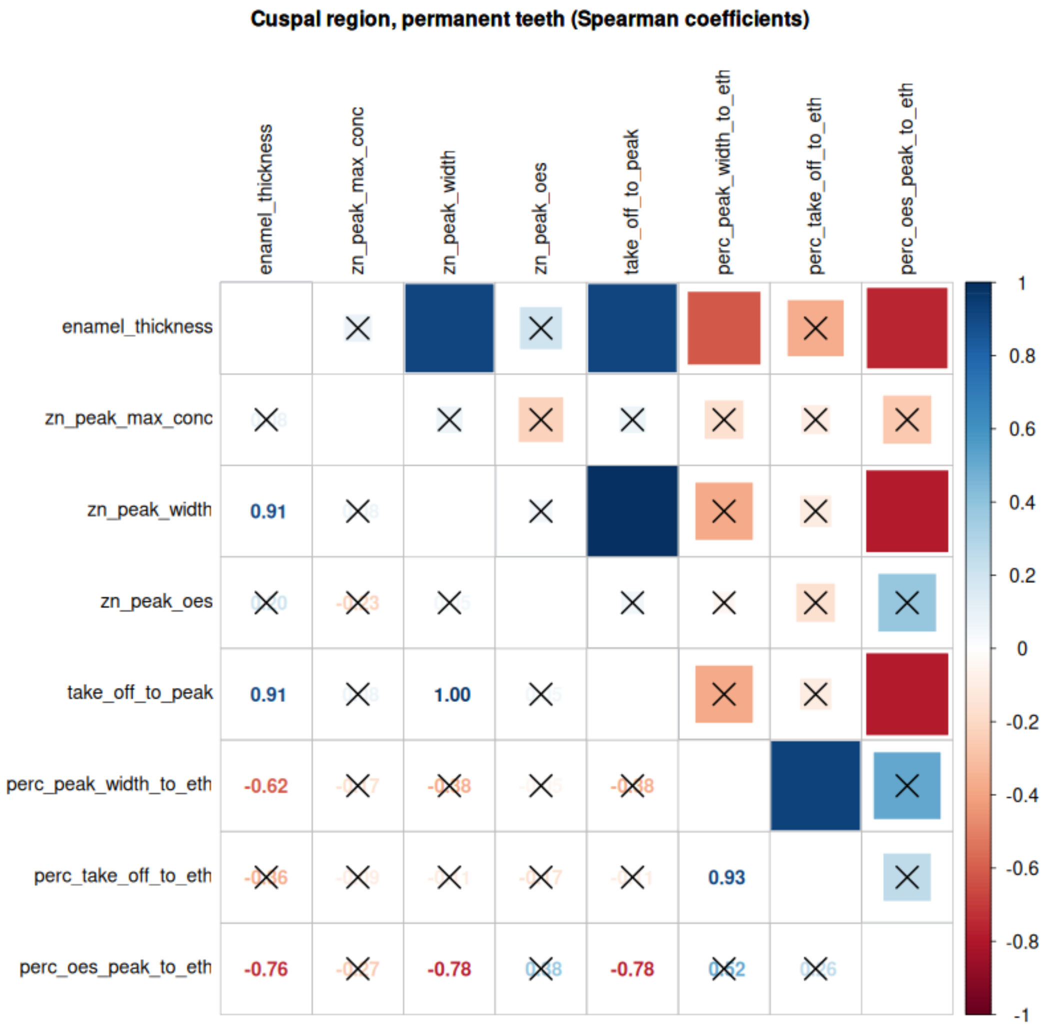

Figure 7: Graphical representation of the correlation matrix among variables for permanent teeth, measured in the cuspal region.

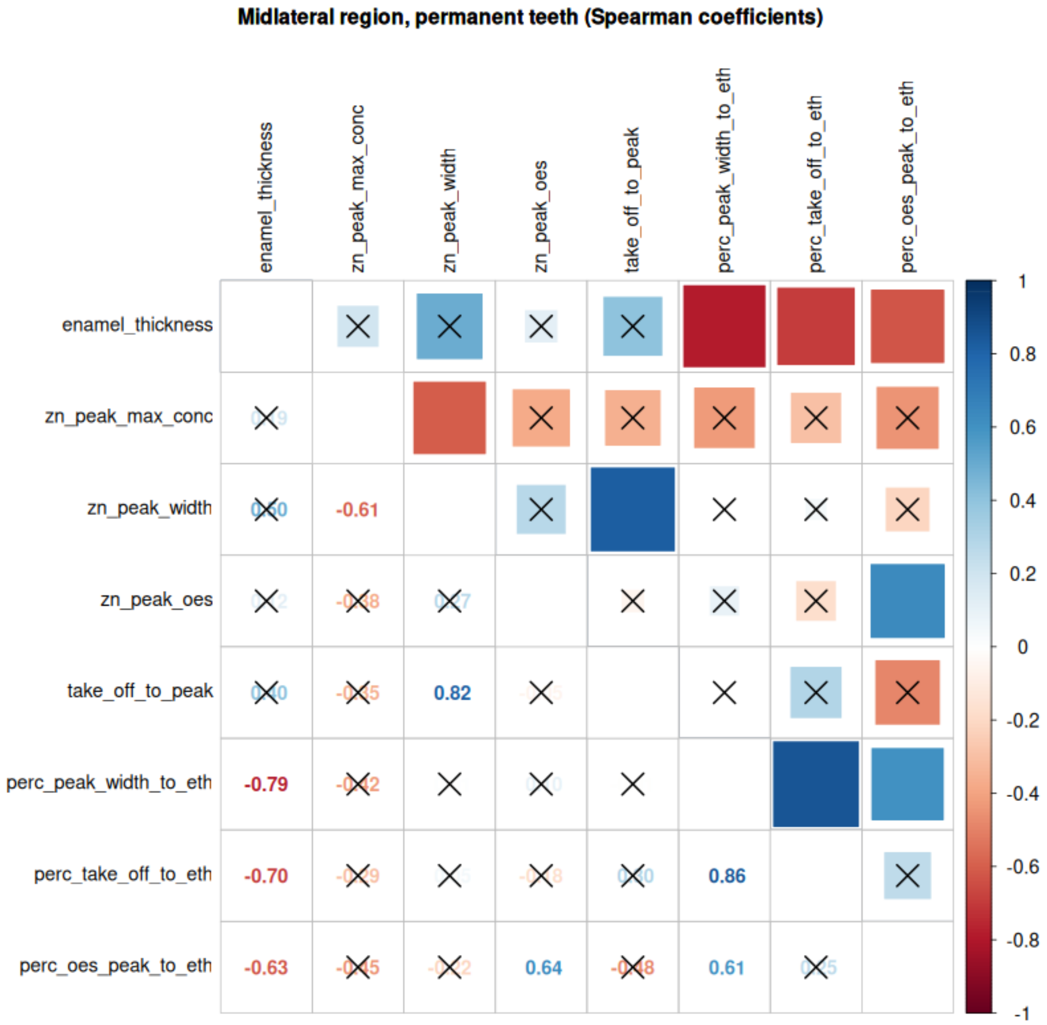

Figure 8: Graphical representation of the correlation matrix among variables for permanent teeth, measured in the midlateral region.

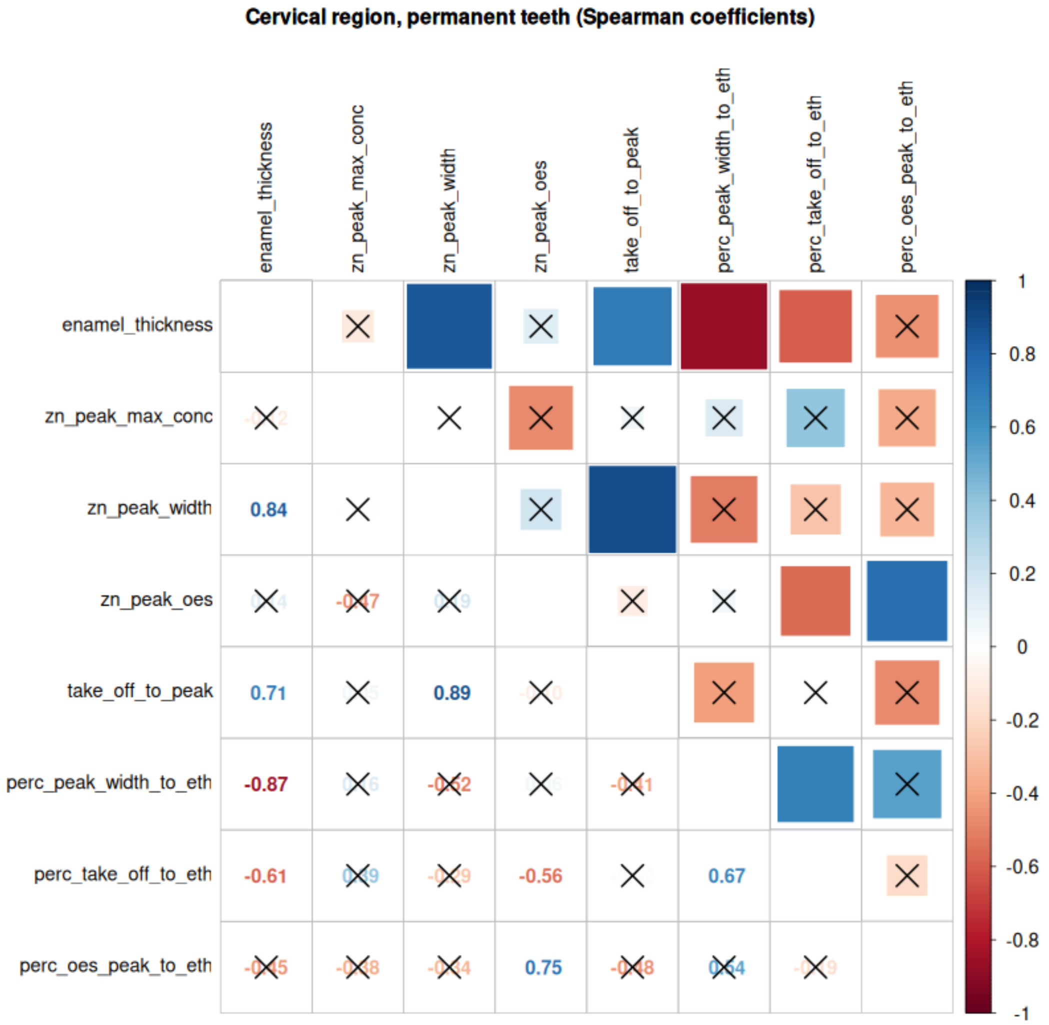

Figure 9: Graphical representation of the correlation matrix among variables for permanent teeth, measured in the cervical region.

4. Scatterplot matrices

To support the previous correlation matrices, we also display (in Figures 10 to 18) matrices of scatterplots (i.e., arrays containing scatterplots for each pair of variables), to identify outliers that might negatively affect the value of the correlation coefficient, or non-linear (or even non-monotone) relationships between variables.

As in the previous case, we display one scatterplot matrix for each tooth type and each enamel region. In addition to the scatterplots, LOESS curves (i.e., local polynomial regression curves) are computed and displayed (in red) for each pair of variables. The name of variables compared in pairs can be read on the diagonal.

4.1. All teeth

Figures 10 to 12 were produced using the whole set of teeth available.

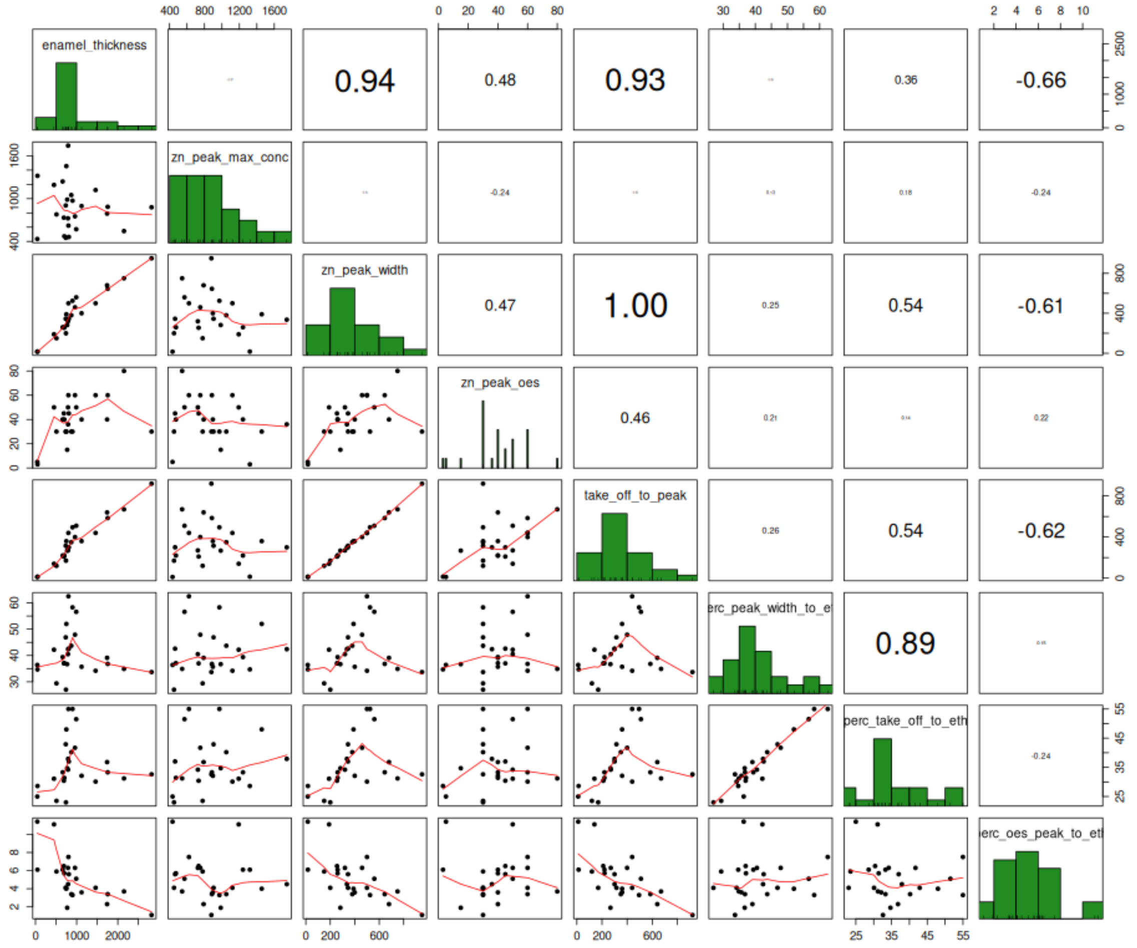

Figure 10: For the cuspal region and all teeth: matrix of scatterplots (with LOESS curves in red) in the bottom part, and Spearman correlation coefficients in the top part.

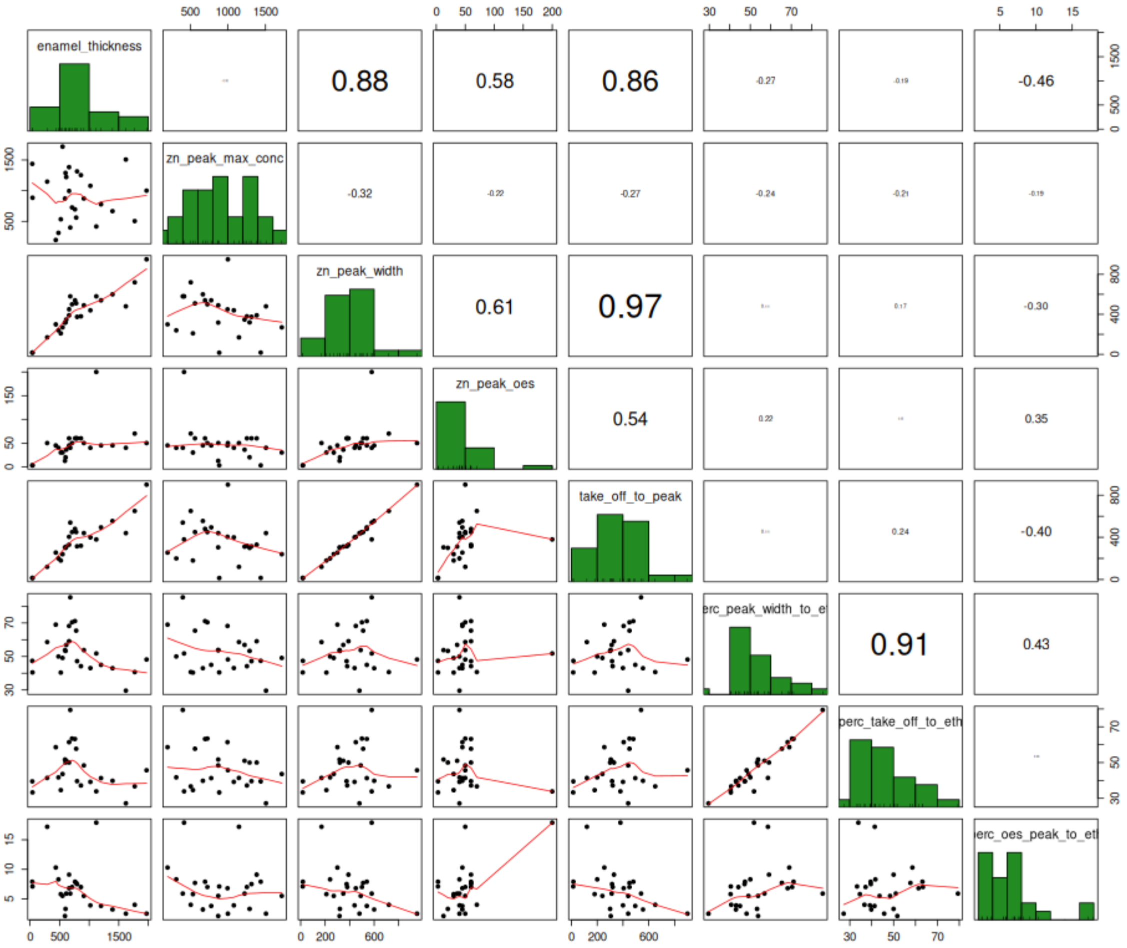

Figure 11: For the midlateral region and all teeth: matrix of scatterplots (with LOESS curves in red) in the bottom part, and Spearman correlation coefficients in the top part.

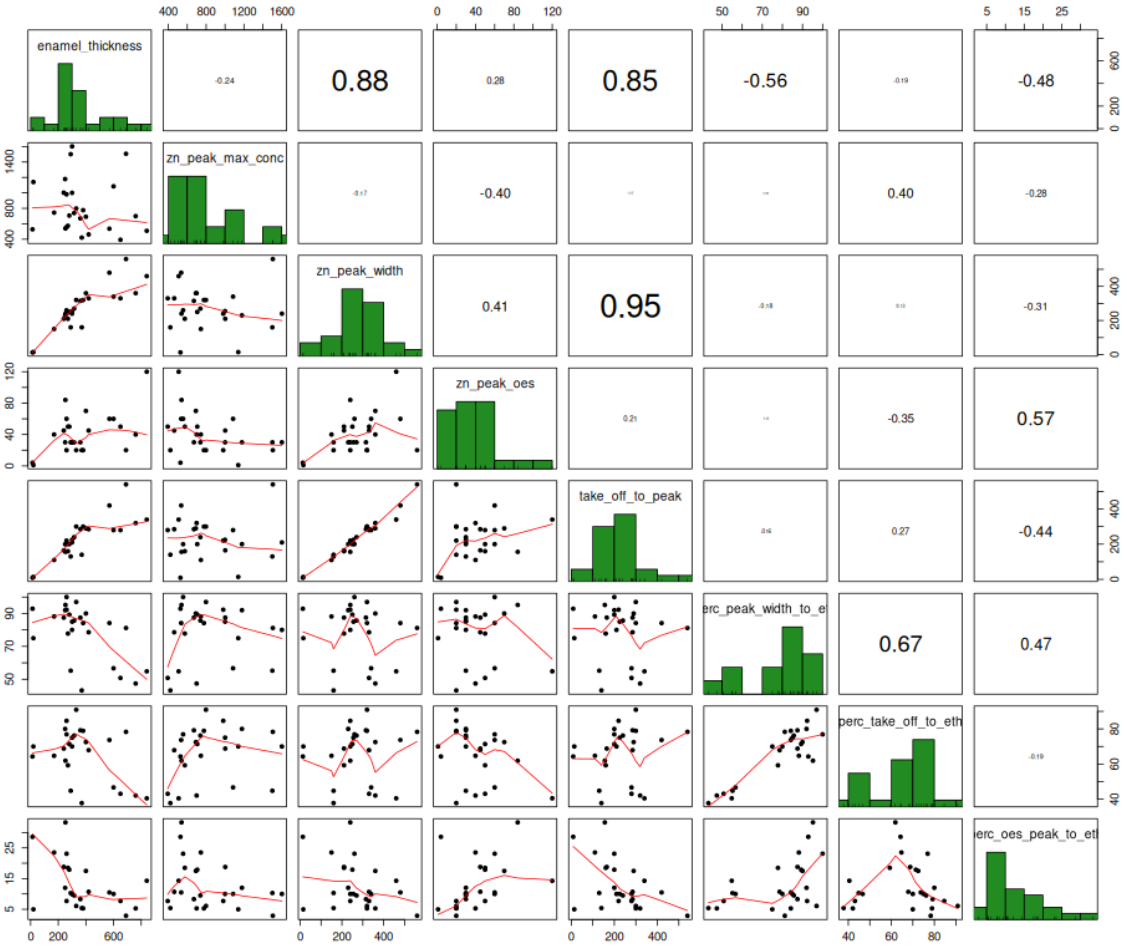

Figure 12: For the cervical region and all teeth: matrix of scatterplots (with LOESS curves in red) in the bottom part, and Spearman correlation coefficients in the top part.

4.2. Deciduous teeth

Figures 13 to 15 were produced using deciduous teeth only.

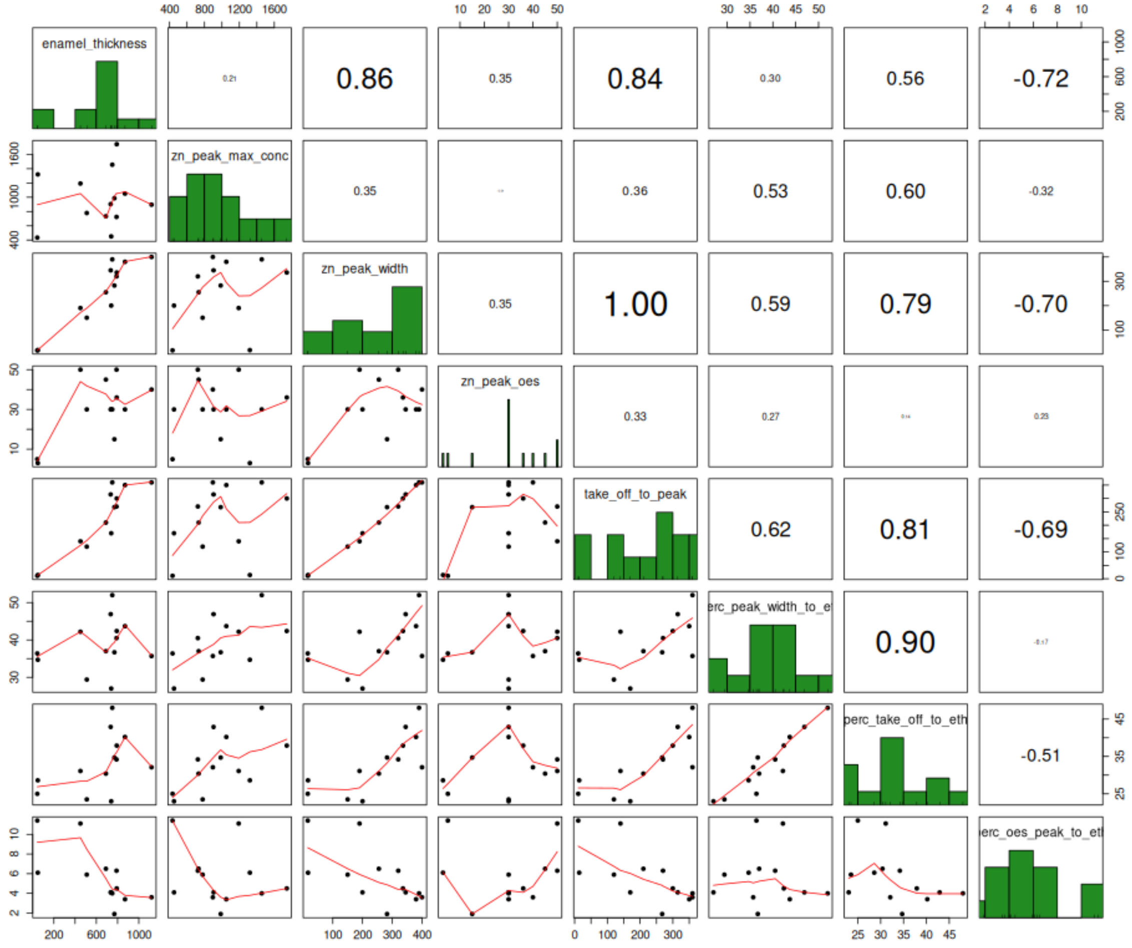

Figure 13: For the cuspal region and deciduous teeth: matrix of scatterplots (with LOESS curves in red) in the bottom part, and Spearman correlation coefficients in the top part.

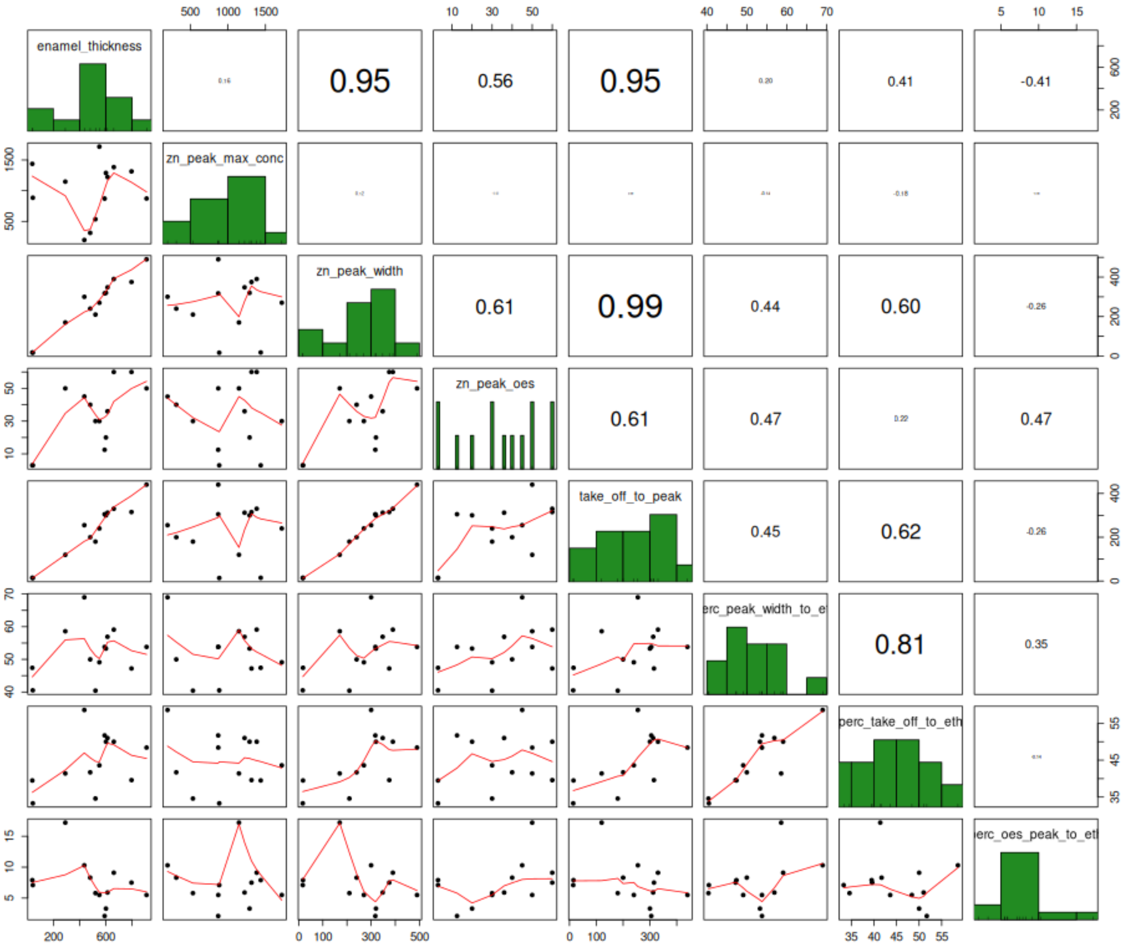

Figure 14: For the midlateral region and deciduous teeth: matrix of scatterplots (with LOESS curves in red) in the bottom part, and Spearman correlation coefficients in the top part.

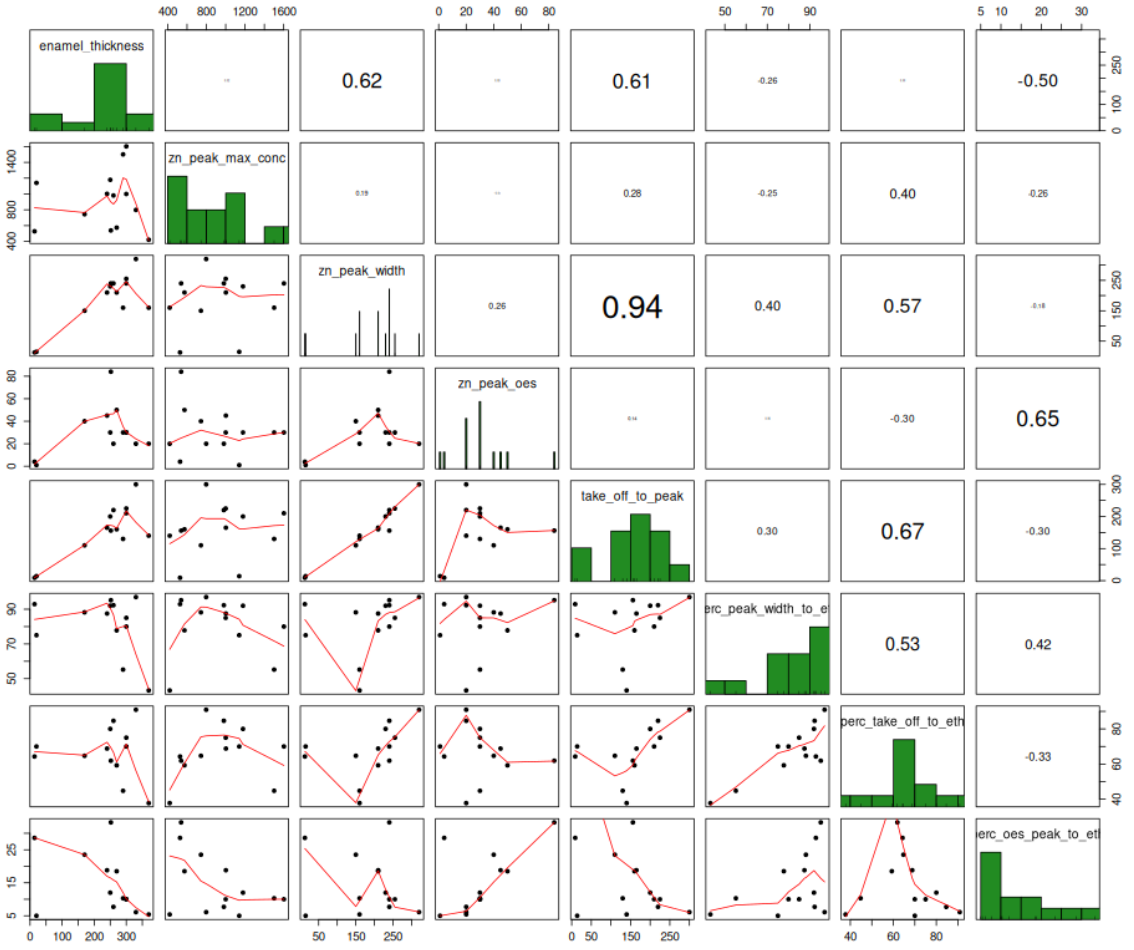

Figure 15: For the cervical region and deciduous teeth: matrix of scatterplots (with LOESS curves in red) in the bottom part, and Spearman correlation coefficients in the top part.

4.3. Permanent teeth

Figures 16 to 18 were produced using permanent teeth only.

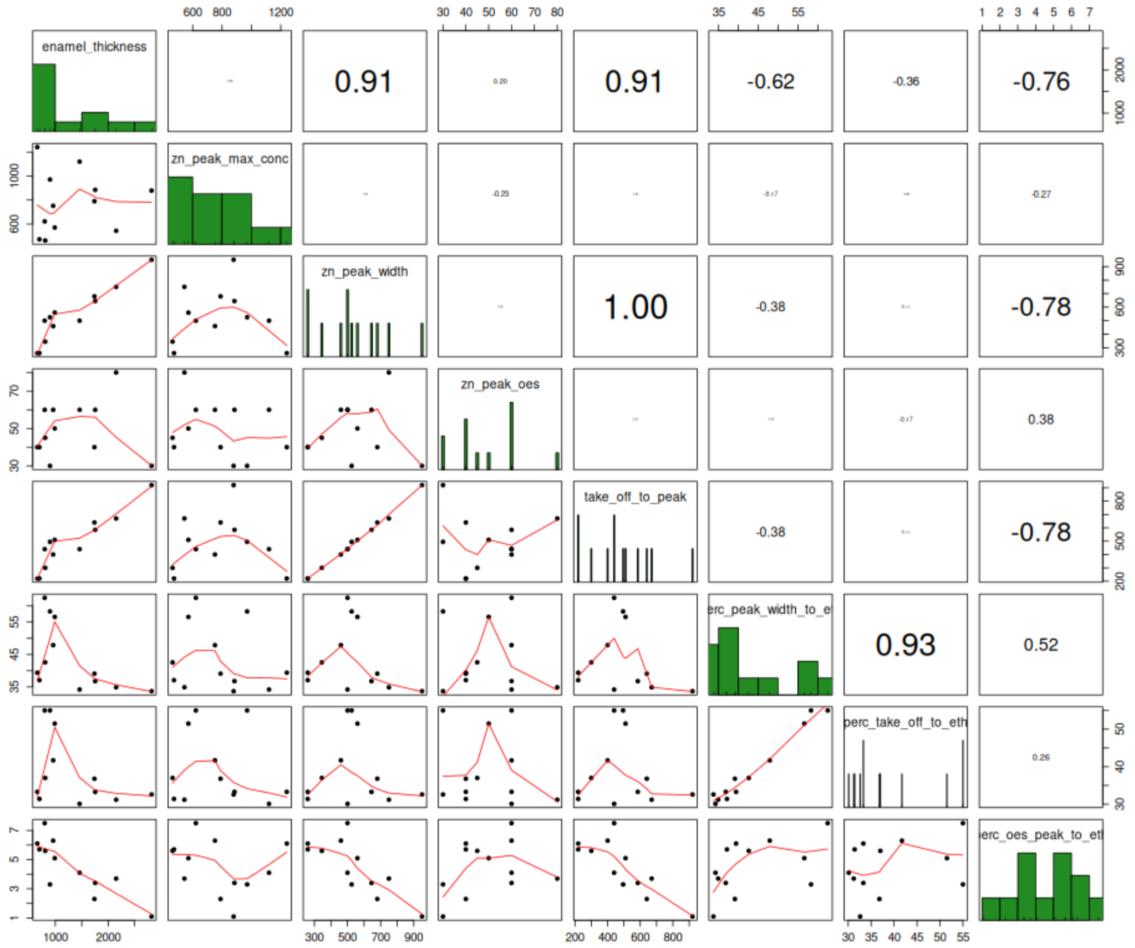

Figure 16: For the cuspal region and permanent teeth: matrix of scatterplots (with LOESS curves in red) in the bottom part, and Spearman correlation coefficients in the top part.

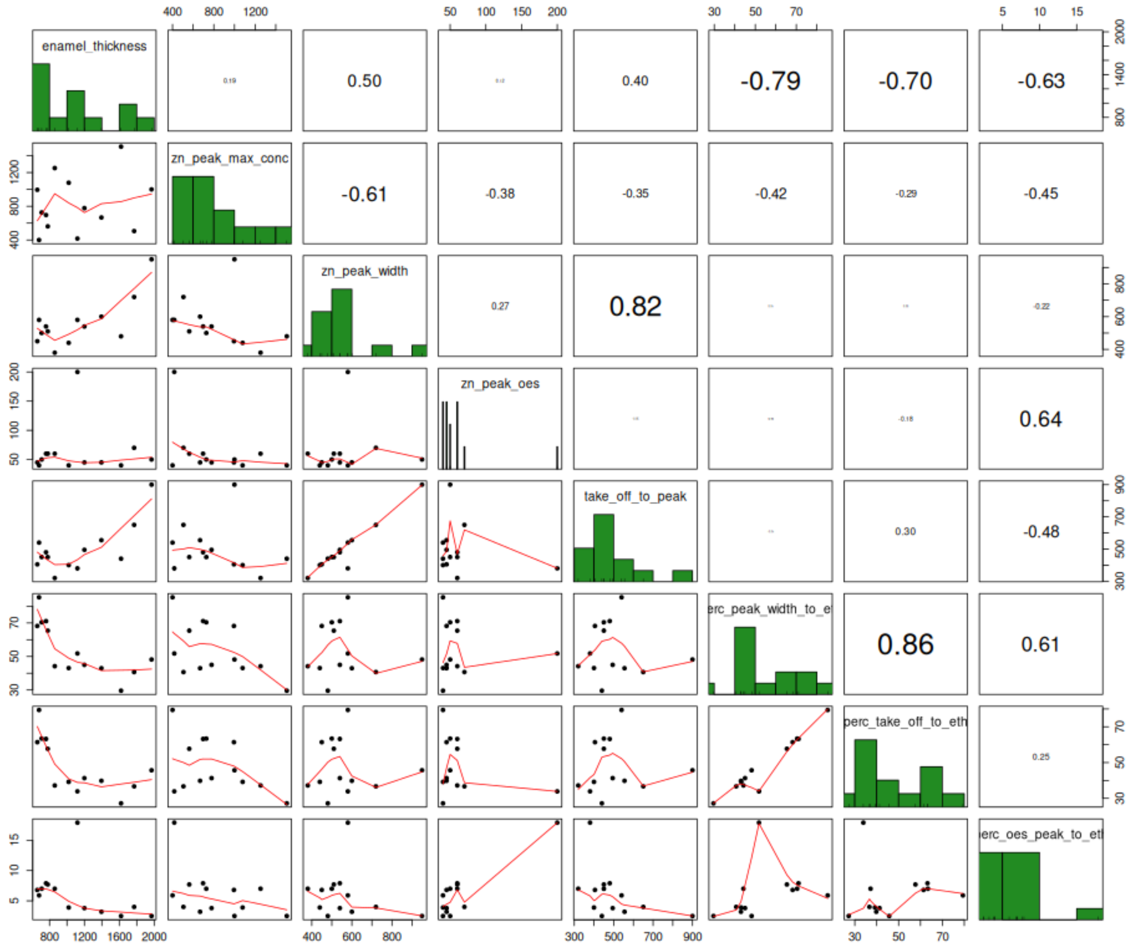

Figure 17: For the midlateral region and permanent teeth: matrix of scatterplots (with LOESS curves in red) in the bottom part, and Spearman correlation coefficients in the top part.

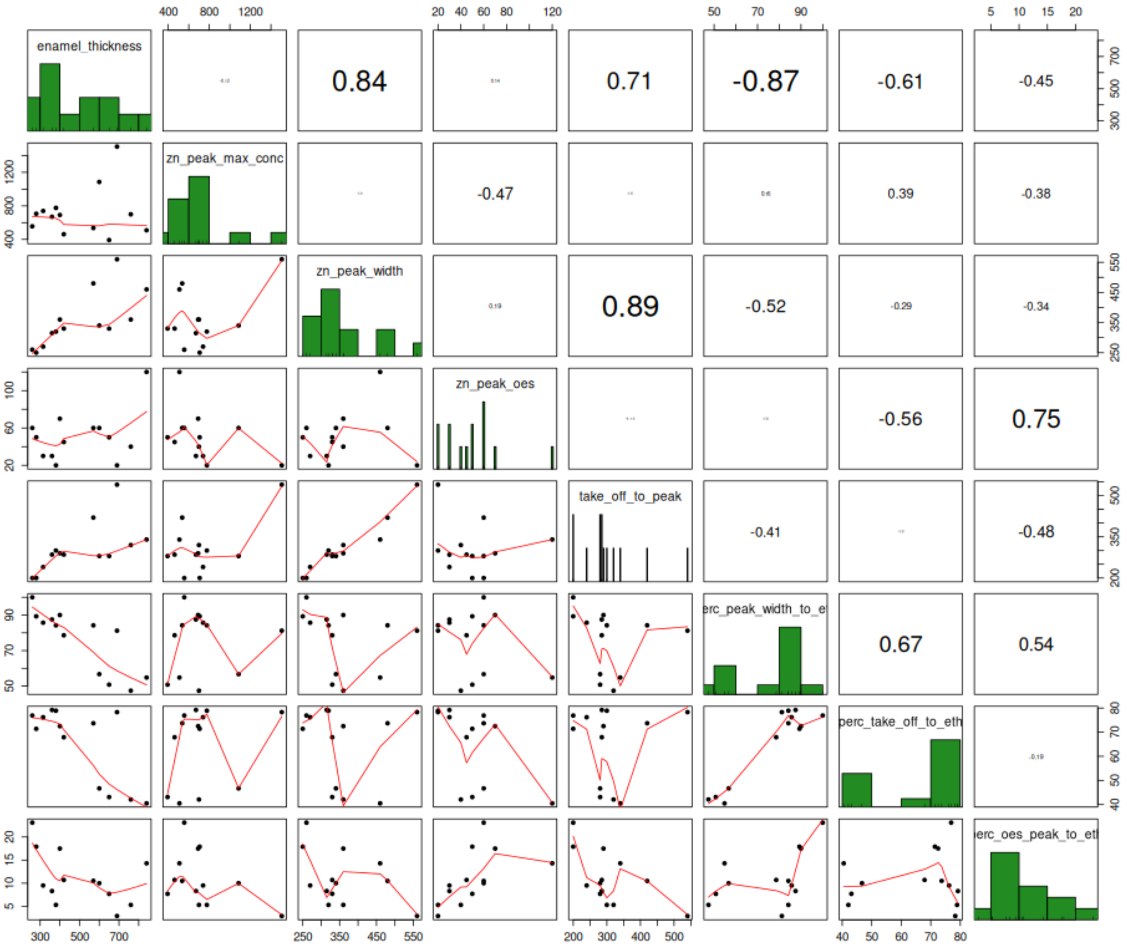

Figure 18: For the cervical region and permanent teeth: matrix of scatterplots (with LOESS curves in red) in the bottom part, and Spearman correlation coefficients in the top part.

5. Principal component analyses

Figures 19 to 23 present various principal component analyses performed on different subsets of the data. Those different subsets may help to avoid the confusing role played by some factors (e.g., tooth type or enamel region), and to inspect whether the global correlation pattern changes across enamel regions or tooth types, in a way which is complementary to the previous plots.

In all PCA plots displayed below, the dotted blue arrows in the correlation circle stand for supplementary (illustrative) variables, i.e. variables which were projected onto the principal axes computed using the other (active) variables.

5.1. By region

Figures 19 to 21 represent PCAs (involving all teeth pooled together) for the cuspal, midlateral and cervical regions respectively.

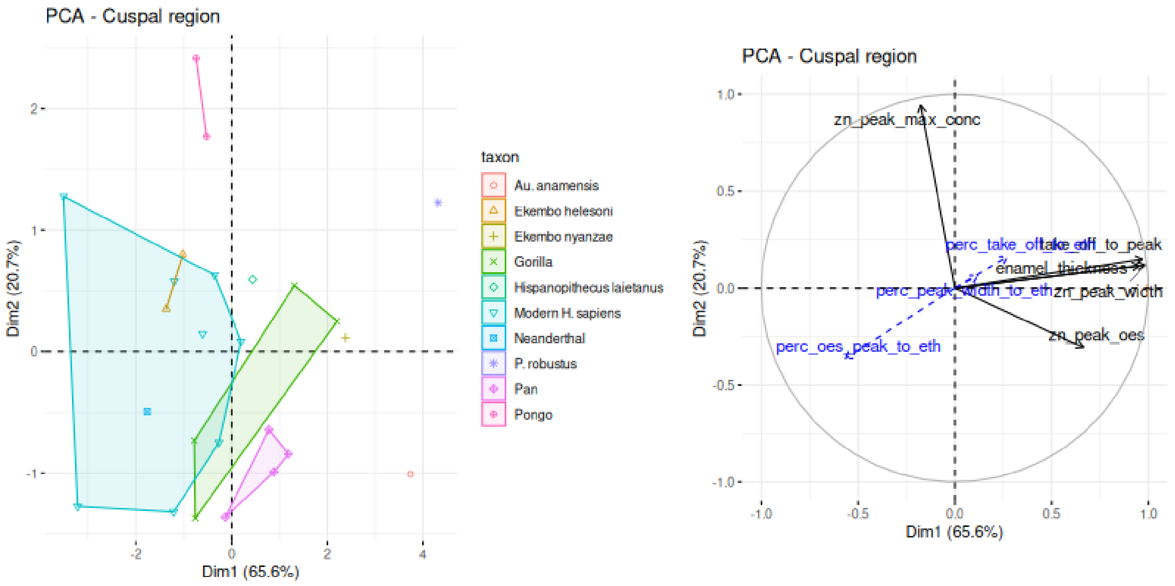

Figure 19: Principal component analysis performed for the cuspal region (all teeth).

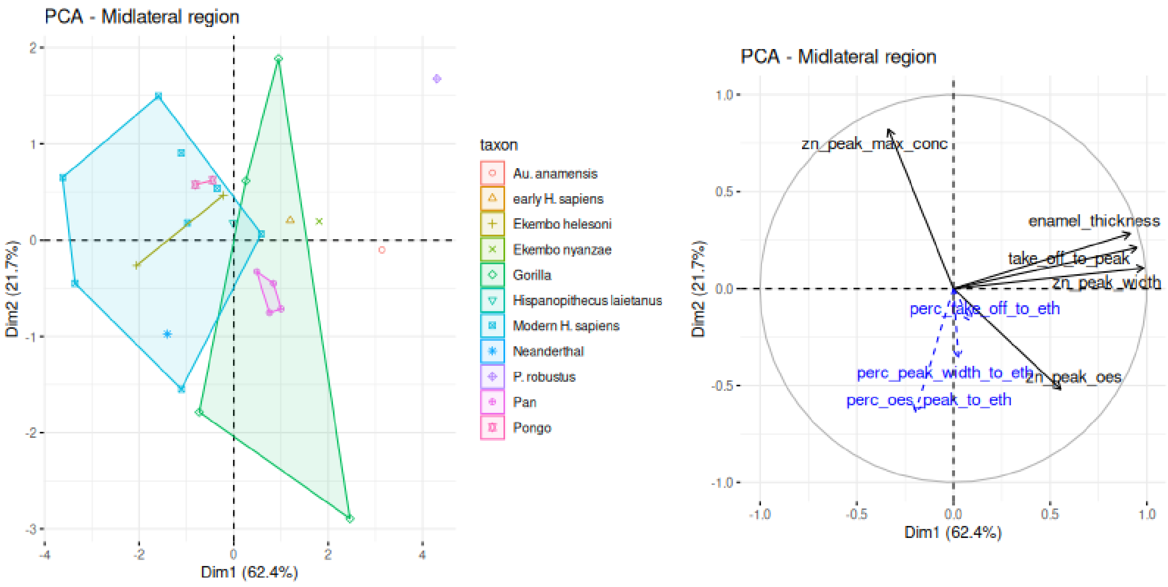

Figure 20: Principal component analysis performed for the midlateral region (all teeth).

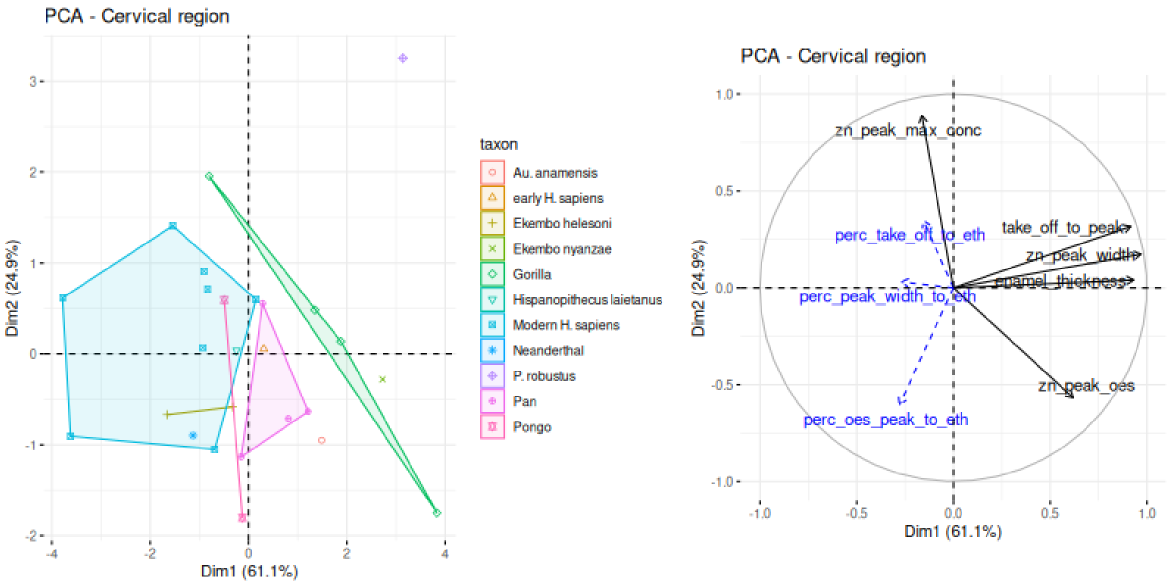

Figure 21: Principal component analysis performed for the cervical region (all teeth).

5.2. By tooth type

Figures 22 to 23 represent PCAs for the deciduous and permanent teeth respectively (all three enamel regions pooled together).

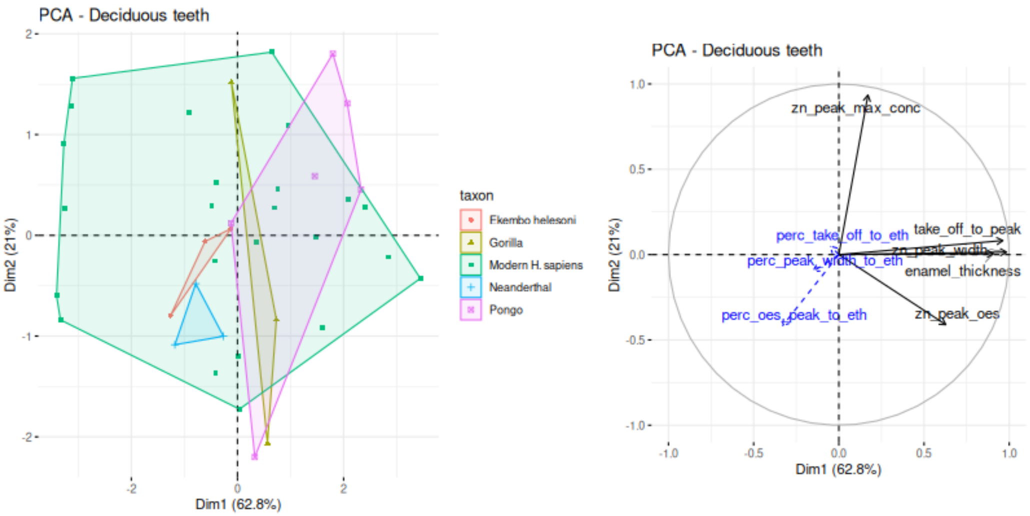

Figure 22: Principal component analysis performed for deciduous teeth only (all enamel regions pooled).

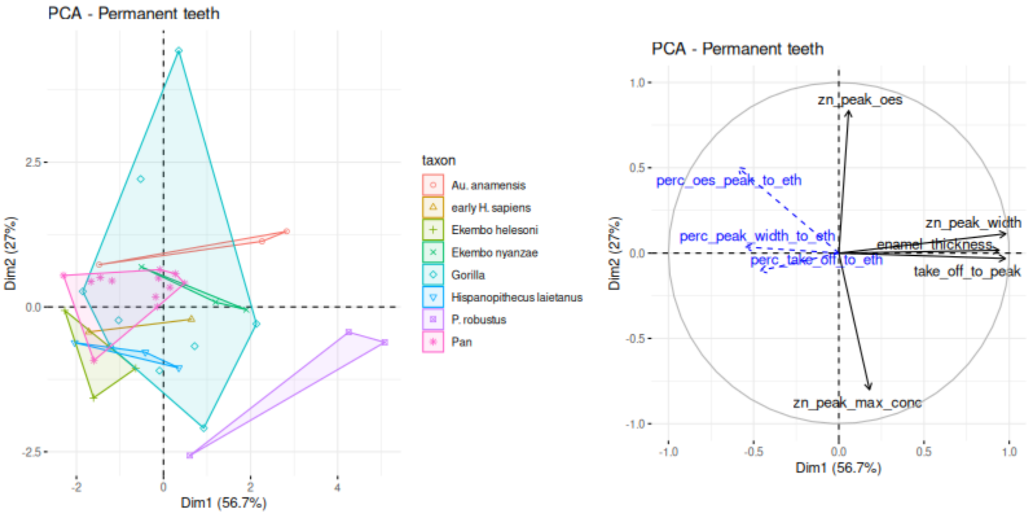

Figure 23: Principal component analysis performed for permanent teeth only (all enamel regions pooled).

6. Formal testing of statistical hypotheses

6.1. Maximal Zn concentration by tooth type

Figure 24 represents the statistical distribution of maximal concentration in zinc for deciduous and permanent teeth. Since the taxa may clearly play a role of confusing factor, they are also explicitly represented.

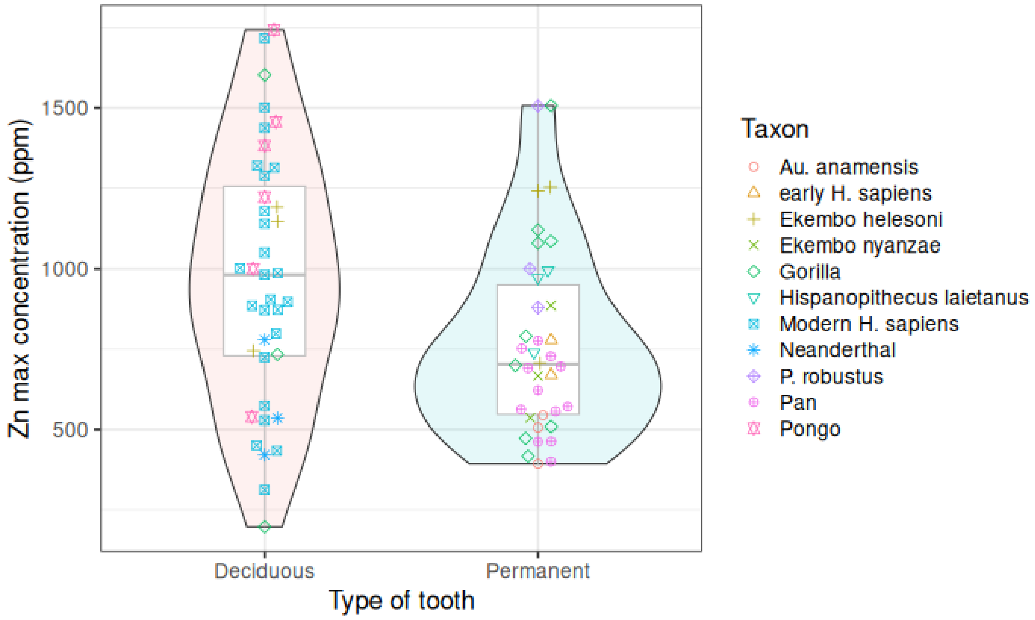

Figure 24: Violin plots, boxplots and (jittered) stripcharts for maximal Zn concentration by tooth type.

In order to test formally the hypothesis that permanent teeth have lower Zn concentration, a Wilcoxon test was performed (please recall once more that taxa may play a role of confusing factor here, so that it is difficult to make a causal inference between tooth type and zinc concentration):

```
        Wilcoxon rank sum test with continuity correction

data:  zn_peak_max_conc by type
W = 983, p-value = 0.01387
alternative hypothesis: true location shift is not equal to 0
95 percent confidence interval:
  37.99996 379.00000
sample estimates:
difference in location
                   210
```

With a p-value approximately equal to 0.01, the Wilcoxon test brings strong evidence against the null hypothesis of no location shift in the probability distributions of Zn maximal concentration between deciduous and permanent teeth.

6.2. Maximal Zn concentration by enamel region

6.2.1. Including all teeth

Figure 25 represents the statistical distribution of maximal concentration in zinc depending on enamel regions.

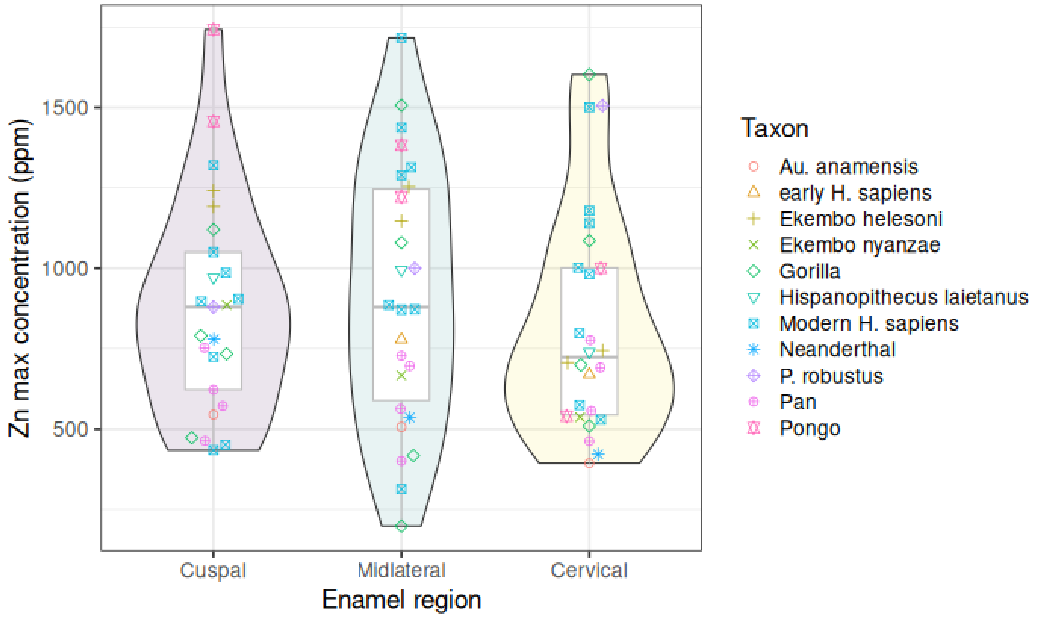

Figure 25: Violin plots, boxplots and (jittered) stripcharts for maximal Zn concentration by enamel region.

Note that the individual "S45 Skhul IV" has one missing value (for the cuspal region), since the tooth cusp is heavily worn. Otherwise, the setting can be seen as a usual case of one-way repeated measures ANOVA, with the zinc concentration as dependent variable, and enamel region as within-subject factor. In order to test formally the hypothesis that Zn concentrations may vary depending on enamel region, we can thus use such a design:

```
## Remove "S45 Skhul IV" to keep a balanced design:
dtf <- dat |>
    dplyr::select(zn_peak_max_conc, specimen_id, enamel_region) |>
    subset(specimen_id != "S45 Skhul IV") |>
    droplevels() |>
    na.omit()

## Perform a one-way repeated mesures ANOVA:
mod <- lmer(
    zn_peak_max_conc ~ enamel_region + (1|specimen_id),
    data = dtf
)
```

```r
## Display the ANOVA table:
Anova(mod, test.statistic = "F")
```

```
Analysis of Deviance Table (Type II Wald F tests with Kenward-Roger df)

Response: zn_peak_max_conc
                   F Df Df.res Pr(>F)
enamel_region 0.6459  2     48 0.5287
```

With a p-value approximately equal 0.52, we can provide no evidence of a difference in Zn maximal concentration depending on enamel region.

6.2.2. Interaction between enamel region and tooth type

As we see in Figure 26, the difference in Zn maximal concentration seems to be consistent from one enamel region to another, with concentrations consisently lower in permanent teeth for all regions. In a mixed-effects two-way ANOVA, this would correspond to a significant effect of the main (fixed) effect of tooth type, with no significant effect for the interaction between enamel region and tooth type.

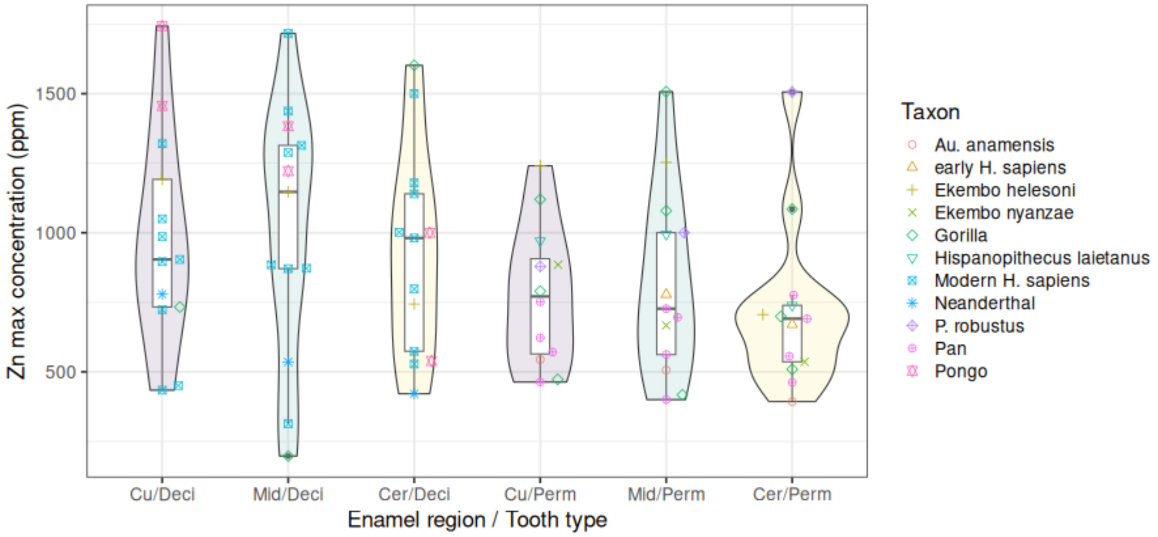

Figure 26: Violin plots, boxplots and (jittered) stripcharts for maximal Zn concentration by enamel region and tooth type.

The analysis of deviance table of such an ANOVA supports indeed our hypotheses:

```r
## Permutational mixed-effects ANOVA:
eza <- ezPerm(
    data = dtf2,
    dv = zn_peak_max_conc,
    wid = specimen_id,
    between = type,
```

```
    within = enamel_region,
    perms = 999
)
print(eza)
```

```
             Effect          p p<.05
1              type 0.04704705     *
2     enamel_region 0.53653654
3 type:enamel_region 0.99599600
```

No evidence of an interaction between tooth type and enamel region could be found ($p \approx 0.99$, see also Fig. 26), thus suggesting that Zn maximal concentrations are consistently lower in permanent teeth, with roughly the same level of decrease between deciduous and permanent teeth in the three anatomical regions.

6.3. Comparisons of correlation coefficients

Some of the correlations previously represented in the correlation matrices (see Section 3) can be compared using the theoretical framework gathered in Diedenhofen and Musch (2015). For instance, in Figure 1 (for the cuspal region and all teeth), the correlation coefficient between `zn_peak_width` and `enamel_thickness` ($r_s \approx 0.94$) seems to be higher than the correlation coefficient between `zn_peak_oes` and `enamel_thickness` ($r_s \approx 0.48$), thus suggesting than the location of the peak has more freedom to vary than its width, depending on enamel thickness. This can be conforted with a formal test[1]:

```
## Comparison of correlation coefficients.
## Test for the cuspal region:
cocor(
    ~zn_peak_width + enamel_thickness | zn_peak_oes + enamel_thickness,
    data = subset(dat, enamel_region == "cu"),
    test = c("hittner2003", "zou2007")
)
```

```

  Results of a comparison of two overlapping correlations based on dependent groups

Comparison between r.jk (enamel_thickness, zn_peak_width) = 0.9388 and r.jh (enamel_thickness, zn_pea
Difference: r.jk - r.jh = 0.4512
Related correlation: r.kh = 0.5491
Data: subset(dat, enamel_region == "cu"): j = enamel_thickness, k = zn_peak_width, h = zn_peak_oes
Group size: n = 25
Null hypothesis: r.jk is equal to r.jh
Alternative hypothesis: r.jk is not equal to r.jh (two-sided)
Alpha: 0.05
```

[1]Formally, note that these tests compare Pearson correlation coefficients, and not the Spearman correlation coefficients represented in Section 3. However, Figure 10 shows that, for the pair of variables tested here, the Pearson correlation coefficient is also relevant as a measure of the strength of the correlation, since there are no strong outliers or nonlinear patterns.

```
hittner2003: Hittner, May, and Silver's (2003) modification of Dunn and Clark's z (1969) using a back
  z = 4.5852, p-value = 0.0000
  Null hypothesis rejected

zou2007: Zou's (2007) confidence interval
  95% confidence interval for r.jk - r.jh: 0.2135 0.8140
  Null hypothesis rejected (Interval does not include 0)
```

The same comparison test can be performed for the midlateral and cervical regions respectively.

```r
## Test for the midlateral region:
cocor(
    ~zn_peak_width + enamel_thickness | zn_peak_oes + enamel_thickness,
    data = subset(dat, enamel_region == "m"),
    test = c("hittner2003", "zou2007")
)
```

```

  Results of a comparison of two overlapping correlations based on dependent groups

Comparison between r.jk (enamel_thickness, zn_peak_width) = 0.8845
  and r.jh (enamel_thickness, zn_peak_oes) = 0.3717
Difference: r.jk - r.jh = 0.5128
Related correlation: r.kh = 0.4527
Data: subset(dat, enamel_region == "m"): j = enamel_thickness,
  k = zn_peak_width, h = zn_peak_oes
Group size: n = 26
Null hypothesis: r.jk is equal to r.jh
Alternative hypothesis: r.jk is not equal to r.jh (two-sided)
Alpha: 0.05

hittner2003: Hittner, May, and Silver's (2003) modification of Dunn and Clark's z (1969)
  using a backtransformed average Fisher's (1921) Z procedure
  z = 3.8167, p-value = 0.0001
  Null hypothesis rejected

zou2007: Zou's (2007) confidence interval
  95% confidence interval for r.jk - r.jh: 0.2316 0.8886
  Null hypothesis rejected (Interval does not include 0)
```

```r
## Test for the cervical region:
cocor(
    ~zn_peak_width + enamel_thickness | zn_peak_oes + enamel_thickness,
    data = subset(dat, enamel_region == "ce"),
    test = c("hittner2003", "zou2007")
)
```

```

  Results of a comparison of two overlapping correlations based on dependent groups
```

```
Comparison between r.jk (enamel_thickness, zn_peak_width) = 0.8557
  and r.jh (enamel_thickness, zn_peak_oes) = 0.5155
Difference: r.jk - r.jh = 0.3402
Related correlation: r.kh = 0.4764
Data: subset(dat, enamel_region == "ce"): j = enamel_thickness,
  k = zn_peak_width, h = zn_peak_oes
Group size: n = 26
Null hypothesis: r.jk is equal to r.jh
Alternative hypothesis: r.jk is not equal to r.jh (two-sided)
Alpha: 0.05

hittner2003: Hittner, May, and Silver's (2003) modification of Dunn and Clark's z (1969)
  using a backtransformed average Fisher's (1921) Z procedure
  z = 2.7054, p-value = 0.0068
  Null hypothesis rejected

zou2007: Zou's (2007) confidence interval
  95% confidence interval for r.jk - r.jh: 0.0925 0.6838
  Null hypothesis rejected (Interval does not include 0)
```

References

Dean, M. C., Garrevoet, J., Van Malderen, S. J. M., Santos, F., Mirazón Lahr, M., Foley, R., & Le Cabec, A. (2023). *Data file for Dean et al.'s article, "The Distribution and Biogenic Origins of Zinc in the Mineralised Tooth Tissues of Modern and Fossil Hominoids: Implications for Life History, Diet and Taphonomy".* Zenodo. doi:10.5281/ZENODO.8403017

Diedenhofen, B., & Musch, J. (2015). Cocor: A Comprehensive Solution for the Statistical Comparison of Correlations. *PLOS ONE*, *10*(4), e0121945. doi:10.1371/journal.pone.0121945

R Core Team. (2023). *R: A language and environment for statistical computing. R Foundation for Statistical Computing, Vienna, Austria.*

Schulte, E., Davison, D., Dye, T., & Dominik, C. (2012). A Multi-Language Computing Environment for Literate Programming and Reproducible Research. *Journal of Statistical Software*, *46*(1), 1–24. doi:10.18637/jss.v046.i03

Simonsohn, U., & Gruson, H. (2021). *Groundhog: Reproducible Scripts via Version-Specific Package Loading.*
