# Supplementary figures and images for: The Distribution and Biogenic Origins of Zinc in the Mineralised Tooth Tissues of Modern and Fossil Hominoids: Implications for Life History, Diet and Taphonomy
Source: Biology (Basel). 2023 Nov 21;12(12):1455. doi: 10.3390/biology12121455 (PMC10740576; doi:10.3390/biology12121455)

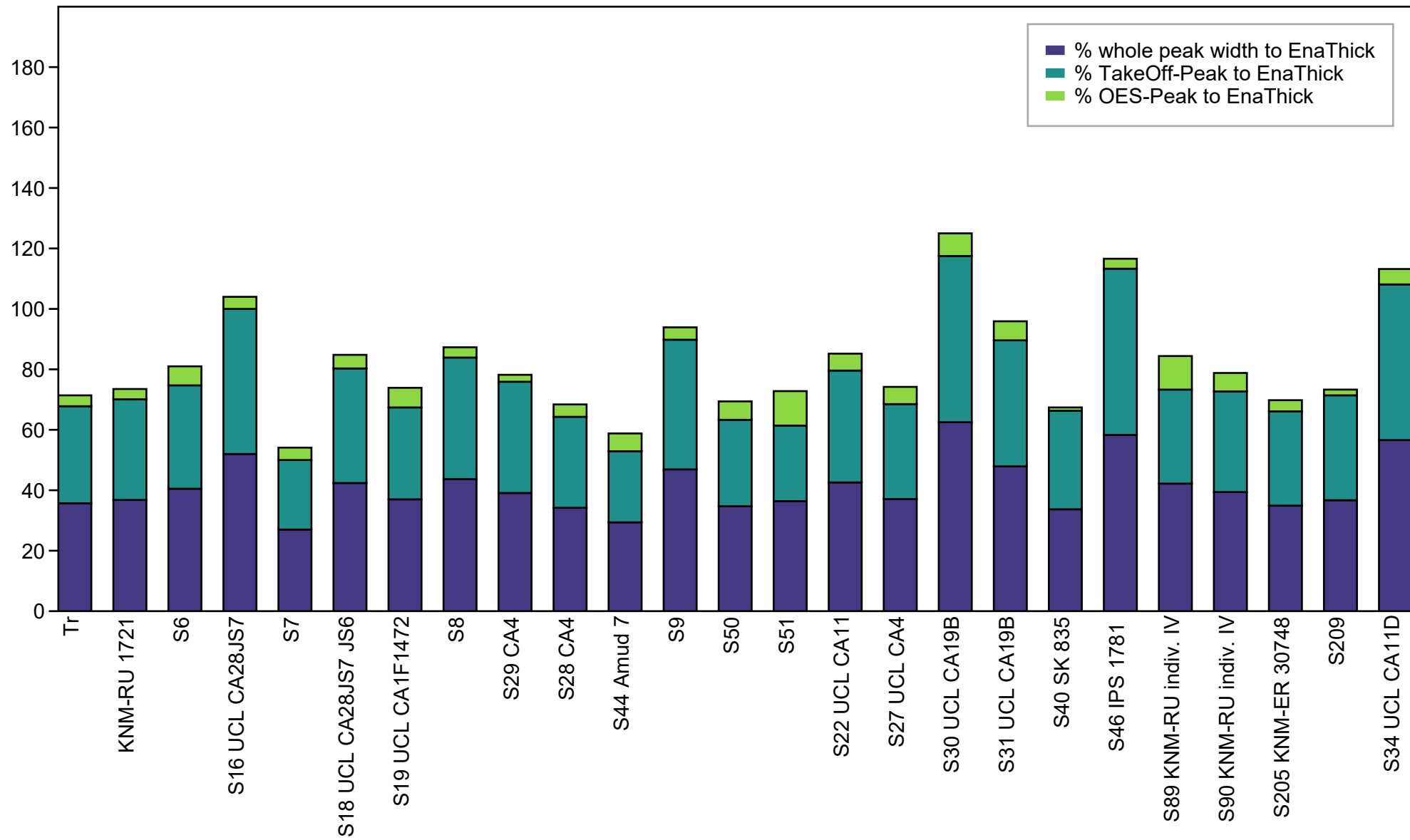

Supplement: Supplementary file 1 [file biology-12-01455-s001.zip › Figure-S1_Cuspal_stacked_chart.pdf]

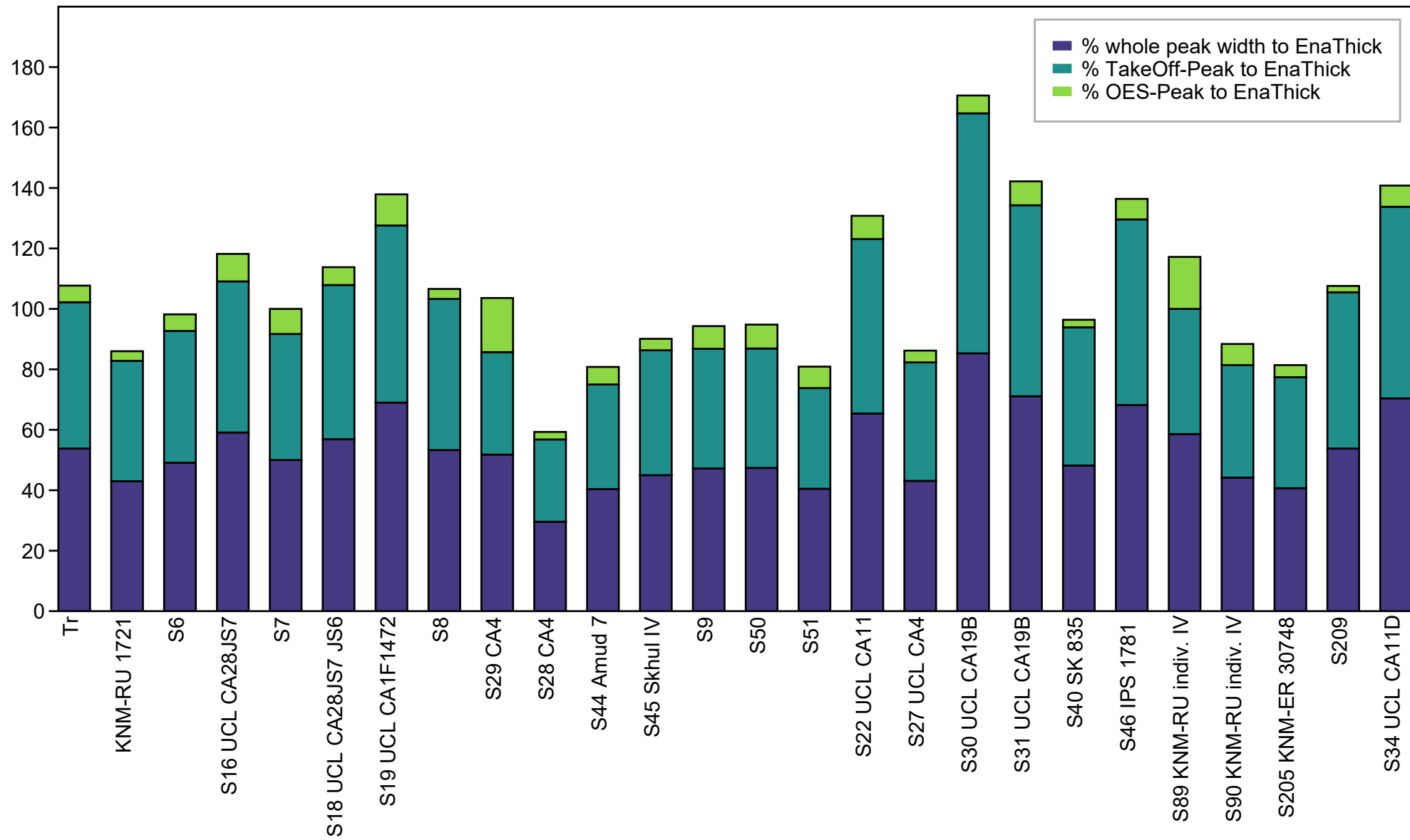

Supplement: Supplementary file 1 [file biology-12-01455-s001.zip › Figure-S2_Midlateral_stacked_chart.pdf]

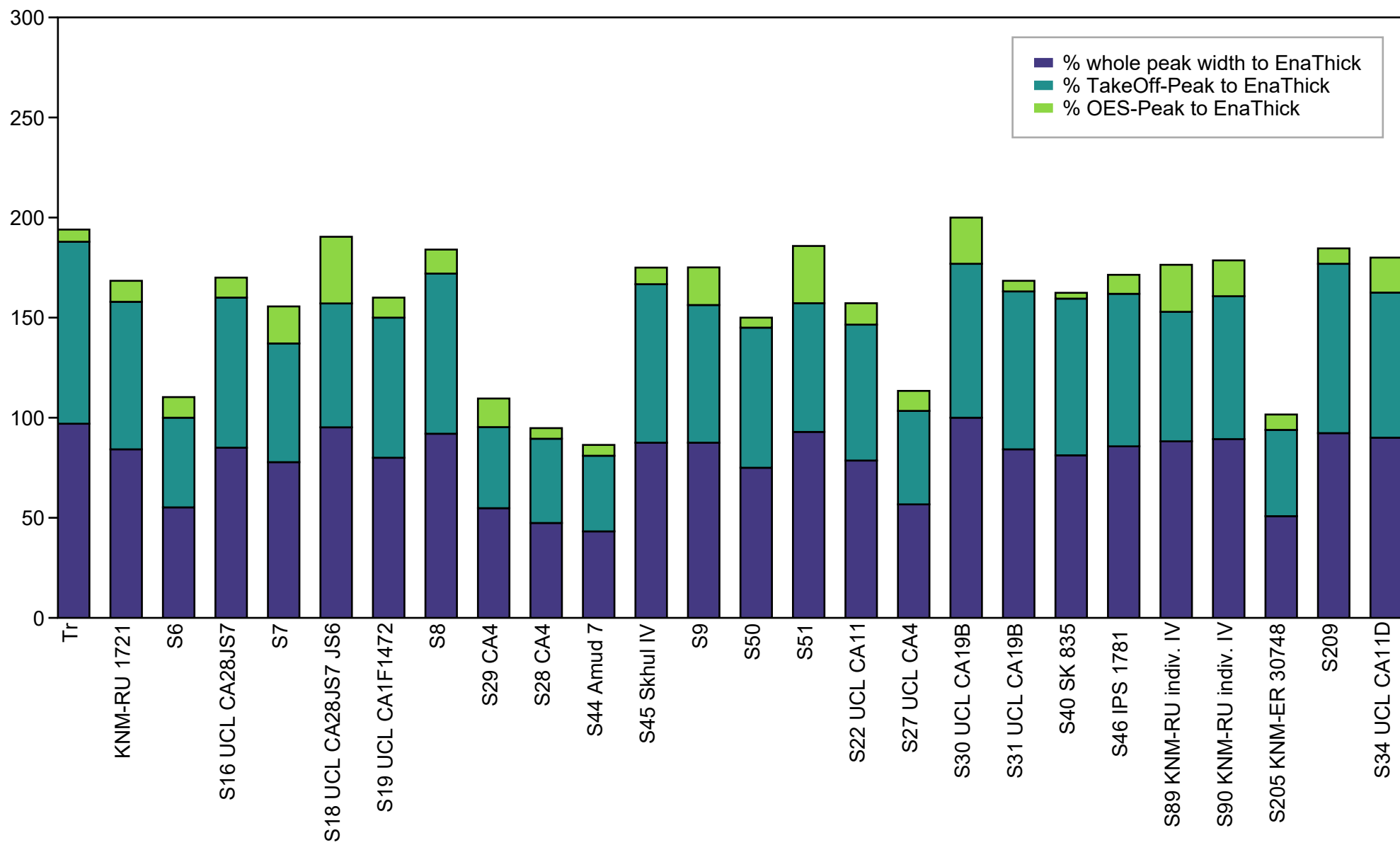

Supplement: Supplementary file 1 [file biology-12-01455-s001.zip › Figure-S3_Cervical_stacked_chart.pdf]

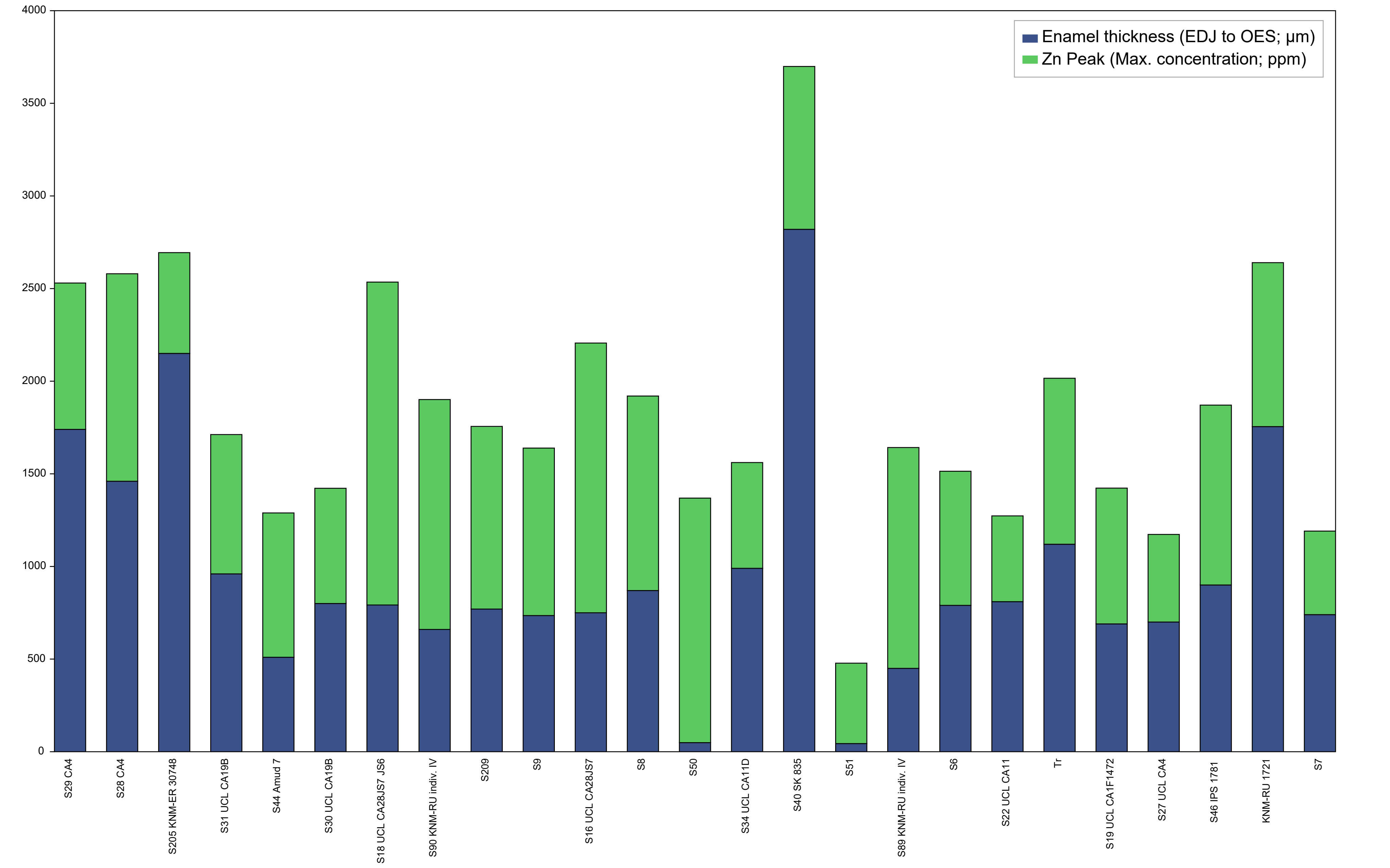

Supplement: Supplementary file 1 [file biology-12-01455-s001.zip › Figure-S4_Cuspal_stacked_chart_Zn_enathick.pdf]

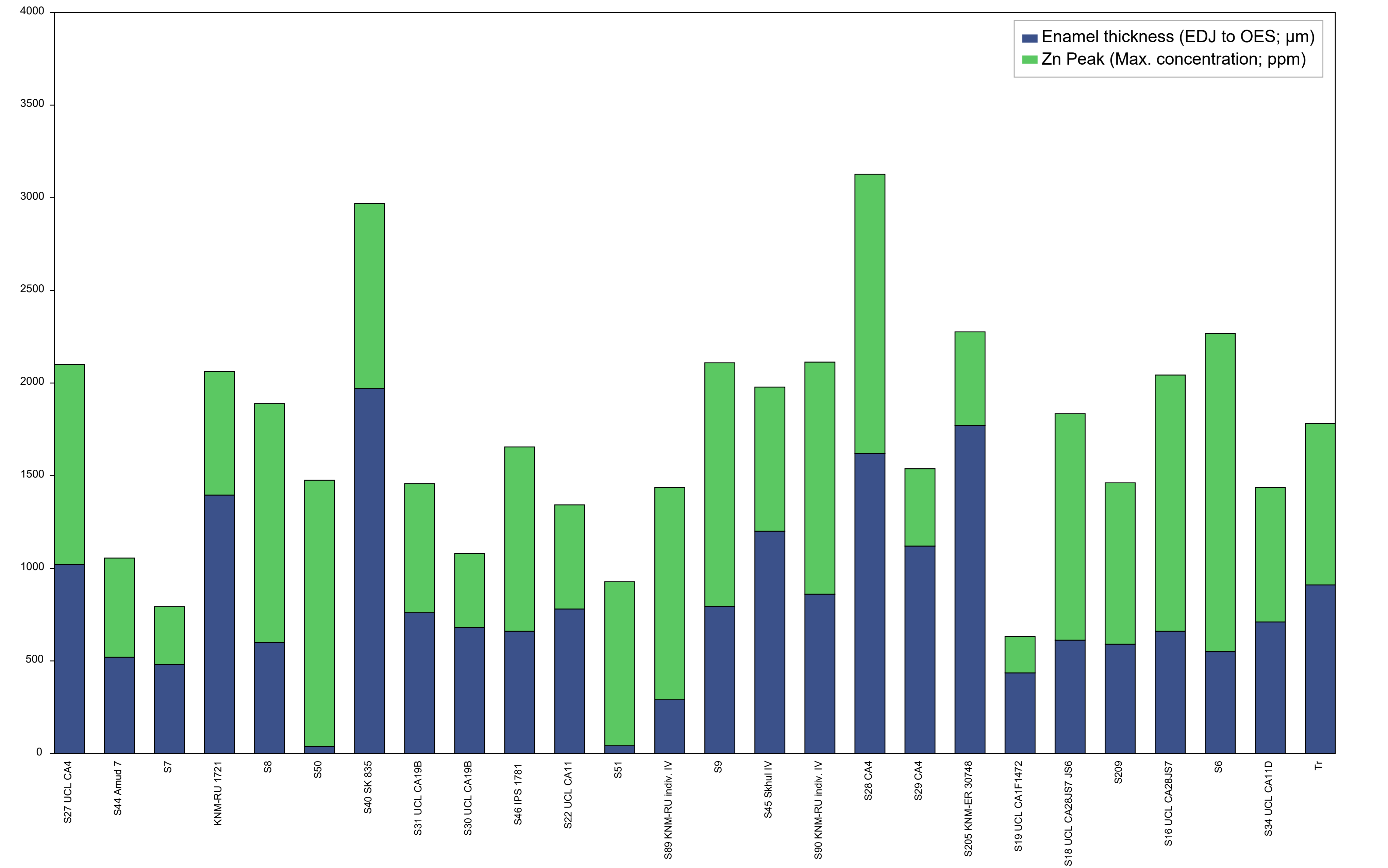

Supplement: Supplementary file 1 [file biology-12-01455-s001.zip › Figure-S5_Midlateral_stacked_chart_Zn_enathick.pdf]

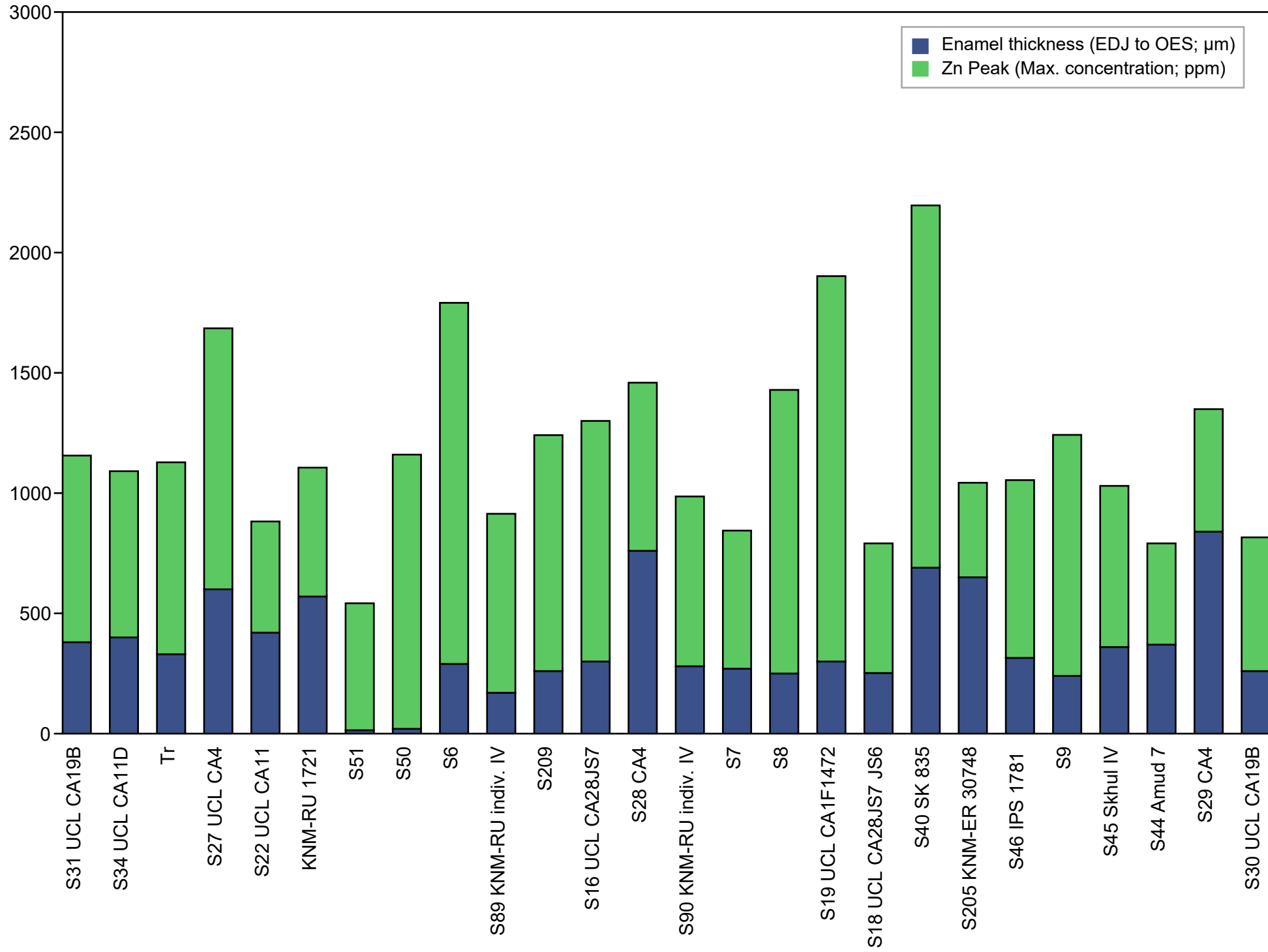

Supplement: Supplementary file 1 [file biology-12-01455-s001.zip › Figure-S6_Cervical_stacked_chart_Zn_enathick.pdf]
